# Comparison of Hepatocellular Carcinoma miRNA Expression Profiling as Evaluated by Next Generation Sequencing and Microarray

**DOI:** 10.1371/journal.pone.0106314

**Published:** 2014-09-12

**Authors:** Yoshiki Murakami, Toshihito Tanahashi, Rina Okada, Hidenori Toyoda, Takashi Kumada, Masaru Enomoto, Akihiro Tamori, Norifumi Kawada, Y-h Taguchi, Takeshi Azuma

**Affiliations:** 1 Department of Hepatology, Osaka City University Graduate School of Medicine, Osaka, Japan; 2 Department of Medical Pharmaceutics, Kobe Pharmaceutical University, Kobe, Japan; 3 Division of Gastroenterology, Department of Internal Medicine, Kobe University Graduate School of Medicine, Kobe, Japan; 4 Department of Gastroenterology, Ogaki Municipal Hospital, Ogaki, Japan; 5 Department of Physics, Chuo University, Tokyo, Japan; New York University School of Medicine, United States of America

## Abstract

MicroRNA (miRNA) expression profiling has proven useful in diagnosing and understanding the development and progression of several diseases. Microarray is the standard method for analyzing miRNA expression profiles; however, it has several disadvantages, including its limited detection of miRNAs. In recent years, advances in genome sequencing have led to the development of next-generation sequencing (NGS) technologies, which significantly advance genome sequencing speed and discovery. In this study, we compared the expression profiles obtained by next generation sequencing (NGS) with the profiles created using microarray to assess if NGS could produce a more accurate and complete miRNA profile. Total RNA from 14 hepatocellular carcinoma tumors (HCC) and 6 matched non-tumor control tissues were sequenced with Illumina MiSeq 50-bp single-end reads. Micro RNA expression profiles were estimated using miRDeep2 software. As a comparison, miRNA expression profiles for 11 out of 14 HCCs were also established by microarray (Agilent human microRNA microarray). The average total sequencing exceeded 2.2 million reads per sample and of those reads, approximately 57% mapped to the human genome. The average correlation for miRNA expression between microarray and NGS and subtraction were 0.613 and 0.587, respectively, while miRNA expression between technical replicates was 0.976. The diagnostic accuracy of HCC, p-value, and AUC were 90.0%, 7.22×10^−4^, and 0.92, respectively. In summary, NGS created an miRNA expression profile that was reproducible and comparable to that produced by microarray. Moreover, NGS discovered novel miRNAs that were otherwise undetectable by microarray. We believe that miRNA expression profiling by NGS can be a useful diagnostic tool applicable to multiple fields of medicine.

## Introduction

MicroRNAs (miRNAs) are an abundant class of small (19–24 nt) and highly conserved, non-coding RNA. They act as post-transcriptional regulators of gene expression, altering mRNA transcription and translation by hybridizing to the untranslated regions (UTRs) of certain subsets of mRNAs [1] [2]. Since their initial discovery in *Caenorhabditis elegans* in 1993 [3], researchers have gained much insight into the prevalence of miRNAs in other species. The latest miRBase database (release 20) contains 1827 precursor miRNAs and 2578 mature miRNA products in *Homo sapiens* (http://www.mirbase.org/index.shtml).

Hepatocellular carcinoma (HCC) is a common cause of cancer-related deaths worldwide. There are more than 250,000 new HCC cases and an estimated 500,000–600,000 HCC deaths annually [4] [5]. The most frequent etiology of HCC is chronic hepatitis B and C (CHB, CHC), or alcoholic liver disease. Although recent advances in functional genomics provide a deeper understanding of viral associated hepatocarcinogenesis (review in [6]), the molecular pathogenesis of HCC remains unclear.

Altered miRNA expression has been observed in a large variety of HCC and a correlation has been found between miRNA expression and histological differentiation [7] [8]. For example, the expression level of miR-26 has been associated with hepatocarcinogenesis and response to interferon therapy [9]. Moreover recently, miR-122 expression was associated with hepatocarcinogenesis, liver homeostasis, and essential liver metabolism [10] [11]. miR-18 has also been highly associated with the occurrence and progression of different types of cancer [12] [13]. In other research, miRNA expression profiles were associated with vascular invasion, the levels of alpha-fetoprotein, and large tumor size [14].

To date, studies exploring the role of miRNAs in hepatocarcinogenesis have relied on microarrays to assay miRNA expression. Deep sequencing, a set of technologies that produce large amounts of sequence data from nucleic acid specimens, is rapidly replacing microarrays as the technology of choice for quantifying and annotating miRNAs [15] [16]. Deep sequencing has the superior ability to capture the scale and complexity of whole transcriptomes [17]. In particular, short read deep sequencing (e.g., the Illumina MiSeq platform) is appropriate for miRNAs because a complete miRNA can be sequenced with a single read. While array design relies on prior knowledge of the miRNAs being investigated, deep sequencing allows for the discovery of novel miRNAs. Furthermore, microarray methods lack the dynamic range to detect and quantify low abundance transcripts, but deep sequencing can identify miRNAs that are expressed at levels that fall below microarray's detectable threshold. In addition, deep sequencing eliminates background problems that result from cross-hybridization in microarrays, thus facilitating interpretation of the signal and obviating the non-linear data manipulation steps required by microarrays. Therefore, the application of deep sequencing to miRNA profiling has the potential to uncover novel miRNAs and to detect expression of rare but functionally significant miRNAs. Recently, deep sequencing was used to analyze non-coding RNAs in HCC, by which miRNAs, PIWI-interacting RNA, and small nucleolar RNAs were identified [18].

In this study, we created miRNA expression profiles for HCC and non-tumorous tissue using NGS. We then compared the miRNA expression profiles obtained by NGA and microarray. Unlike previous studies, we sequenced un-pooled miRNA libraries to a previously unprecedented sequencing depth from multiple replicates and controls across multiple time-points, allowing us to explore the statistically significant temporal changes in miRNA expression in hepatocarcinogenesis.

## Materials and Methods

### Sample preparation

We isolated total RNA from 14 surgically resected HCC tumors and 6 matched adjacent non-tumor control tissues (Table 1). We confirmed that the 14 samples were accurately diagnosed as HCC by image diagnosis by CT and USTG and pathological findings. All patients or their guardians provided written informed consent, and Osaka City University and Ogaki Municipal Hospital approved all aspects of this study in accordance with the Helsinki Declaration.

**Table 1:** Clinical information of 14 HCC patients analyzed in this study.

code No.	histological differentiation	sex	age	HBsAg	anti-HCV	AFP	PIVKA-II	no tumorous tissue	NGS analysis	microarray analysis
CU-070	moderately	M	53	(−)	(+)	3510	NI	LC	only HCC	only HCC
CU-083	moderately	M	49	(+)	(−)	143830	0.9	LC	both	only HCC
CU-085	moderately	M	55	(−)	(+)	113	0.06	LC	only HCC	only HCC
CU-087	well	M	62	(−)	(+)	NI	NI	NI	both	only HCC
CU-089	moderately	F	61	(−)	(+)	400	0.06	CH	both	only HCC
CU-091	moderately	M	64	(−)	(+)	5	0.06	CH	only HCC	only HCC
K-023	moderately	M	47	(+)	(−)	3500	2.2	LC	only HCC	only HCC
K-147	poorly	M	65	(−)	(+)	NI	NI	NI	only HCC	neither
K-175	moderately	M	67	(−)	(+)	3.4	32	CH	both	neither
K-177	moderately	M	68	(−)	(+)	13	29	NI	both	only HCC
K-181	well	M	LC	(−)	(−)	5.1	19	NI	both	neither
O-086	moderately	F	64	(−)	(+)	218	32	LC	only HCC	only HCC
O-088	moderately	M	74	(+)	(−)	13.4	7991	LC	only HCC	only HCC
O-089	moderately	M	68	(−)	(+)	8	25	LC	only HCC	only HCC

Abbreviations, moderately; moderately differentiated HCC, well; well differentiated HCC, poorly; poorly differentiated HCC, NI; no information, CH; chronic hepatitis, LC; liver cirrhosis, only HCC; only HCC was analyzed by NGS, both; both tumor and non-tumorous tissue were analyzed by NGS, neither; neither HCC nor non-tumorous tissue was not analyzed.

### RNA preparation, and miRNA deep sequencing and microarray

Total RNA from surgical resection was prepared using mirVana miRNA Isolation kit (Invitrogen), according to the manufacturer's instruction.

Total RNA, containing the small RNA fraction, was reverse transcribed into a cDNA library using the TruSeq Small RNA Sample Prep Kit (Illumina). Briefly, total RNA (1 µg per sample) was ligated overnight with adapters, reverse transcribed, RNase-treated, and PCR-amplified with unique barcode-labeled amplification primers. Then, size-selection was conducted on 6% native polyacrylamide gels. cDNA fragments between 145 and 160 bp corresponding to the miRNA populations were excised from the gel, then eluted and precipitated. The final cDNA pellet was air dried and resuspended in 10 µl of nuclease-free water. The quantity of cDNA in each final miRNA libraries was verified using Qubit fluorometer (Invitrogen). Equimoloar amounts for each final library were pooled at a final concentration of 2 nM cDNA. Barcoded templates were sequenced on a single flowcell of the Illumina MiSeq with 50-bp single-end reads. Eleven of 14 total HCC RNA samples were also assayed by microarray (Agilent human microRNA microarray release 14.0) (Table 1). Hybridization signals were detected with a DNA microarray scanner G2505B (Agilent Technologies) and the scanned images were analyzed using Agilent feature extraction software (v9.5.3.1). Raw data (gProcessedSignal) was normalized so that each expression had a mean of zero and a sample variance of one. The above processes were conducted with various packages and functions implemented in R {http://www.r-project.org}.

The sequence reads obtained in this study have been deposited in the DNA Data Bank of Japan Sequence Read Archive (http://www.ddbj.nig.ac.jp/index-e.html) under accession number DRA001067. All microarray data were deposited in NCBI's Gene Expression Omnibus and are accessible through GEO Series accession number GSE31164.

### Bioinformatics

In order to extract the adaptor sequence from each short read obtained by NGS, fastx_clipper from the fastx toolkit {http://hannonlab.cshl.edu/fastx_toolkit/} was used. The adaptor trimmed short reads were then mapped to the human reference genome sequence hg19 by mapper.pl script included in miRDeep2 [19] {http://genomewiki.ucsc.edu/index.php/Hg19_Genome_size_statistics}. miRNA mature and hairpin sequences were obtained from miRBase release 18 {http://www.mirbase.org}. Finally, the resulting fastq files were processed by miRDeep2.pl script. miRNA read counts were extracted from the “read_count” column of the file named“miRNAs_expressed_all_samples_sample_id.csv” file; while sample_id was given automatically by scripts (for sample scripts see Text S1 and for extracted read counts for each miRNA see Table S1). When drawing boxplots, the read count in each sample was normalized such that it had a zero mean and a variance of one. For microarray processing, gProcessedSignal values were extracted from raw data files. gProcessedSignal values were also normalized so that they had a zero mean and a variance of one. Average gProcessedSignal values over probes assigned to common mature miRNA were used to compute the correlation coefficient for NGS and microarray results. The correlation coefficients of logarithmic expression and subtracted expression were computed using only miRNAs with non-negative signals in both NGS and average microarray expression. P-values associated with boxplots were computed using Wilcoxon rank sum test. All bioinformatics computation was performed using functions implemented in R.

To discriminate HCC from non-tumorous tissue when miRNA expression was quantified by NGS, we combined principal components analysis (PCA)-based feature extraction and PCA-based linear discriminant analysis (LDA) [20] [21]. When PCA-based feature extraction was applied, each miRNA was embedded into a two-dimensional space by PCA and M miRNAs located far from the origin (outliers) were selected. Using these selected miRNA, each sample was embedded into a low dimensional space with dimension (M–M'). Samples were then discriminated by LDA using the M' dimensional PC scores. For more details, see Text 2. Novel miRNA candidates were selected from the total set if they satisfied the following criteria: 1) among the novel miRNAs identified by miRDeep2, those with a >80% probability of being a true positive, and 2) the miRNA was reproducibly detected in more than three samples.

## Results

### Analysis of miRNA sequence reads and reproducibility of NGS analysis

**Figure 1:**
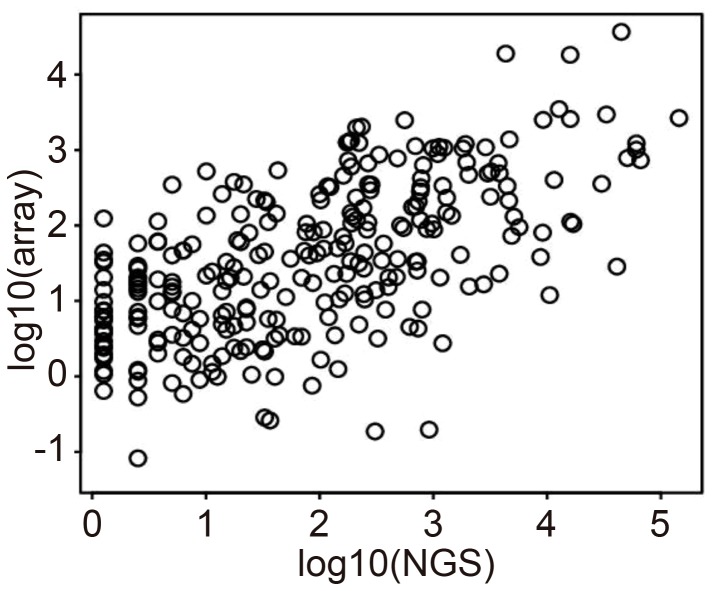
Comparison between logarithmic HCC miRNA expression in NGS (horizontal axis) and microarray (vertical) analysis (K-177_1 means the first technical replicates of code No. K-177). One black circle showed one miRNA. Pearson's correlation coefficient is 0.6059.

The average number of sequencing reads per sample exceeded 2.2 million, of which approximately 57% mapped to the human reference genome (for more details, see Table S2). A scatter plot of logarithmic miRNA expression measured by NGS and microarray using the first technical replicate of K-177 (K-177_1) is shown in Fig 1.

Although the microarray and NGS expression levels are not perfectly correlated, they are approximately proportional to each other with a positive proportional coefficient. The corresponding scatter plots for the remaining 9 HCC samples are shown in Fig. S1. The similarity in the NGS and microarray miRNA profiling results was relatively independent of the samples considered. The correlation coefficient of logarithmic miRNA expression from 11 HCC miRNA expression profiles as measured by both NGS and microarray was 0.613. This demonstrates that NGS and microarray measurements give similar results.

**Figure 2:**
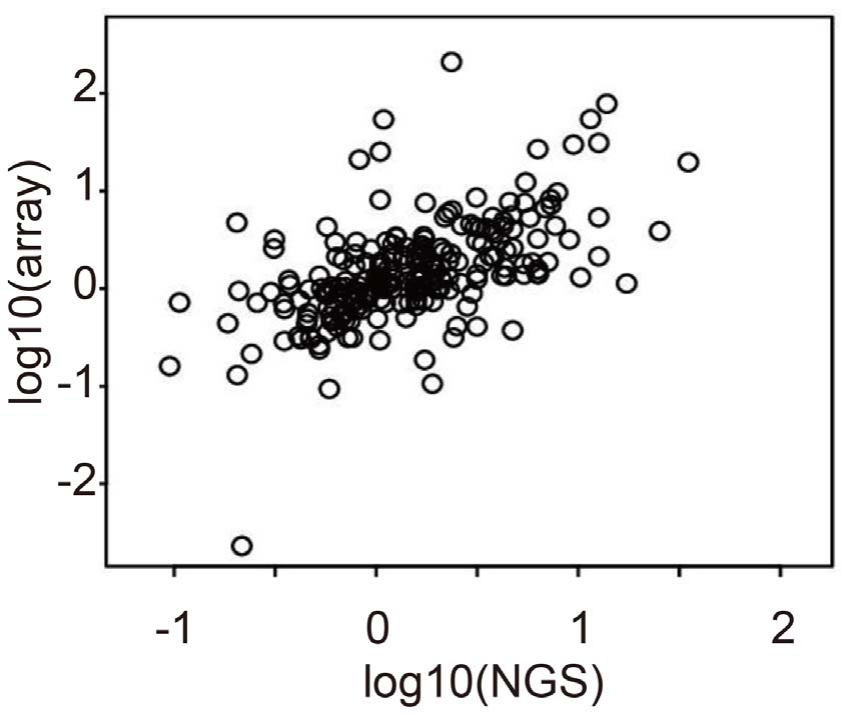
Comparison between differential (K-177 vs. CU-087) logarithmic HCC miRNA expression in NGS (horizontal axis) and microarray (vertical) analysis. Pearson's correlation coefficient is 0.5555.

We next validated the reproducibility of miRNA differential expression across the 11 samples. In general, miRNA expression profiles are not individually evaluated; instead, profiles for distinct samples are analyzed in pairs e.g., compared between tumor and adjacent non-tumorous tissue. Thus, reproducibility between NGS and microarray is more important in differential expression than in any single expression measurement by itself. Fig. 2 shows a scatter plot of differential (K-177 vs. CU-087) logarithmic miRNA expression obtained by NGS and microarray (Fig. S2 shows the full set of scatter plots). Again, we observed that the coincidence between NGS and microarray miRNA profiles was relatively strong irrespective of the sample pair that was considered. The correlation coefficient of differential logarithmic miRNA expression averaged over all pairs of 11 HCC is 0.587. This demonstrates a reasonable level of congruency between miRNA profiling results from NGS and microarray when considering differential logarithmic miRNA expression.

### miRNA expression measured by NGS can be applied to diagnosing HCC

**Figure 3:**
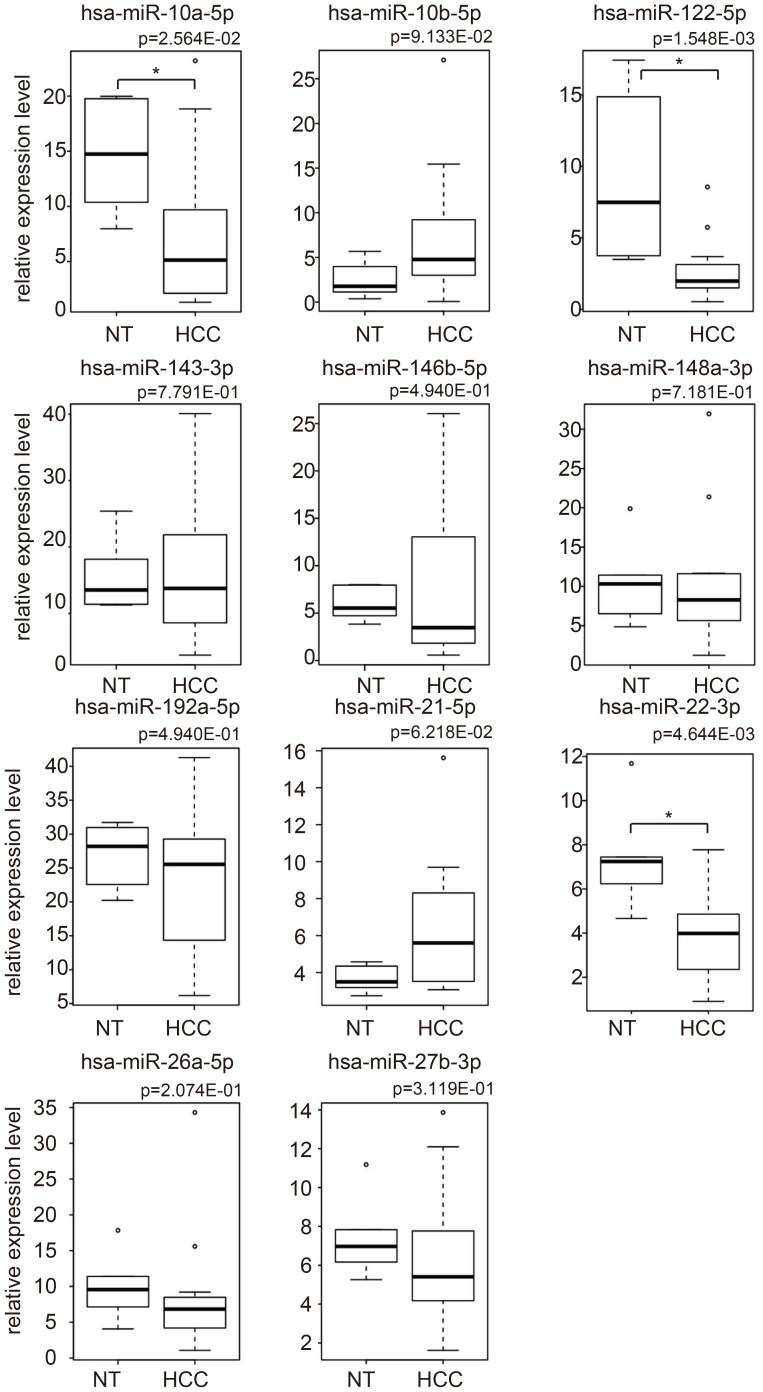
Boxplots of the expression of 11 miRNAs in HCC and non-tumorous tissue obtained by NGS, which were used for the differential analysis. P-values were computed using two-sided Wilcoxon Rank Sum test. Asterisk indicates a significant difference of p<0.05 (*).

In order to discriminate between HCC and non-tumorous tissue, 11 miRNAs quantified by NGS (miR-10a-5p, miR-122-5p, miR-146b-5p, miR-148a-3p, miR-192-5p, miR-22-3p, miR-26a-5p, and miR-27b-3p, miR-10b-5p, miR-143-3p, and miR-21-5p) were chosen by PCA-based feature extraction. The miRNA expression levels in HCC and non-tumorous tissue is shown in Fig 3. The expression level of miR-10a-5p (p<2.56×10^−2^), miR-122-5p (p < 1.55×10^−3^), and miR-22-3 (p<4.64×10^−3^) among the 11 miRNAs differed significantly in the HCC and non-tumorous tissue. miRNA profiling allowed the accurate prediction of HCC with an overall cross-validation accuracy of 90.0% (18/20) by PCA-based feature extraction (Table 2). The p-value and AUC value for diagnostic ability were <7.22×10^−4^ and 0.92 respectively.

**Table 2:** Performance of discrimination between 14 HCC samples and 6 normal tissue samples using miRNA expression obtained by NGS analysis.

		Result	
		Control	Tumor
Prediction	Control	12	0
	Tumor	2	6

### Reproducibility of NGS measurement among technical replicates

**Figure 4:**
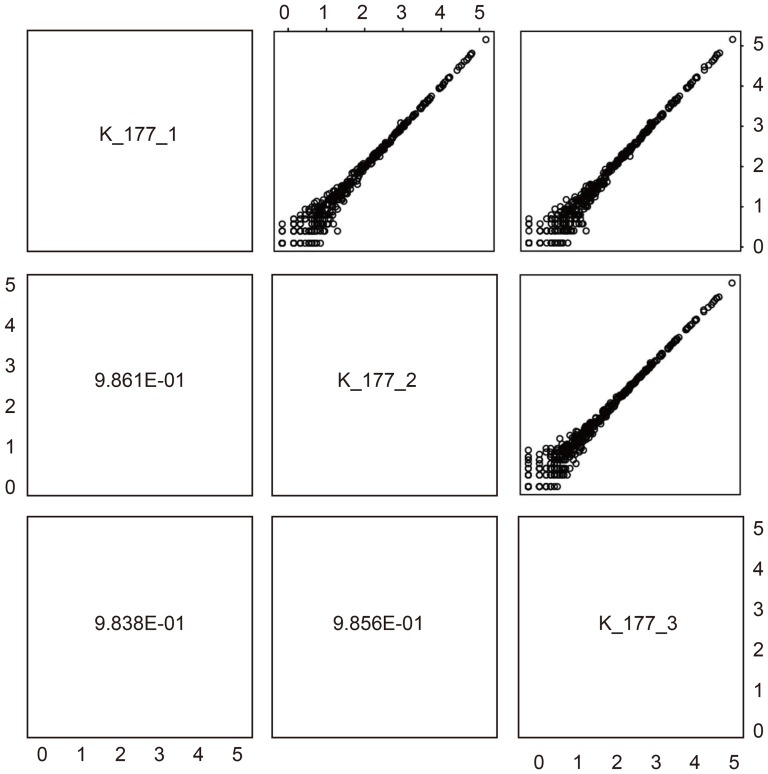
Comparison between logarithmic miRNA expression in HCC and NGS technical replicates (K-177_1 means the first technical replicates of code No. K-177). Pearson's correlation coefficients are greater than 0.9861.

We have demonstrated that quantifying miRNA using NGS gives results similar to those obtained by microarray, and that miRNA expression measured by NGS can discriminate HCC from non-tumorous tissue. However, we have found that miRNA profiling using NGS is more accurate in cases where NGS measurement does not vary within multiple technical replicates. For those HCC samples in this study that had more than one technical replicate we validated the reproducibility of NGS miRNA expression between technical replicates. Fig. 4 shows examples of technical replicates (other scatter plots are available in Fig. S3). Among three technical replicates, the correlation coefficients of logarithmic miRNA expression are greater than 0.98. Additionally, the dynamic range is almost 5 digits. This means that technical replicates obtained from NGS measurements are highly reproducible.

### Discovery of novel miRNAs in our analysis

**Figure 5:**
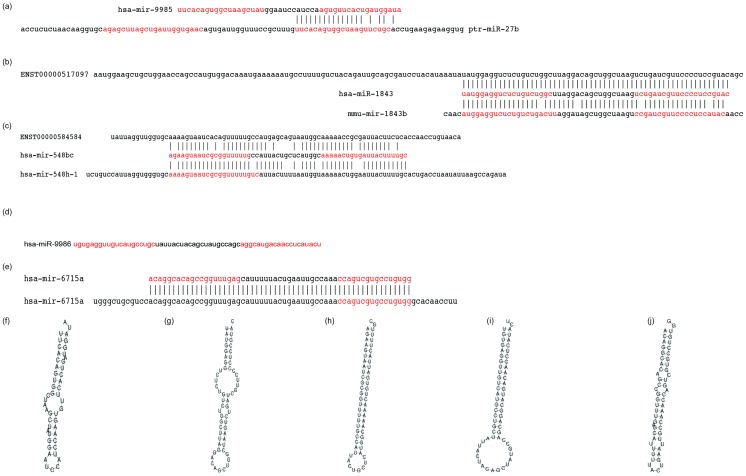
Comparison of novel miRNA candidates ((a) hsa-mir-9985, (b) hsa-mir-1843, (c) hsa-mir-548bc and (d) hsa-mir-9986) with known miRNAs and transcripts. (e) hsa-mir-6715a novel miRNA was not included in miRBase release 18, but was present in later releases. Nucleotides highlighted in red show hairpin constructs. F, g, h, i, and j are constructs of each miRNA and respectively correspond to a, b, c, d, and e.

NGS detected several miRNA candidates that are not registered in the present miRBase (Rel. 18) (see Materials and Methods for detection criteria, and see Supporting Information for more details about detected miRNAs). We speculate that four precursor miRNAs, hsa-mir-9985, hsa-mir-1843, hsa-mir-548bc, and hsa-mir-9986 and the corresponding four mature miRNAs, hsa-miR-9985, hsa-miR-1843, hsa-miR-548bc, and hsa-miR-9986, were not previously reported because they are not among the miRNAs found at the corresponding genomic coordinates. Fig. 5 shows the sequence of the new miRNA candidates, their alignment with their closest homologous miRNA, and the hairpin structure predicted by RNA-fold with default parameter settings (short reads mapped to these candidate miRNAs are available in Table S3).

## Discussion

The clinical application of miRNA expression profiling, such as its use as a disease biomarker, has been extensively developed in recent years. This analysis demonstrates that miRNA profiling by NGS has the potential to diagnose HCC with high accuracy. Previous comprehensive analyses of miRNA expression have been performed by microarray; however, the miRBase is currently underdeveloped. An updated miRBase is required to create accurate microarray profiles, and especially because microarray experiments performed using previous version of miRBase are incompatible with microarrays based on the current version. The results from our miRNA expression analysis in HCC suggest that NGS may allow us to overcome this problem. Law et al. has previously reported small RNA transcriptome analysis in HCC by using NGS [18]. However, pre-miRNAs are of a similar length as other more numerous classes of ncRNA, including tRNA and snoRNA, making deep profiling of pre-miRNA sequences difficult [22]. Therefore, this study focused on the analysis on whole miRNA instead of ncRNA.

In order to determine if miRNA expression as measured by NGS technology can discriminate HCC from non-tumorous tissue, we adopted a recently proposed combination of PCA-based feature extraction and PCA-based LDA [20] [21] (for details, see Materials and Methods). Previously, we showed that miRNA expression profiles detected by microarray can accurately discriminate HCC from non-tumorous tissues [7] [23] [24] [25]. Therefore, because NGS produced results similar to those by microarray, and was capable of differentiating HCC from non-tumorous tissues, it is evident that miRNA expression quantified by NGS is as informative as is miRNA expression assessed by microarray. NGS's ability to quantify miRNA expression suggests that the miRNA expression profile measured by NGS can be clinically applicable as a diagnostic tool.

HCC was discriminated from non-tumorous tissues using a linear combination of 11 miRNA expression profiles; however, it may be useful to investigate the expression of individual miRNA in order to understand their biological significance. Fig. 3 shows boxplots of the 11 miRNAs that were used to discriminate between HCC and non-tumorous tissue. Contrary to our expectations, only three (miR-10a-5p, 122-5p and 22-3p) of the 11 miRNAs showed significant differential expression (p<0.05) between HCC and non-tumorous tissue. Among these three miRNAs, miR-122-5p and miR-22-3p are well-known to be downregulated in HCC [10] [10] [26] [27]. Among the three miRNAs with significant differential expression between HCC and non-tumorous tissue, miR-10a-5p showed the least difference, with a p-value of 0.03. Recently, it has been reported that miR-10a-5p is involved in HCC metastasis [28]. Thus, the selection of these three miRNAs as potential biomarkers is biologically reasonable. Although the remaining eight miRNAs did not have significant differential expression between HCC and non-tumorous tissues, their inclusion did not reduce the discriminatory performance. This suggests that these eight miRNAs may contribute to diagnosing hepatocarcinogenesis only when combined with other miRNAs. Since controlling the expression of multiple miRNAs simultaneously is experimentally difficult, we were unable to confirm if these miRNAs work together. This may be worth further study in the future.

While investigating novel miRNA candidates, we found that mature miRNAs from hsa-mir-9985 and hsa-miR-9985-5p are almost identical to ptr-miR-27a-3p (Fig 5(a, f)). This may suggest that hsa-mir-9985 has been transposed during its evolution from chimpanzee to human. hsa-miR-1843 was homologous to miR-1843a, which was previously detected in mouse, Chinese hamsters, and brown rats. Near the genomic coordinate where hsa-mir-1843 was detected, Ender reported finding snoRNA (ENSR00000517097), therefore hsa-mir-1843 is likely to be a small nuclear RNA-derived microRNA [29] (Fig, 5(b, g)). It is possible that this explains why researchers have overlooked hsa-mir-1843 despite reports of homologous miRNAs in other animals. While hsa-mir-548bc appears homologous to hsa-mir-548h-1 (Fig. 5(c, h)), hsa-mir-548bc is detected at a distinct genomic coordinate from the original location of hsa-548h-1. Thus, hsa-miR-548bc seems to be a distinct microRNA from hsa-mir-548h-1.

It is interesting to note that the novel miRNA (EST00000584584) not included in the miRBase was reported to be located near the genomic coordinate where hsa-mir-9986 was detected. To our knowledge, this is the first experimental evidence that these predicted novel miRNAs exist. Finally, no homologous miRNA has been reported for hsa-mir-9986 (Fig. 5(d, i)), thus it is possible that hsa-mir-9986 is completely new. In addition to these four new miRNA candidates, hsa-mir-6715a was also detected in our analysis (Fig. 5(e, j)). hsa-miR-6715a was not included in the miRBase release 18, but was included in later releases, which lends support to the reliability and accuracy of our strategy to identify novel miRNAs. It is interesting that although hsa-miR-6715a-5p was not reported in the miRBase, it was detected in our analysis although only 10 reads were assigned to the miRNA. The biological significance of these candidate miRNAs requires future examination.

**Figure 6:**
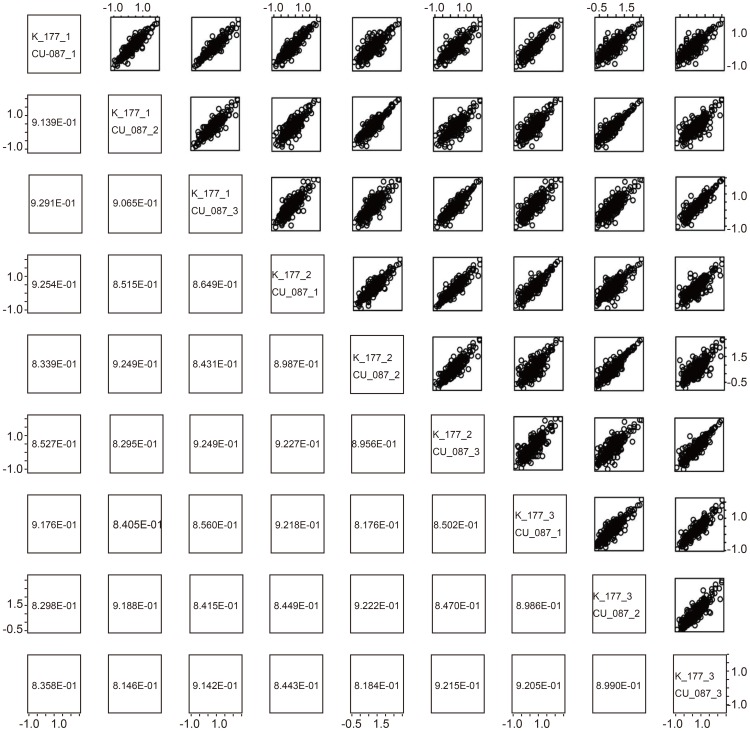
Comparison of differential (K-177 vs. CU-087) logarithmic miRNA expression in HCC among NGS technical replicates. Pearson's correlation coefficients range from 0.80 to 0.93.

Finally, we also investigated the reproducibility of technical replicates for differential miRNA expression; a full list of scatter plots and correlation coefficients is available in Fig. S4. Fig. 6 shows examples of technical replicates with good correlation. We were unable to identify the specific conditions necessary to achieve highly reproducible technical replicates. However, several of our cross-replicate comparisons were of acceptable quality, leading us to believe that our method has the potential to adequately reproduce differential miRNA expression between technical replicates.

## Conclusions

We have shown in this study that miRNA expression profiles obtained from NGS analysis are reproducible and are concordant with that obtained by the standard microarray procedure. Moreover, we have demonstrated that NGS can identify novel miRNAs that are otherwise undetectable by microarray analysis. HCC was distinguished from non-tumorous tissue with high diagnostic accuracy, supporting the clinical application of NGS-based miRNA expression profiling.

## Supporting Information

Figure S1 Full set of scatter plots of logarithmic miRNA expression in HCC samples by NGS and microarray analysis. Comparison between logarithmic HCC miRNA expression in NGS (horizontal axis) and microarray (vertical) analysis. One black circle showed one miRNA.
(PDF)

Figure S2 Full set of scatter plots of differential logarithmic miRNA expression in HCC for NGS and microarray analysis. Comparison between differential logarithmic HCC miRNA expression in NGS (horizontal axis) and microarray (vertical) analysis. One black circle showed one miRNA.
(PDF)

Figure S3 Comparison of logarithmic miRNA expression in HCC for NGS technical replicates not included in Fig. 3. Comparison between differential logarithmic HCC miRNA expression in NGS (horizontal axis) and microarray (vertical) analysis. One black circle showed one miRNA.
(PDF)

Figure S4 Comparison of differential logarithmic miRNA expression in HCC for NGS technical replicates not included in Fig. 4. Comparison between differential logarithmic HCC miRNA expression in NGS (horizontal axis) and microarray (vertical) analysis. One black circle showed one miRNA.
(PDF)

Table S1 Extracted read counts of each miRNA obtained by NGS analysis.
(PDF)

Table S2 Detailed NGS analysis of HCC and non-tumorous tissue samples.
(PDF)

Table S3 Detailed mapping of short reads for novel miRNA candidates and hsa-mir-6715a.
(PDF)

Text S1 Sample script that processed short reads using fastx_clipper and miRDeep2.
(PDF)

Text S2 Detailed description of feature extraction and discriminant procedures.
(PDF)
