# Correction: Comparison of Hepatocellular Carcinoma miRNA Expression Profiling as Evaluated by Next Generation Sequencing and Microarray

**DOI:** 10.1371/journal.pone.0116434

**Published:** 2014-12-19

**Authors:** The PLOS ONE Staff

In Table 2, the headings Tumor and Control are incorrectly swapped in the second column and in the second row. Please see the corrected Table 2 here.

**Table 2:** Performance of discrimination between 14 HCC samples and 6 normal tissue samples using miRNA expression obtained by NGS analysis.

		Result	
		Tumor	Control
Prediction	Tumor	12	0
	Control	2	6
