# Supplementary material for: Comparison of Hepatocellular Carcinoma miRNA Expression Profiling as Evaluated by Next Generation Sequencing and Microarray
Source: PLoS One. 2014 Sep 12;9(9):e106314. doi: 10.1371/journal.pone.0106314 (PMC4162537; doi:10.1371/journal.pone.0106314)
Supplement: Figure S1 — Full set of scatter plots of logarithmic miRNA expression in HCC samples by NGS and microarray analysis. Comparison between logarithmic HCC miRNA expression in NGS (horizontal axis) and microarray (vertical) analysis. One black circle showed one miRNA. (PDF) [file pone.0106314.s001.pdf]

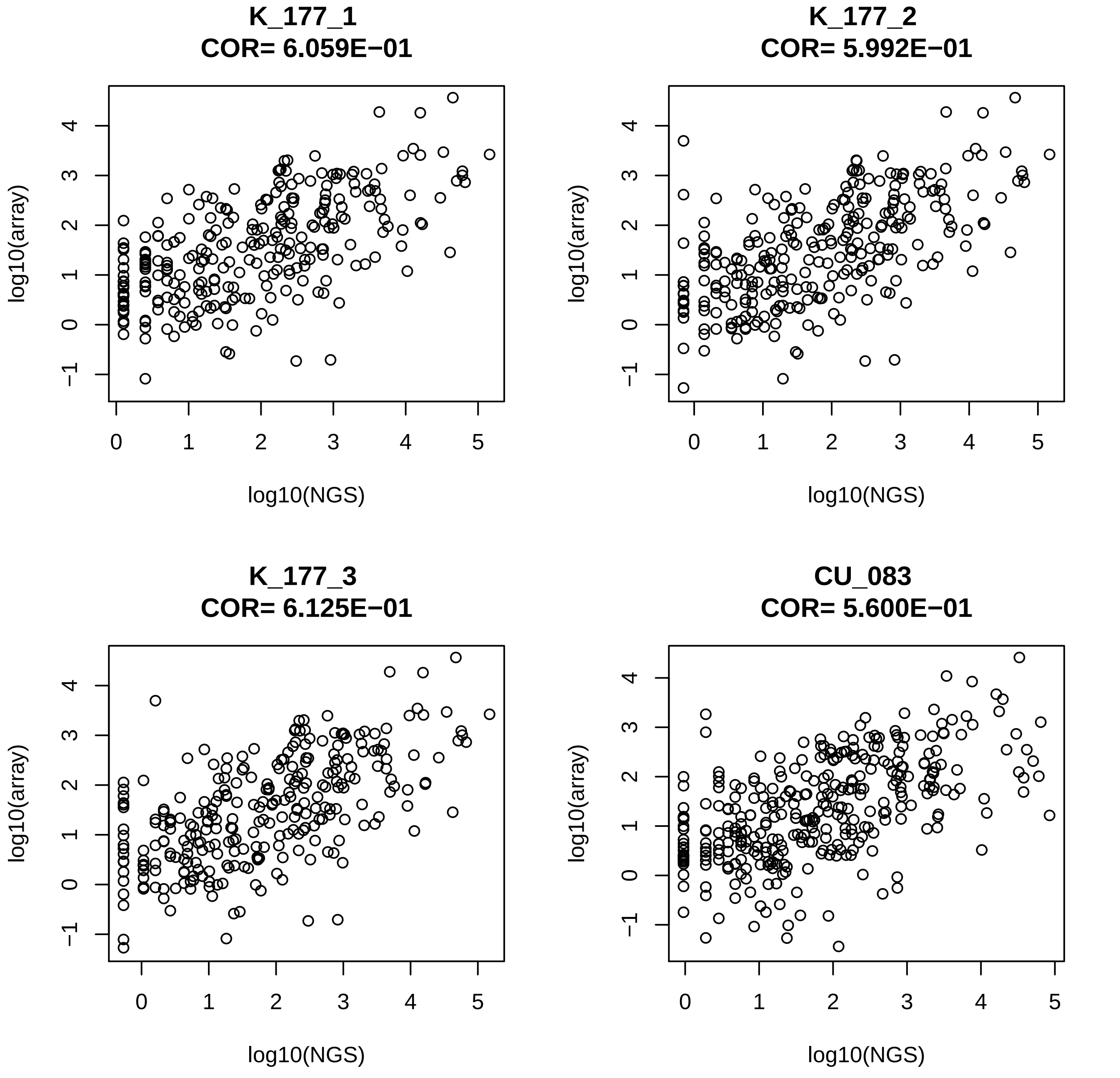
K_177_1
COR= 6.059E−01
K_177_2
COR= 5.992E−01
K_177_3
COR= 6.125E−01
CU_083
COR= 5.600E−01
log10(array)
log10(NGS)
−1
0
1
2
3
4
5

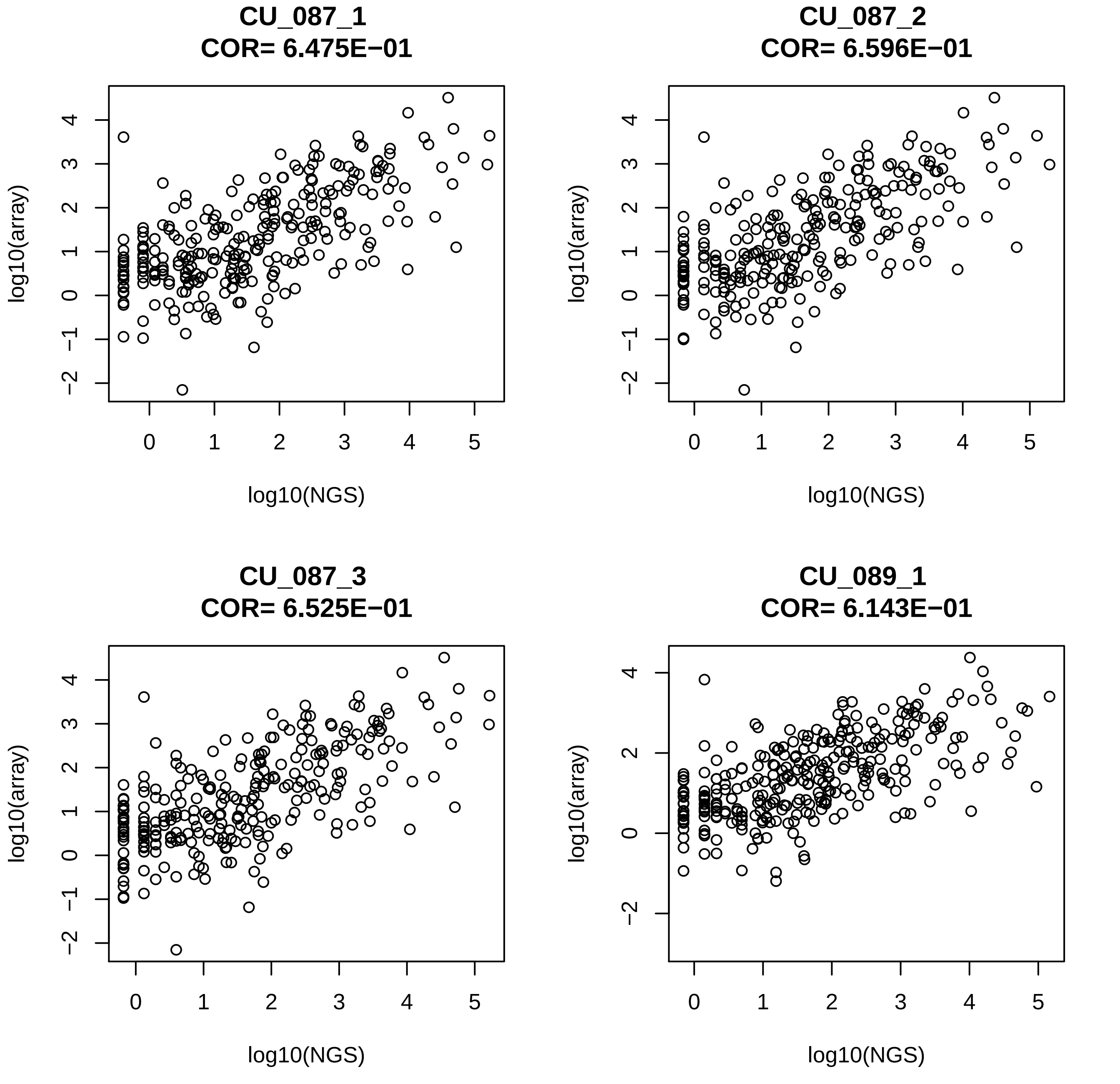
CU_087_1
COR= 6.475E−01
log10(array)
log10(NGS)
CU_087_2
COR= 6.596E−01
log10(array)
log10(NGS)
CU_087_3
COR= 6.525E−01
log10(array)
log10(NGS)
CU_089_1
COR= 6.143E−01
log10(array)
log10(NGS)

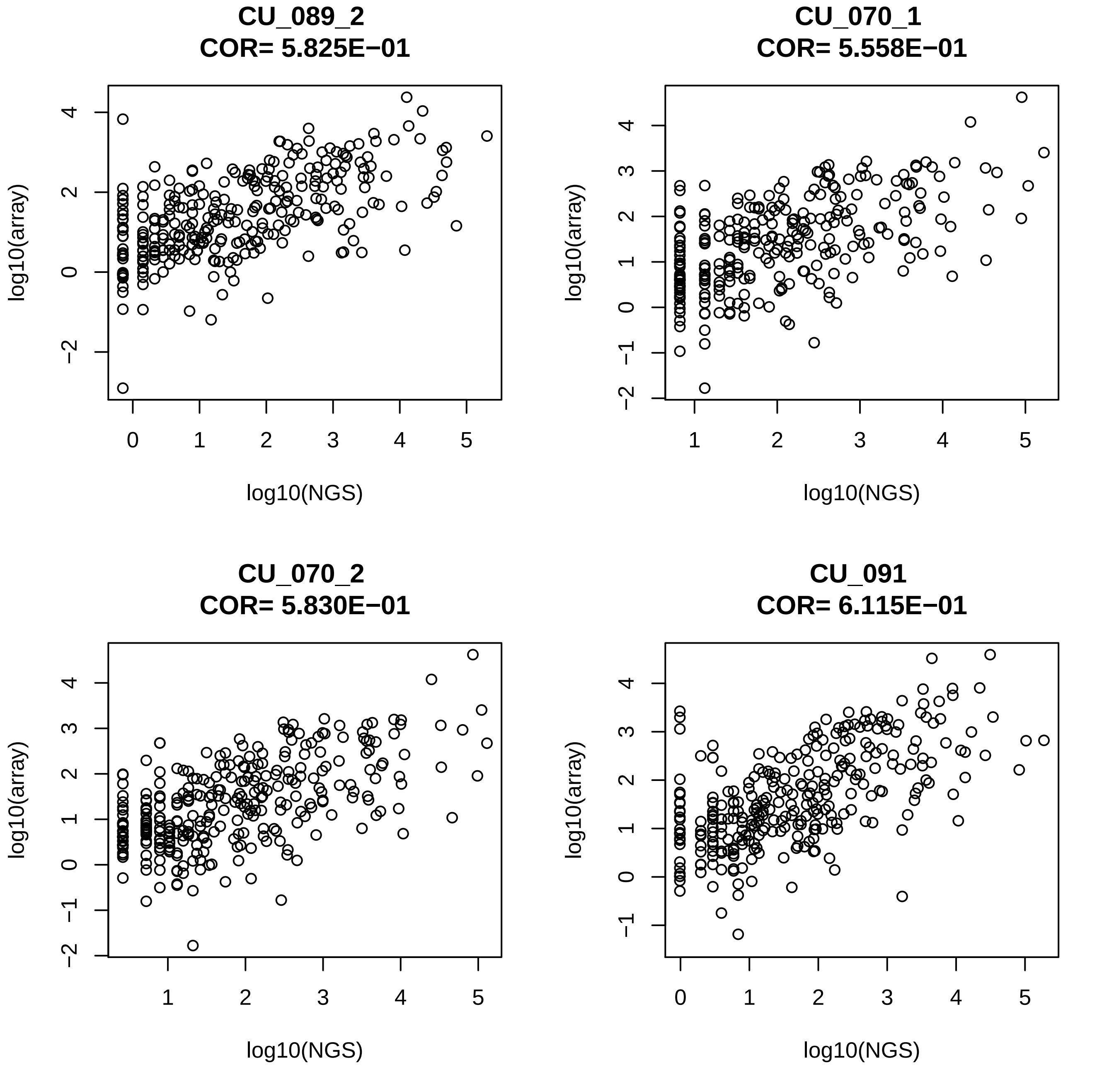
CU_089_2
COR= 5.825E−01
log10(array)
log10(NGS)
CU_070_1
COR= 5.558E−01
log10(array)
log10(NGS)
CU_070_2
COR= 5.830E−01
log10(array)
log10(NGS)
CU_091
COR= 6.115E−01
log10(array)
log10(NGS)

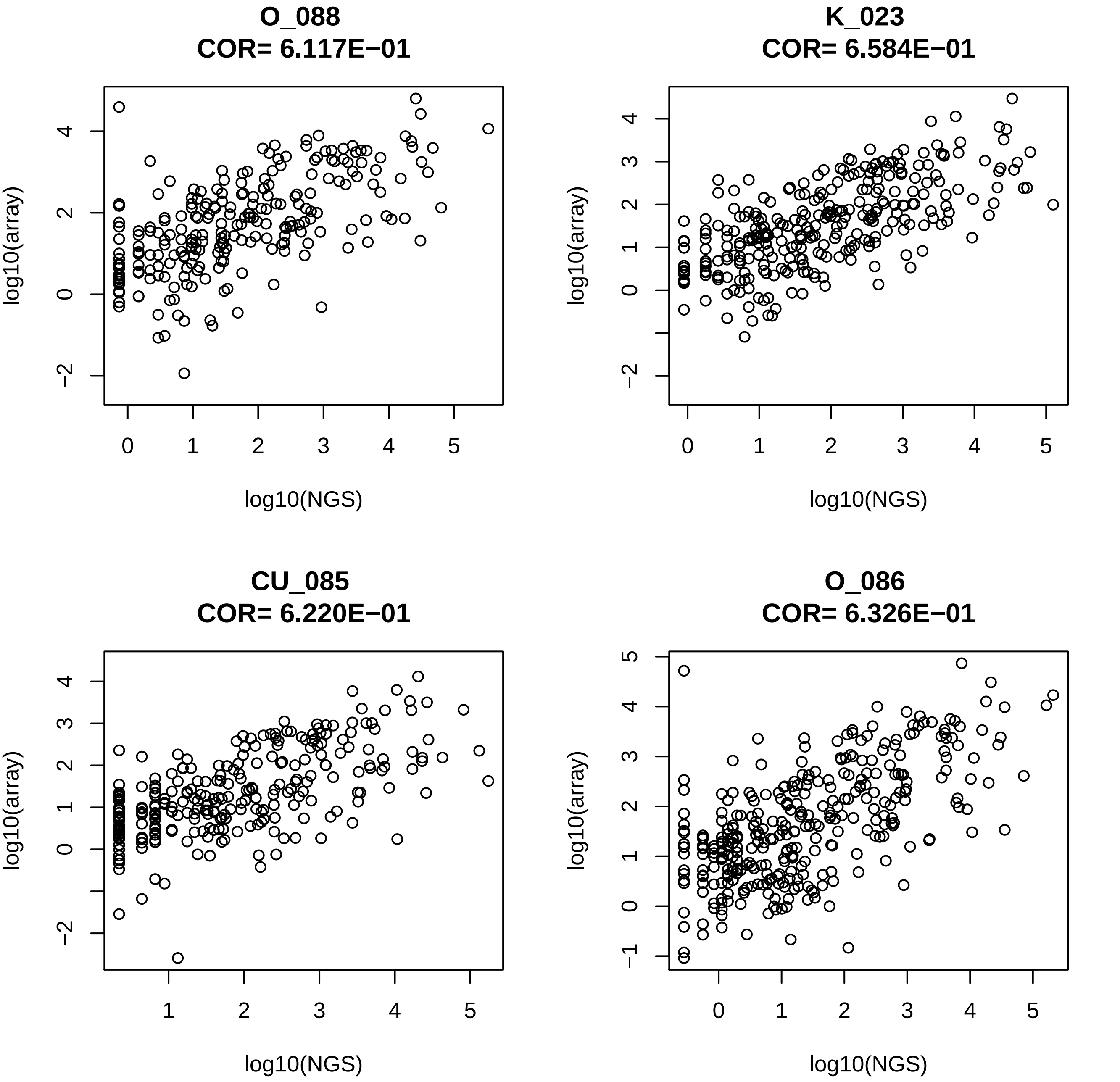
O_088
COR= 6.117E−01
K_023
COR= 6.584E−01
CU_085
COR= 6.220E−01
O_086
COR= 6.326E−01
log10(array)
log10(NGS)
