# Supplementary material for: Comparison of Hepatocellular Carcinoma miRNA Expression Profiling as Evaluated by Next Generation Sequencing and Microarray
Source: PLoS One. 2014 Sep 12;9(9):e106314. doi: 10.1371/journal.pone.0106314 (PMC4162537; doi:10.1371/journal.pone.0106314)
Supplement: Figure S2 — Full set of scatter plots of differential logarithmic miRNA expression in HCC for NGS and microarray analysis. Comparison between differential logarithmic HCC miRNA expression in NGS (horizontal axis) and microarray (vertical) analysis. One black circle showed one miRNA. (PDF) [file pone.0106314.s002.pdf]

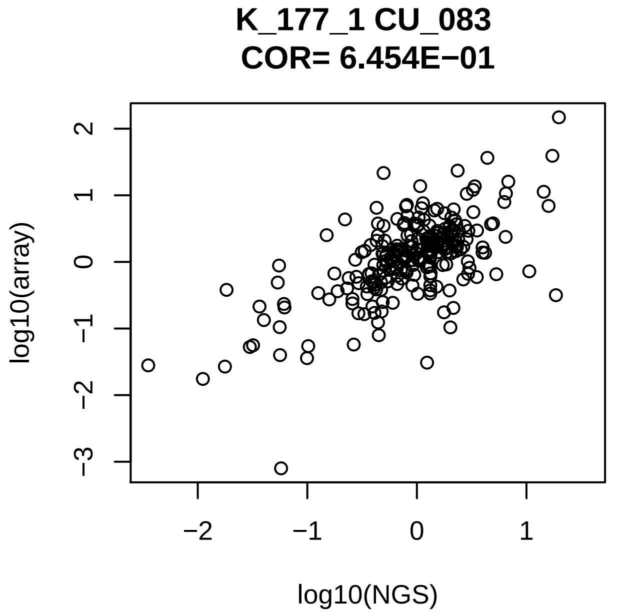
K_177_1 CU_083
COR= 6.454E−01
log10(array)
log10(NGS)
−2
−1
0
1
2
−3

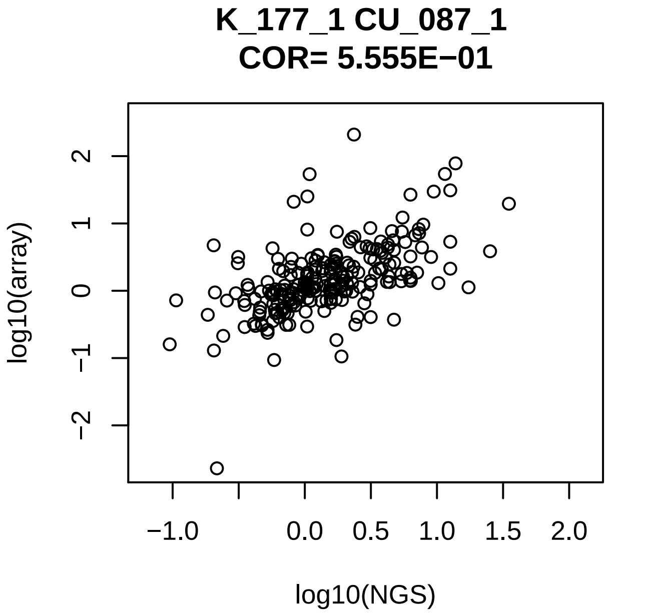
K_177_1 CU_087_1
COR= 5.555E−01
log10(array)
log10(NGS)
−1.0
0.0
0.5
1.0
1.5
2.0
−2
−1
0
1
2

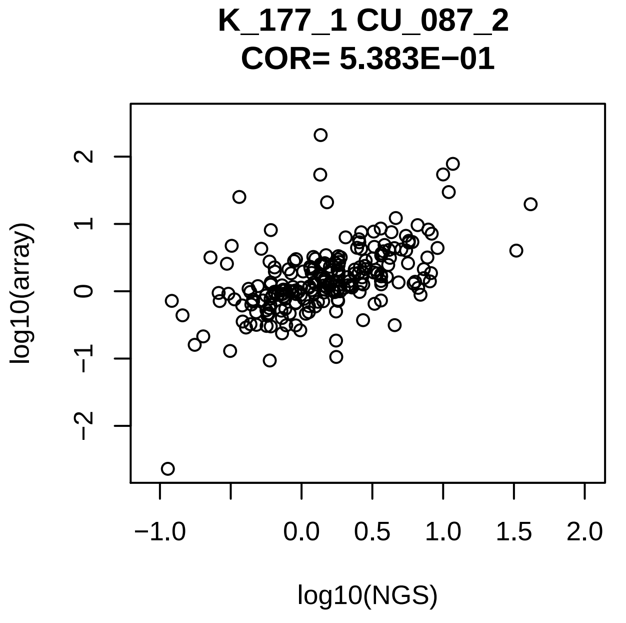
K_177_1 CU_087_2
COR= 5.383E−01
log10(array)
log10(NGS)
−1.0
0.0
0.5
1.0
1.5
2.0
−2
−1
0
1
2

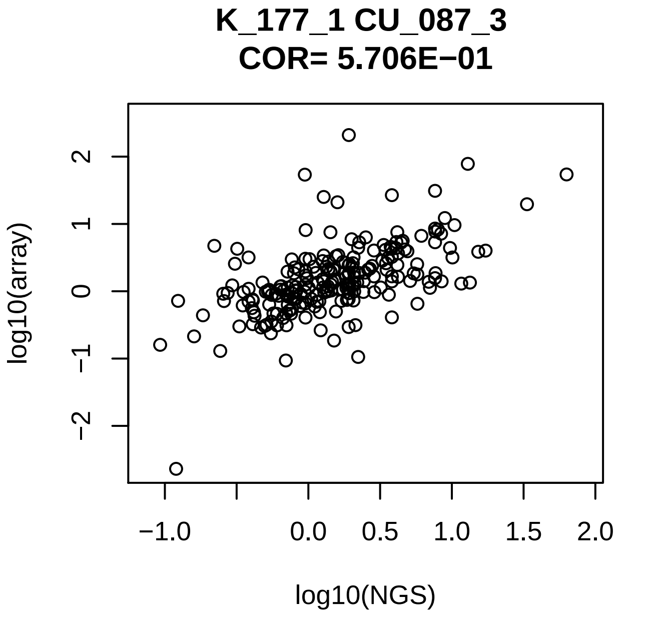
K_177_1 CU_087_3
COR= 5.706E−01
log10(array)
log10(NGS)
−1.0
0.0
0.5
1.0
1.5
2.0
−2
−1
0
1
2

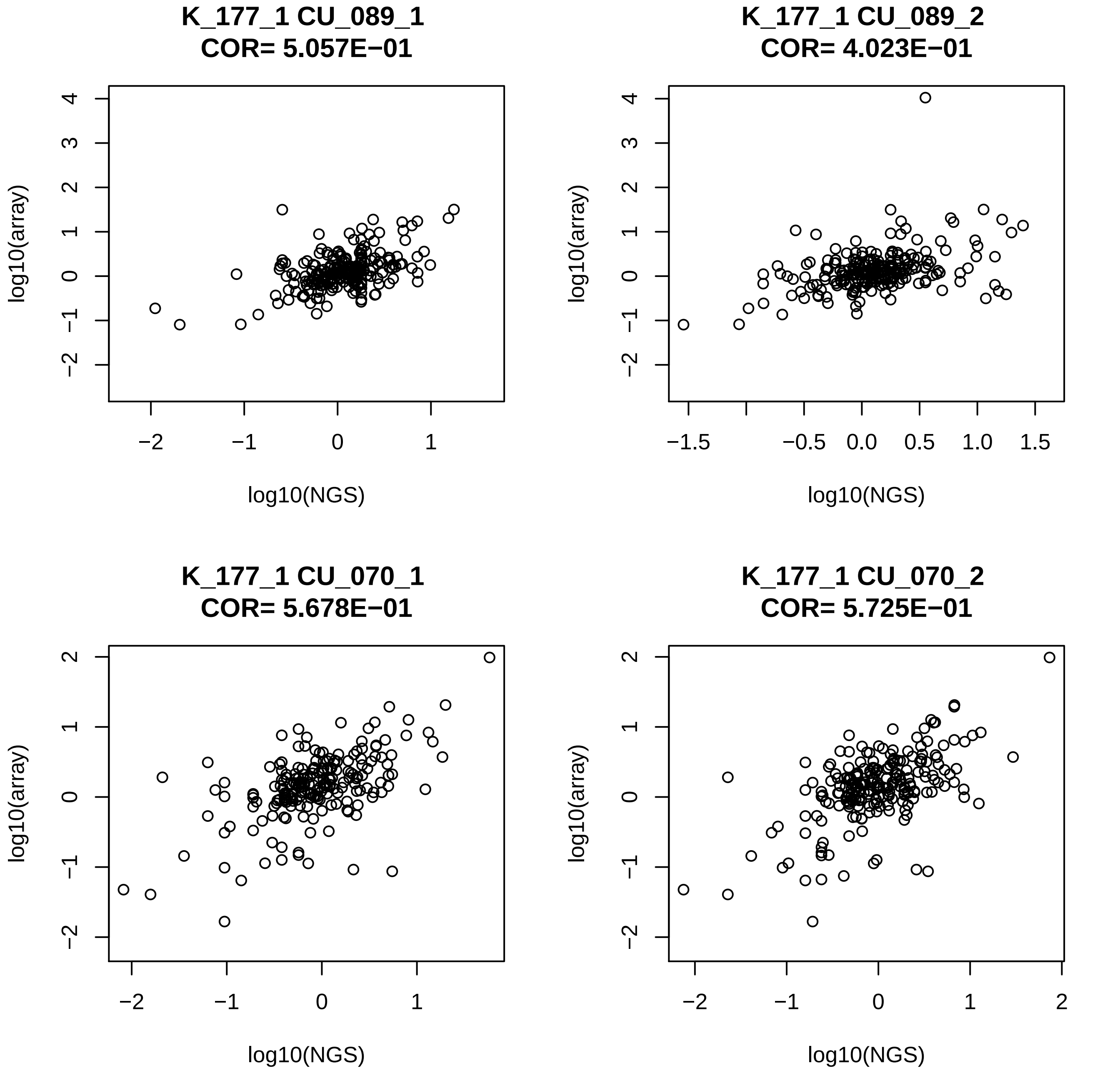
K_177_1 CU_089_1
COR= 5.057E−01
log10(array)
log10(NGS)
K_177_1 CU_089_2
COR= 4.023E−01
log10(array)
log10(NGS)
K_177_1 CU_070_1
COR= 5.678E−01
log10(array)
log10(NGS)
K_177_1 CU_070_2
COR= 5.725E−01
log10(array)
log10(NGS)

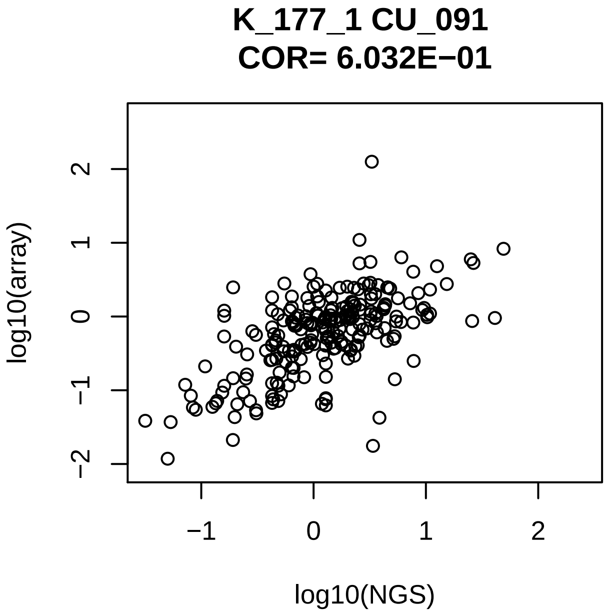
K_177_1 CU_091
COR= 6.032E−01
log10(array)
log10(NGS)

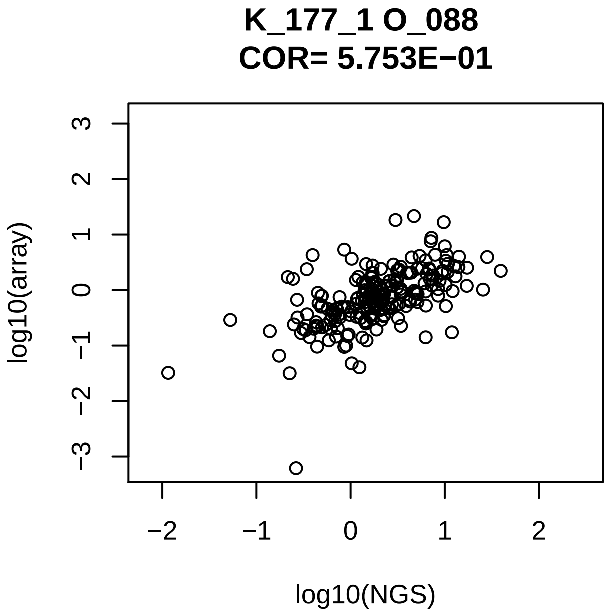
K_177_1 O_088
COR= 5.753E−01
log10(array)
log10(NGS)

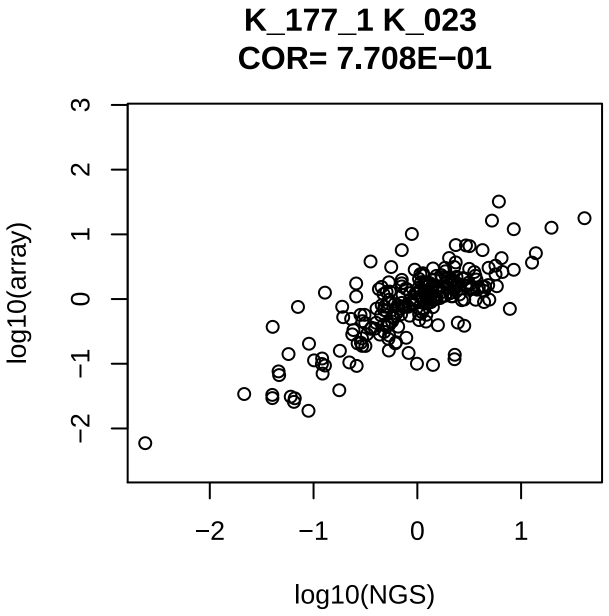
K_177_1 K_023
COR= 7.708E−01
log10(array)
log10(NGS)

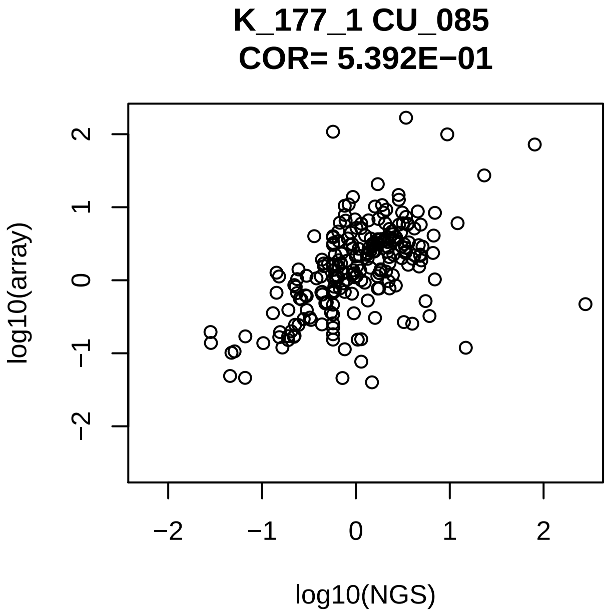
K_177_1 CU_085
COR= 5.392E−01
log10(array)
log10(NGS)

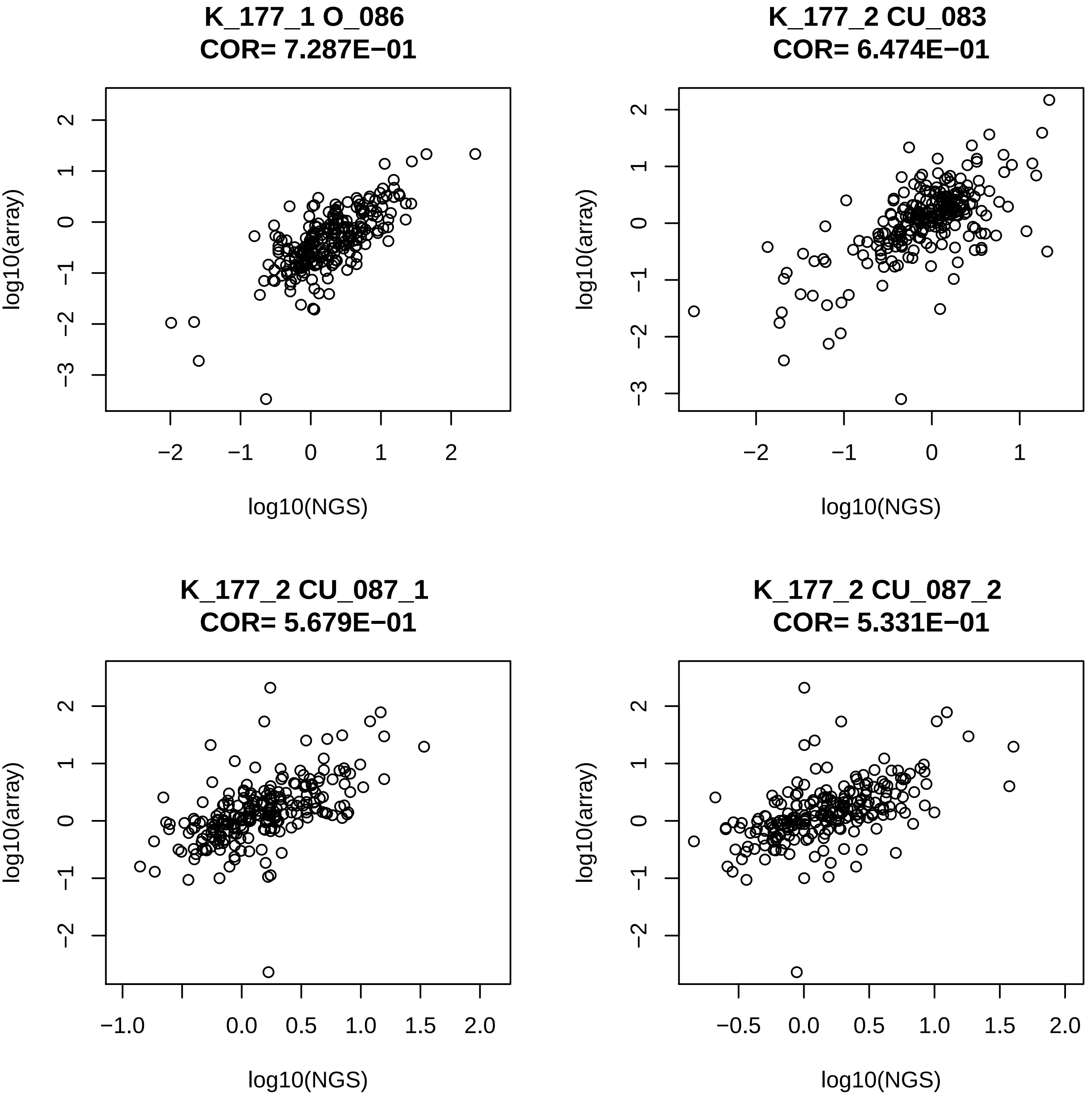
K_177_1 O_086
COR= 7.287E−01
log10(array)
log10(NGS)
K_177_2 CU_083
COR= 6.474E−01
log10(array)
log10(NGS)
K_177_2 CU_087_1
COR= 5.679E−01
log10(array)
log10(NGS)
K_177_2 CU_087_2
COR= 5.331E−01
log10(array)
log10(NGS)

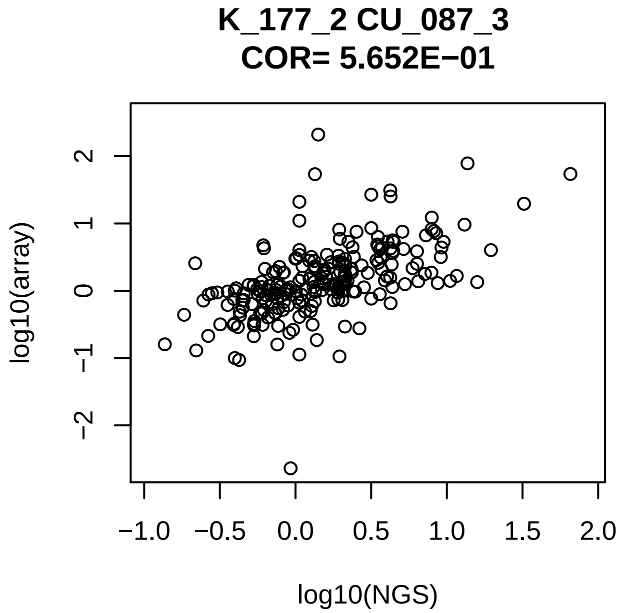
K_177_2 CU_087_3
COR= 5.652E−01
log10(array)
log10(NGS)
−2
−1
0
1
2
−1.0
−0.5
0.0
0.5
1.0
1.5
2.0

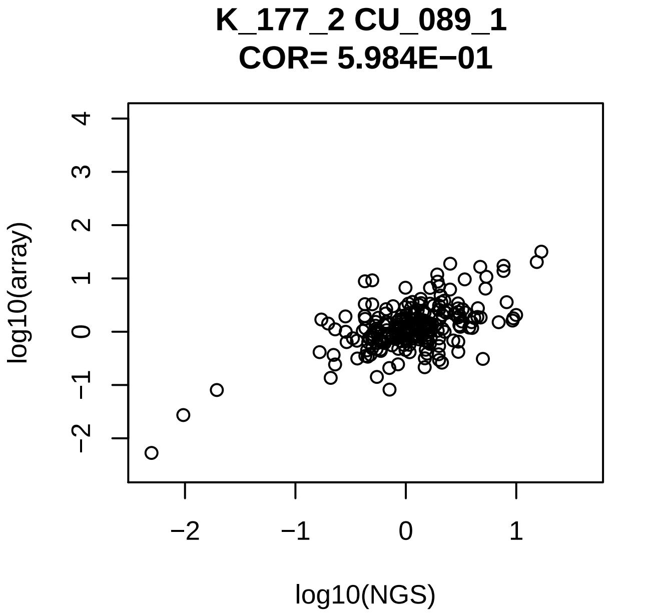
K_177_2 CU_089_1
COR= 5.984E−01
log10(array)
log10(NGS)
−2
−1
0
1
2
3
4
−2
−1
0
1

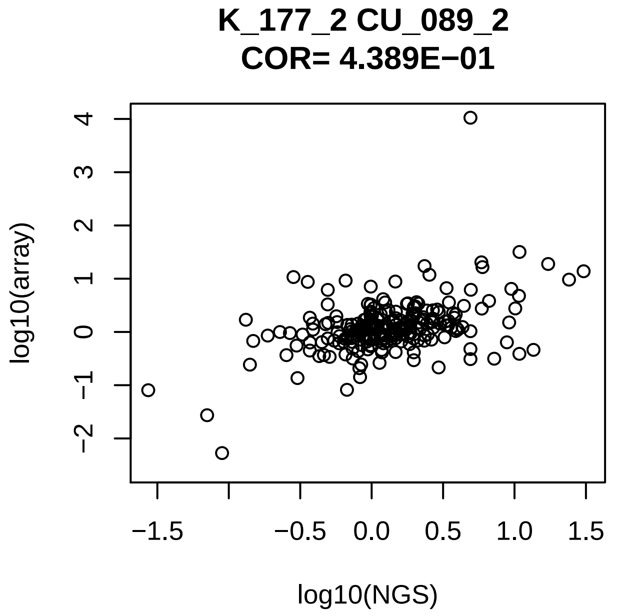
K_177_2 CU_089_2
COR= 4.389E−01
log10(array)
log10(NGS)
−2
−1
0
1
2
3
4
−1.5
−0.5
0.0
0.5
1.0
1.5

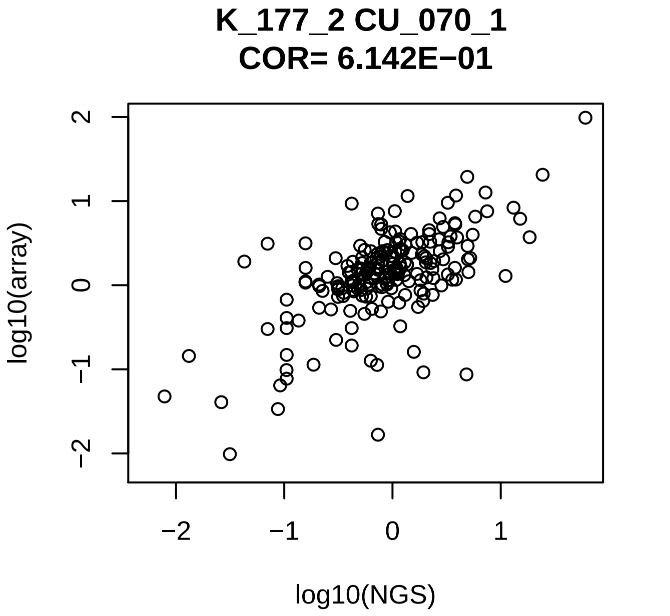
K_177_2 CU_070_1
COR= 6.142E−01
log10(array)
log10(NGS)
−2
−1
0
1
2
−2
−1
0
1

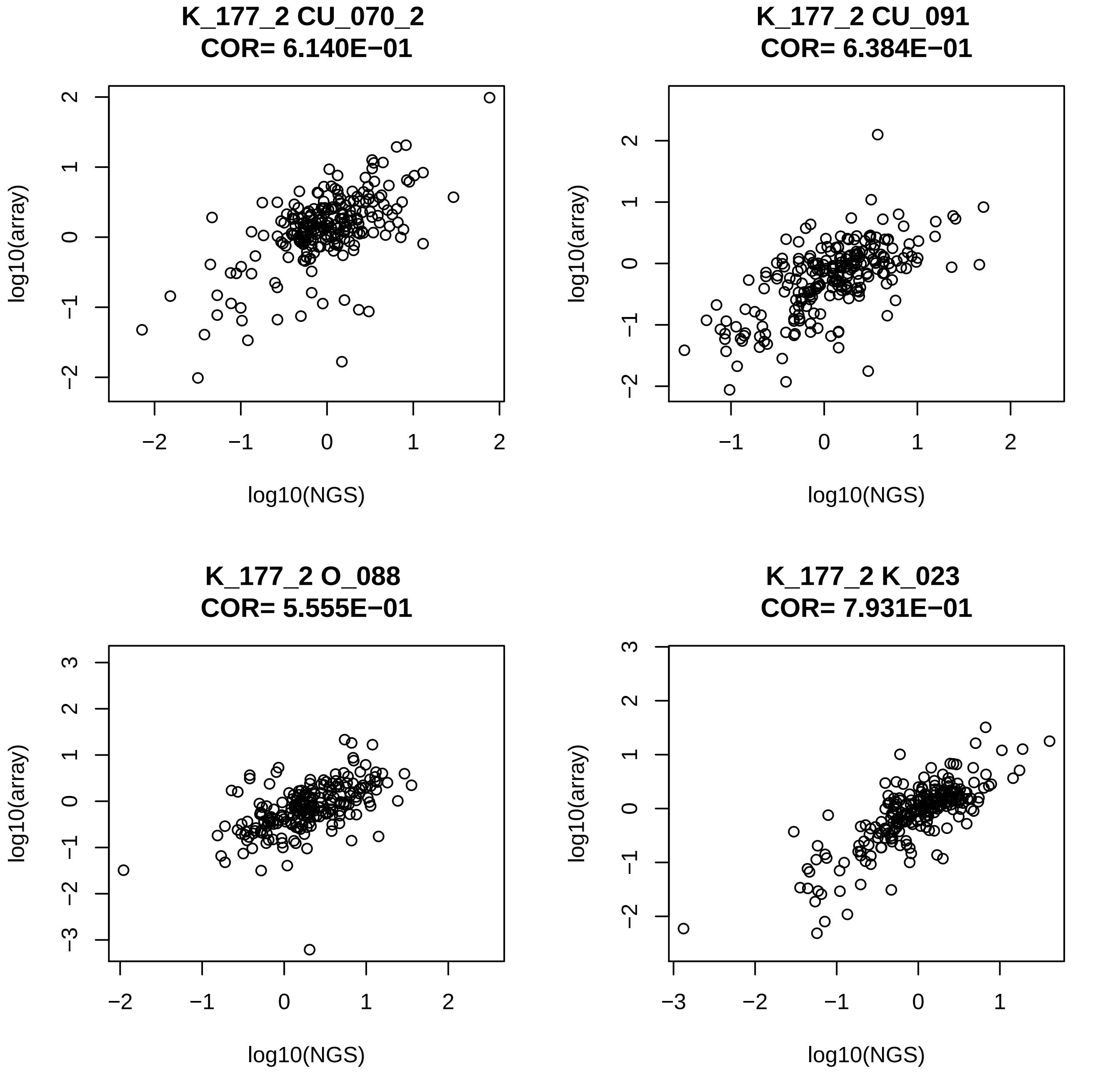
K_177_2 CU_070_2
COR= 6.140E−01
K_177_2 CU_091
COR= 6.384E−01
K_177_2 O_088
COR= 5.555E−01
K_177_2 K_023
COR= 7.931E−01
log10(array)
log10(NGS)

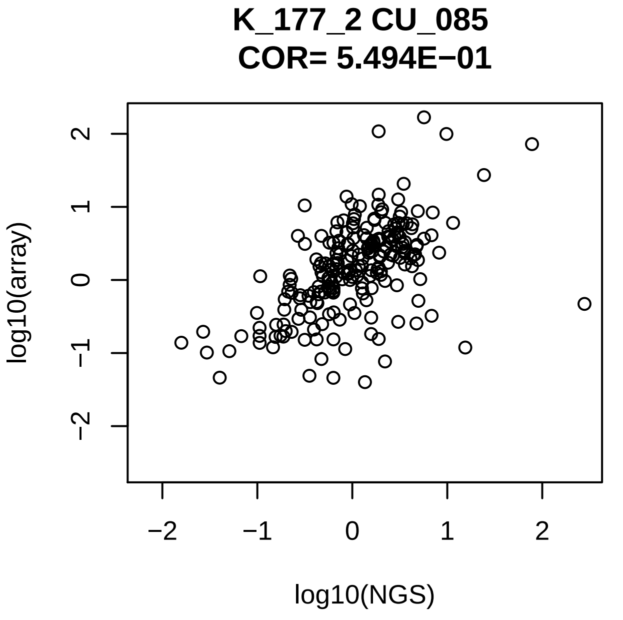
K_177_2 CU_085
COR= 5.494E−01
log10(array)
log10(NGS)
−2
−1
0
1
2

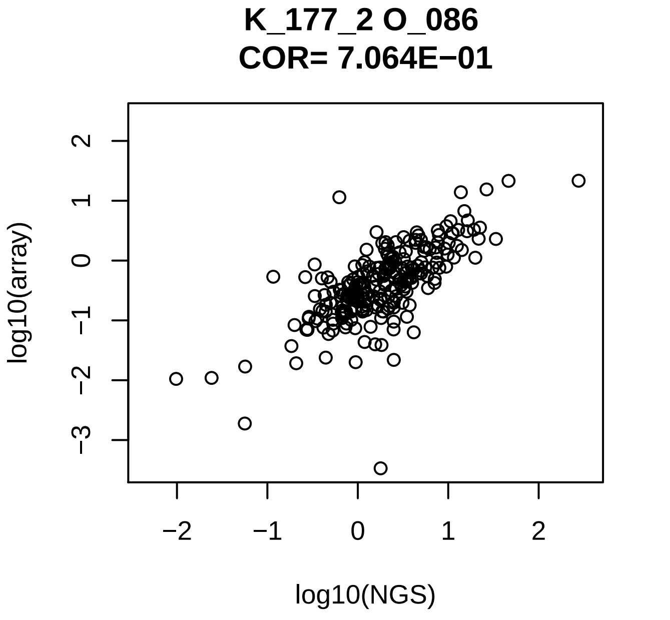
K_177_2 O_086
COR= 7.064E−01
log10(array)
log10(NGS)
−3
−2
−1
0
1
2

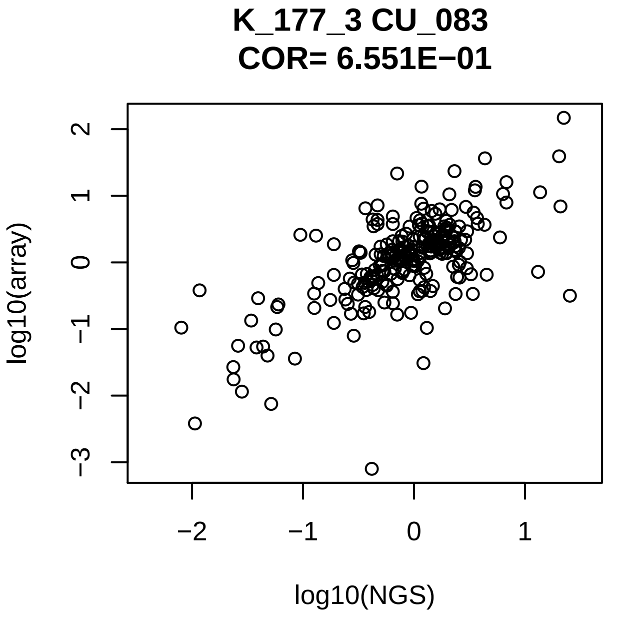
K_177_3 CU_083
COR= 6.551E−01
log10(array)
log10(NGS)
−3
−2
−1
0
1
2

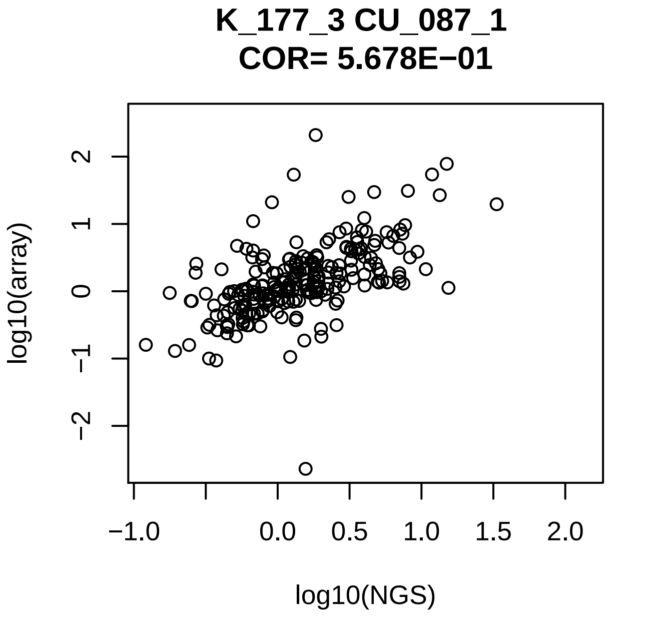
K_177_3 CU_087_1
COR= 5.678E−01
log10(array)
log10(NGS)
−2
−1
0
1
2
−1.0
0.0
0.5
1.0
1.5
2.0

K_177_3 CU_087_2
COR= 5.318E−01

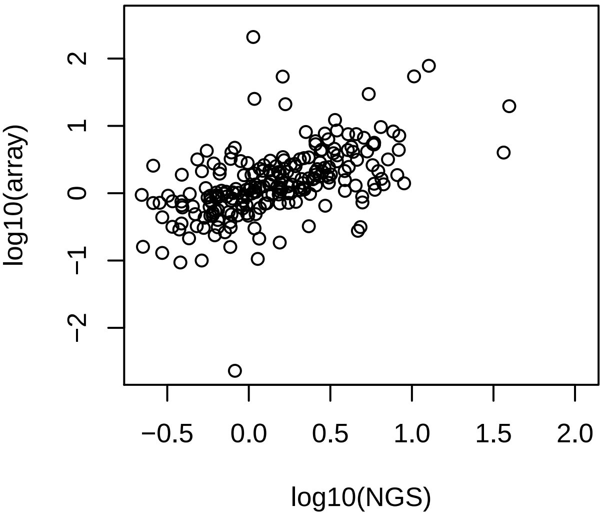

K_177_3 CU_087_3
COR= 5.774E−01

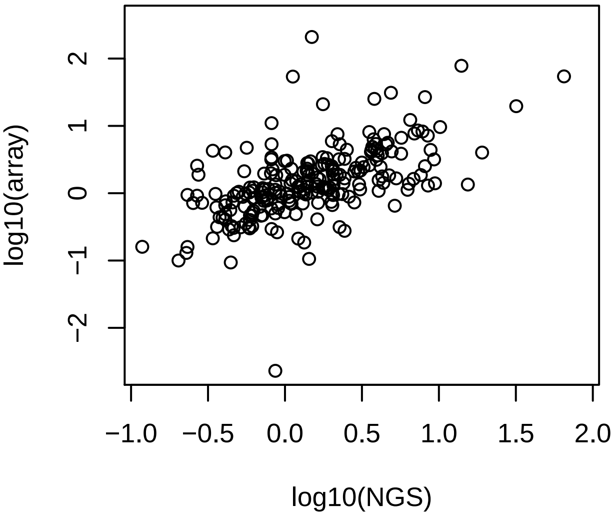

K_177_3 CU_089_1
COR= 6.002E−01

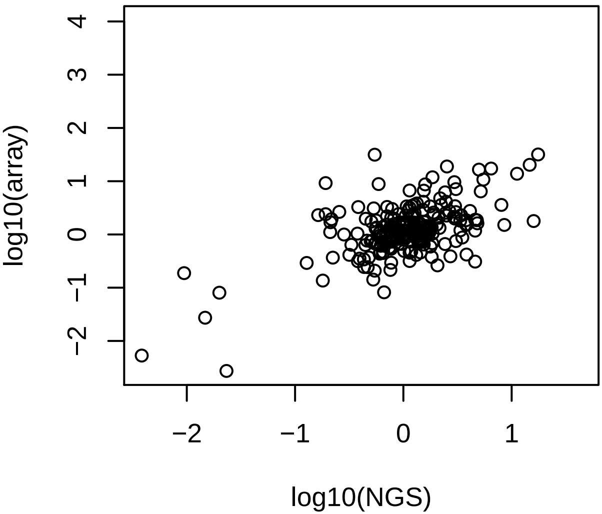

K_177_3 CU_089_2
COR= 4.326E−01

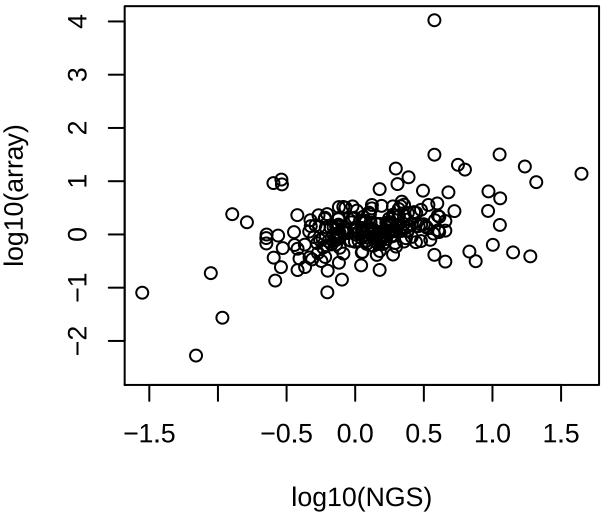

K_177_3 CU_070_1
COR= 6.325E−01

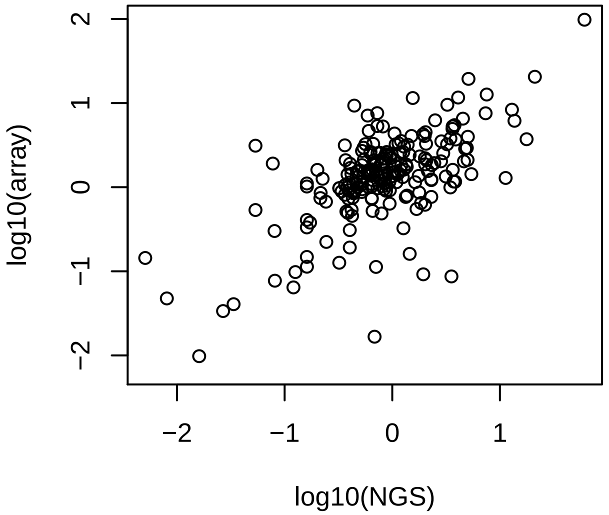

K_177_3 CU_070_2
COR= 6.223E−01

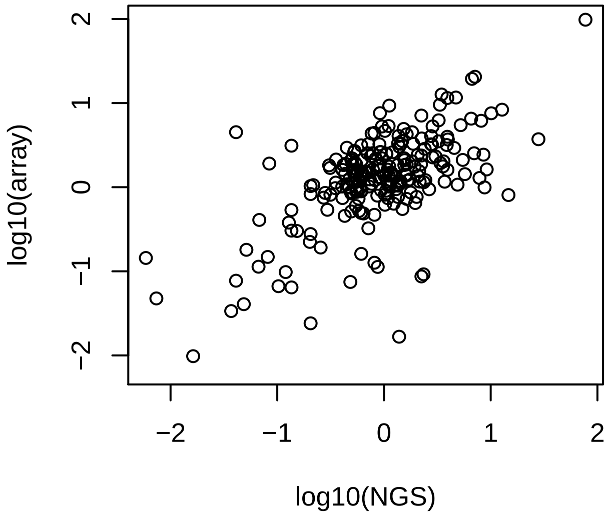

K_177_3 CU_091
COR= 6.534E−01

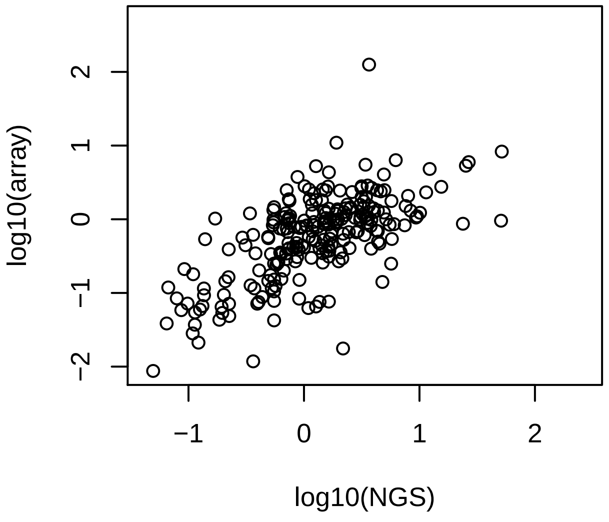

K_177_3 O_088
COR= 5.443E−01

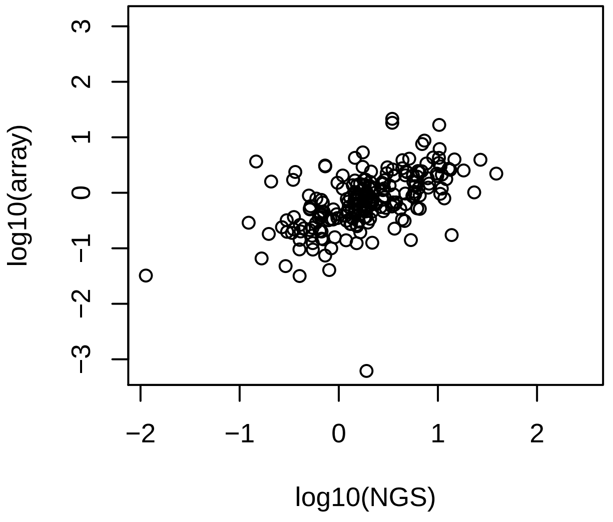

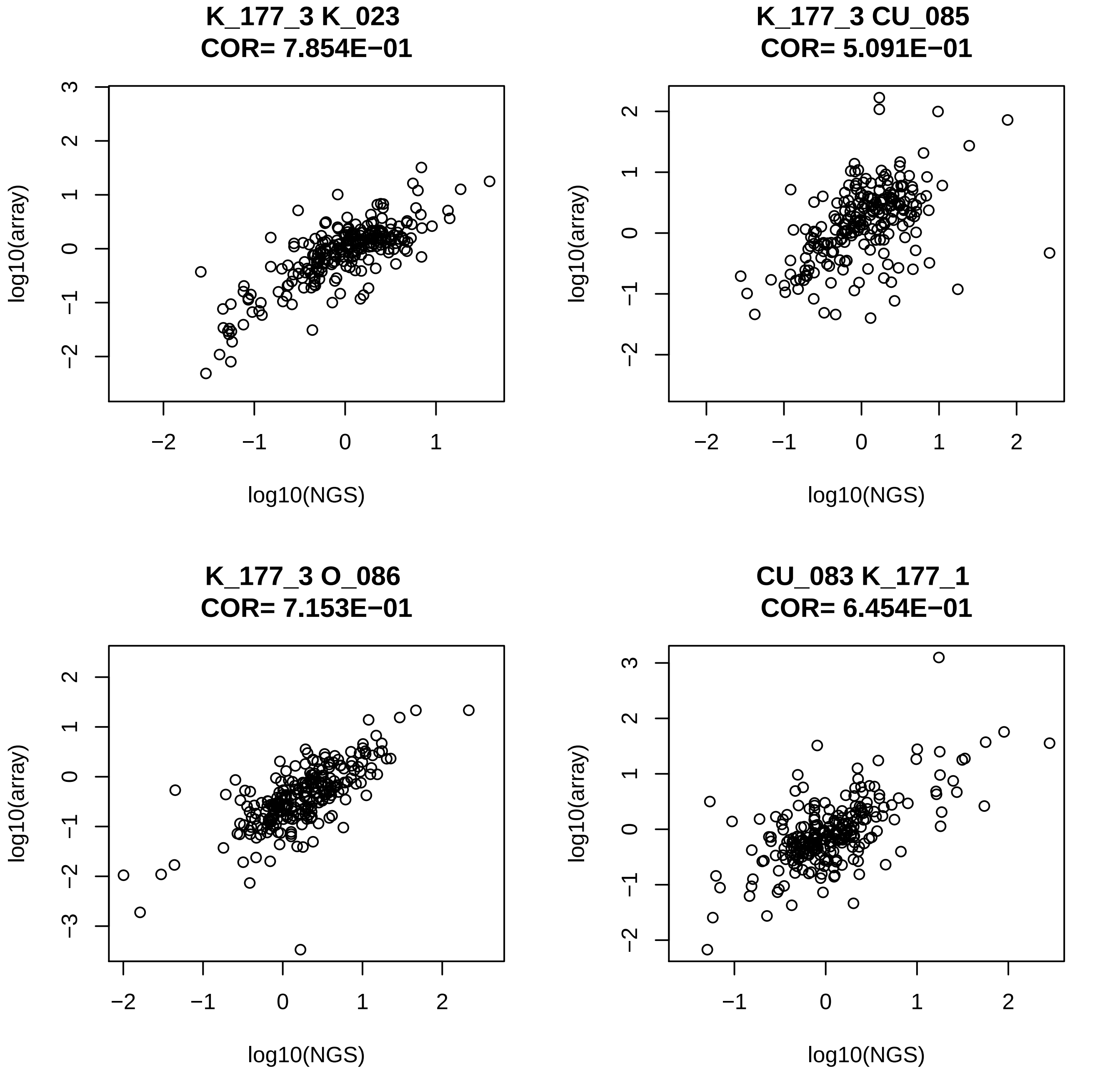
K_177_3 K_023
COR= 7.854E−01
K_177_3 CU_085
COR= 5.091E−01
K_177_3 O_086
COR= 7.153E−01
CU_083 K_177_1
COR= 6.454E−01
log10(array)
log10(NGS)

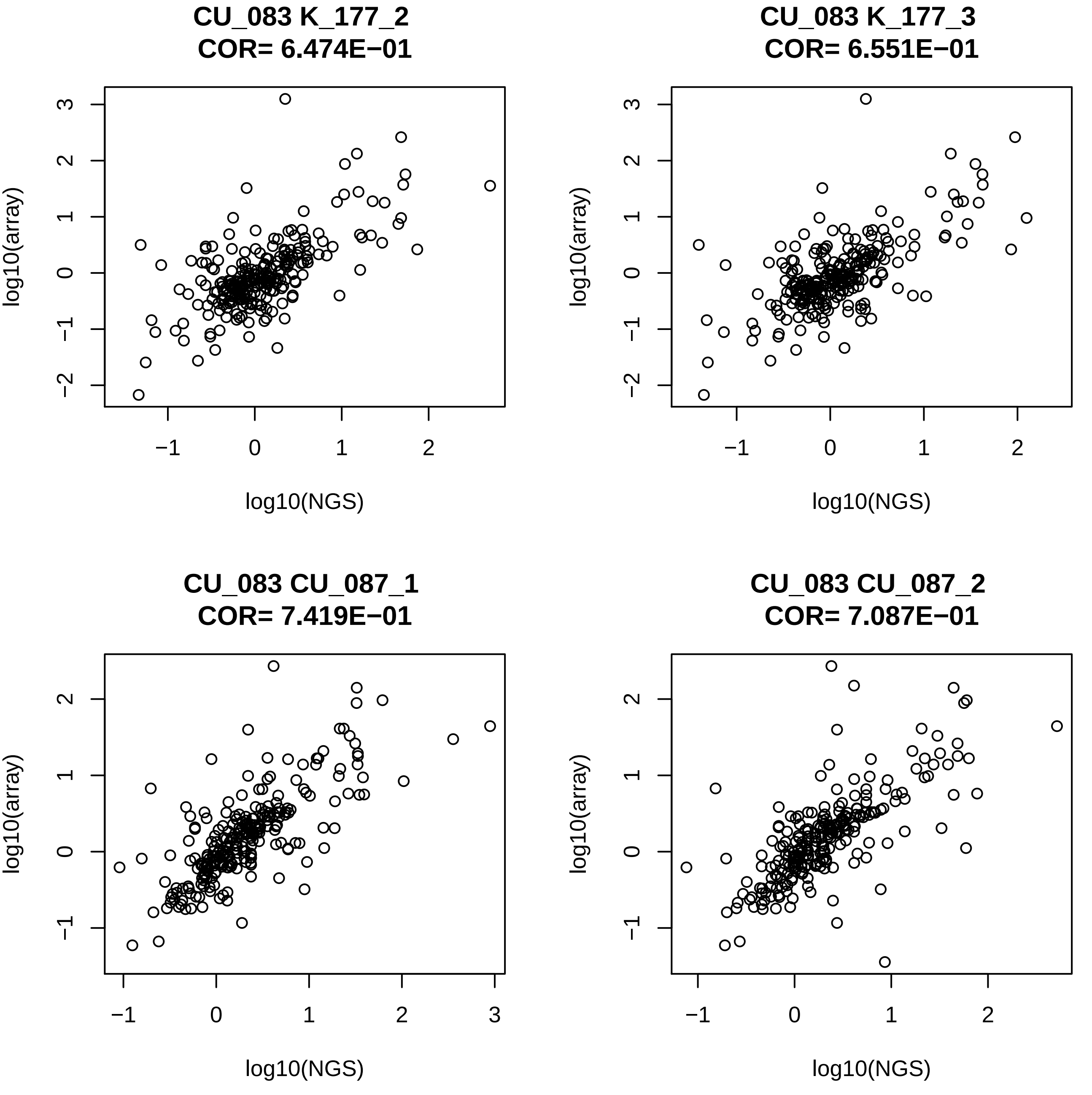
CU_083 K_177_2
COR= 6.474E−01
CU_083 K_177_3
COR= 6.551E−01
CU_083 CU_087_1
COR= 7.419E−01
CU_083 CU_087_2
COR= 7.087E−01
log10(array)
log10(NGS)

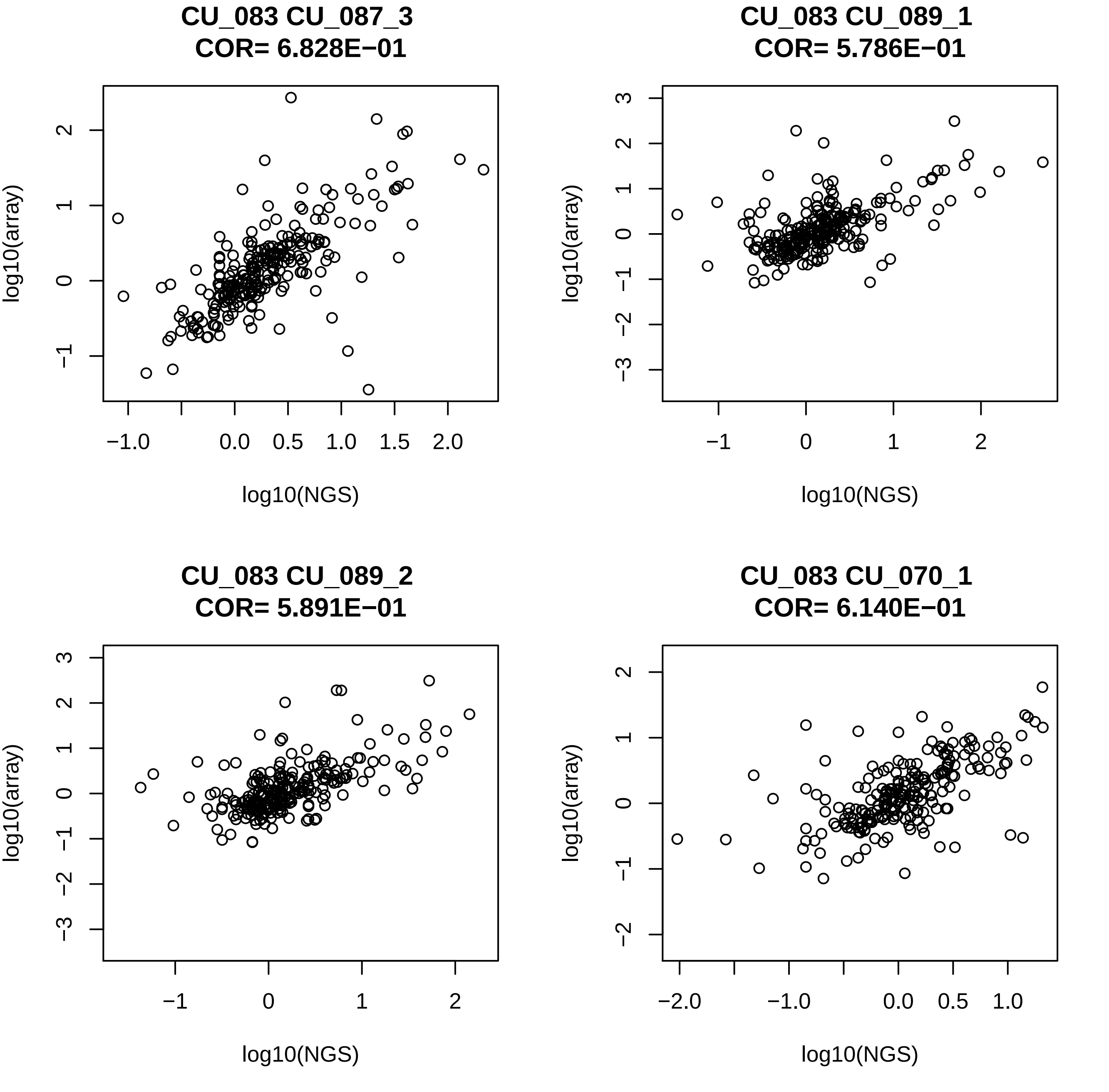
CU_083 CU_087_3
COR= 6.828E−01
log10(array)
log10(NGS)
CU_083 CU_089_1
COR= 5.786E−01
log10(array)
log10(NGS)
CU_083 CU_089_2
COR= 5.891E−01
log10(array)
log10(NGS)
CU_083 CU_070_1
COR= 6.140E−01
log10(array)
log10(NGS)

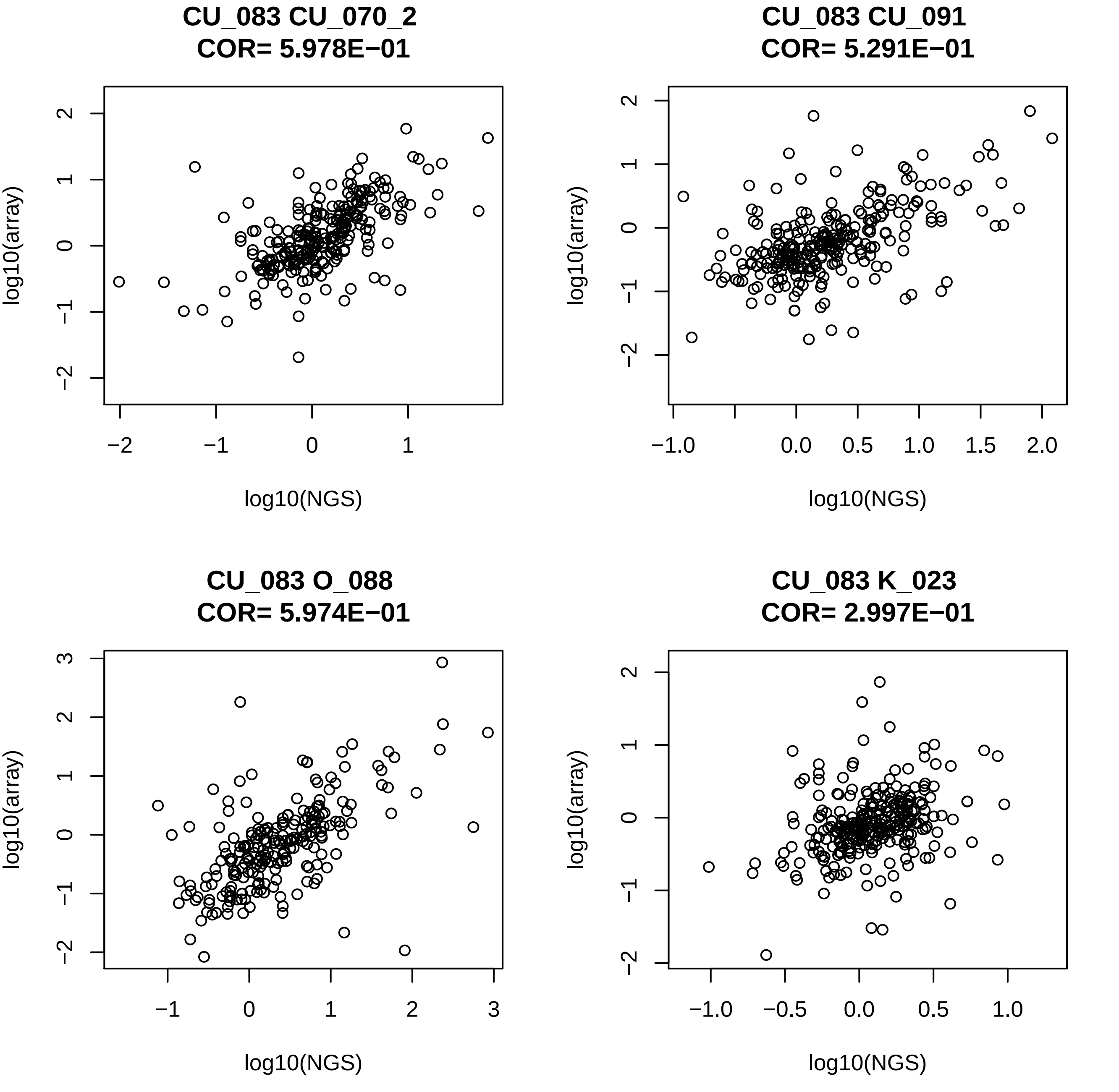
CU_083 CU_070_2
COR= 5.978E−01
log10(array)
log10(NGS)
CU_083 CU_091
COR= 5.291E−01
log10(array)
log10(NGS)
CU_083 O_088
COR= 5.974E−01
log10(array)
log10(NGS)
CU_083 K_023
COR= 2.997E−01
log10(array)
log10(NGS)

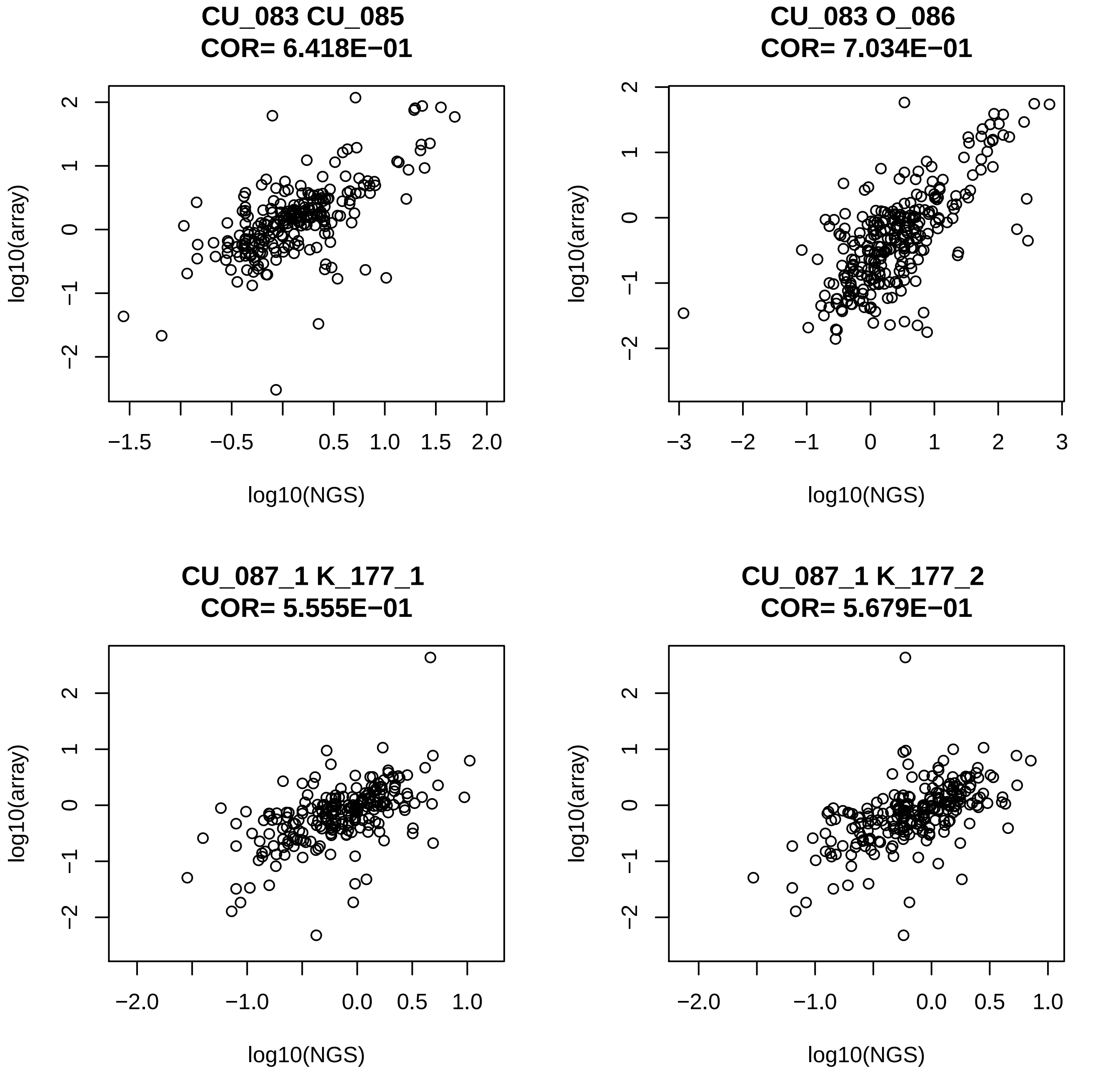
CU_083 CU_085
COR= 6.418E−01
CU_083 O_086
COR= 7.034E−01
CU_087_1 K_177_1
COR= 5.555E−01
CU_087_1 K_177_2
COR= 5.679E−01
log10(array)
log10(NGS)

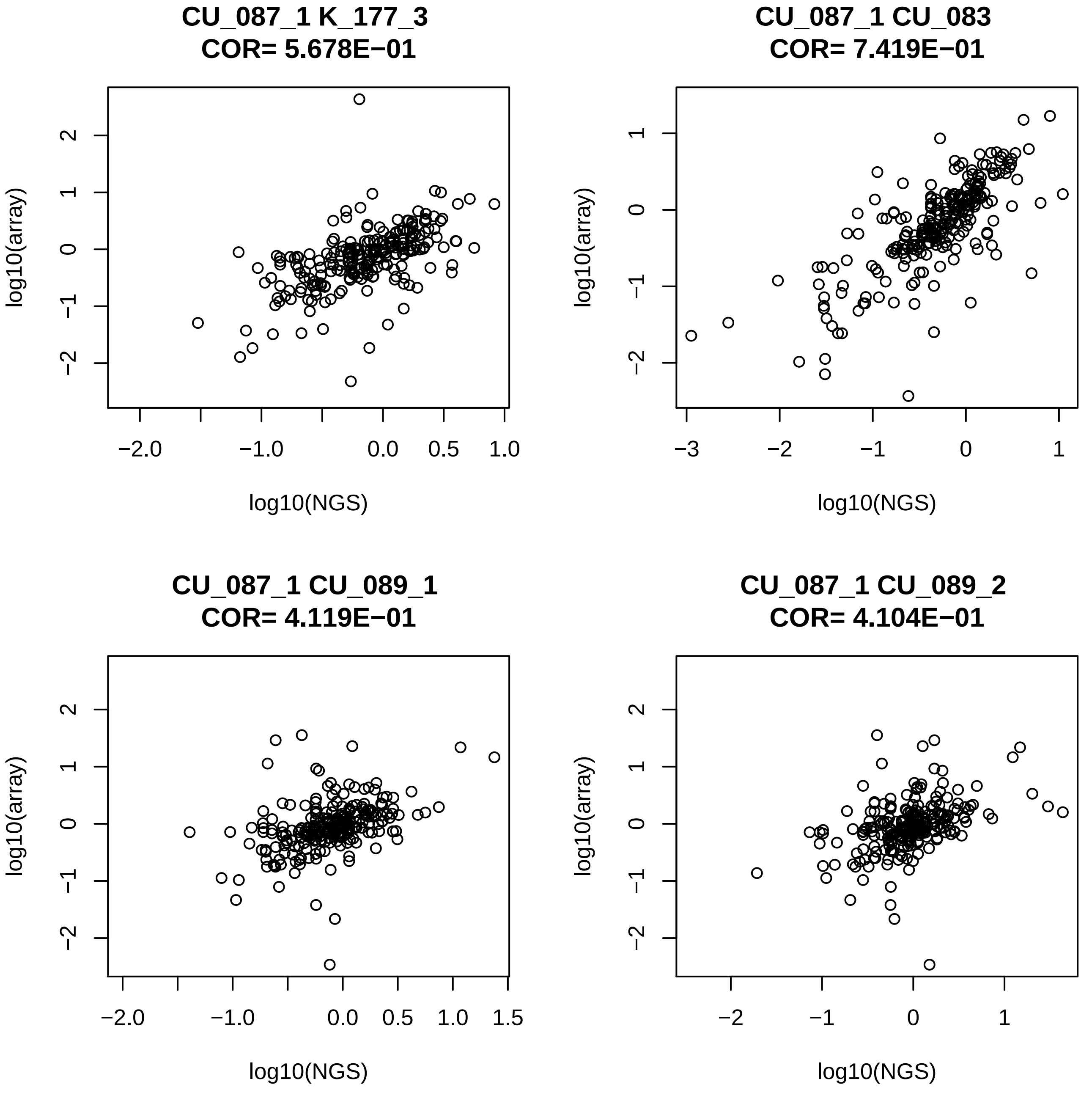
CU_087_1 K_177_3
COR= 5.678E−01
log10(array)
log10(NGS)
−2.0
−1.0
0.0
0.5
1.0
−2
−1
0
1
2
CU_087_1 CU_083
COR= 7.419E−01
log10(array)
log10(NGS)
−3
−2
−1
0
1
CU_087_1 CU_089_1
COR= 4.119E−01
log10(array)
log10(NGS)
−2.0
−1.0
0.0
0.5
1.0
1.5
CU_087_1 CU_089_2
COR= 4.104E−01
log10(array)
log10(NGS)

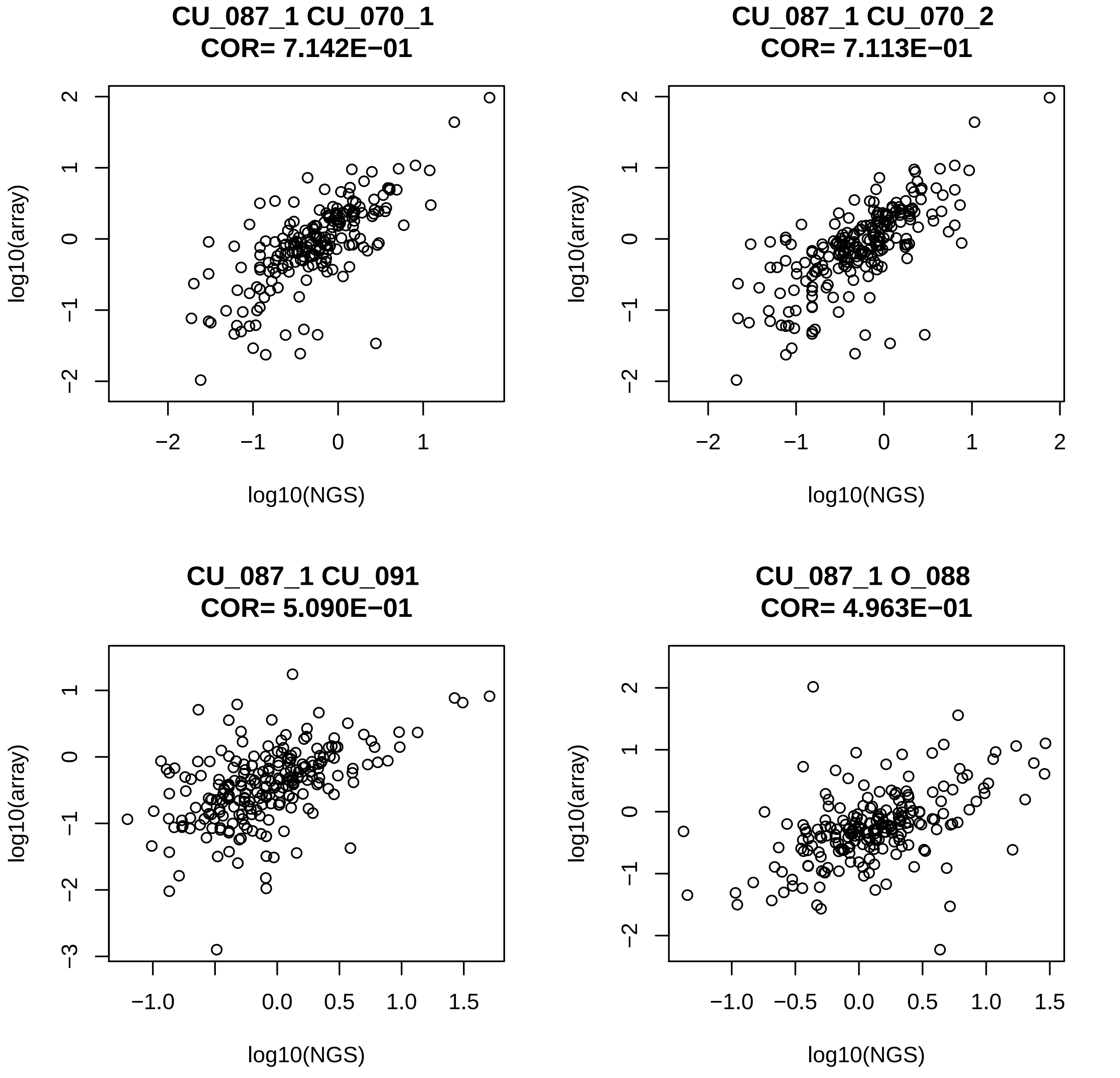
CU_087_1 CU_070_1
COR= 7.142E−01
log10(array)
log10(NGS)
CU_087_1 CU_070_2
COR= 7.113E−01
log10(array)
log10(NGS)
CU_087_1 CU_091
COR= 5.090E−01
log10(array)
log10(NGS)
CU_087_1 O_088
COR= 4.963E−01
log10(array)
log10(NGS)

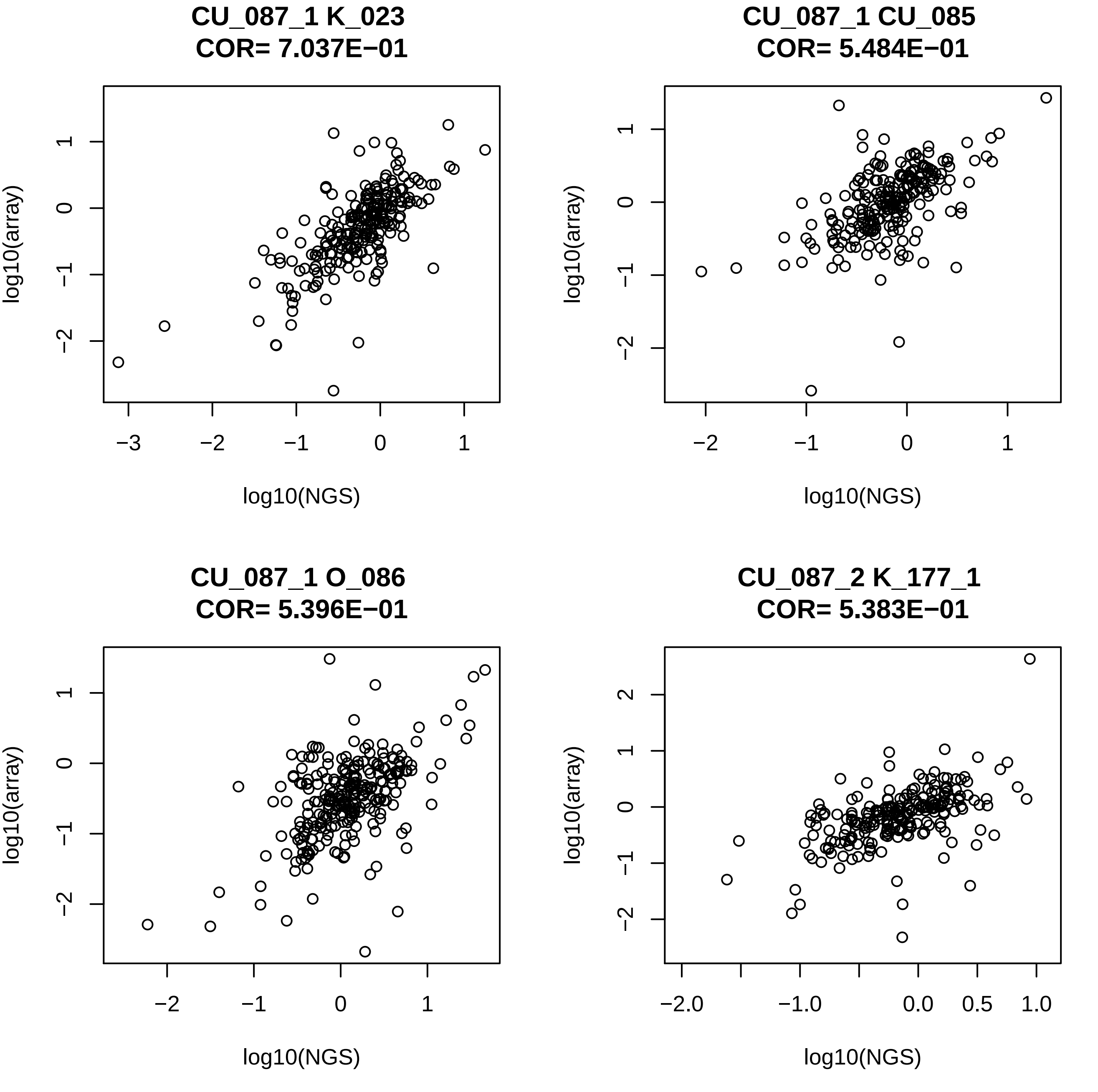
CU_087_1 K_023
COR= 7.037E−01
CU_087_1 CU_085
COR= 5.484E−01
CU_087_1 O_086
COR= 5.396E−01
CU_087_2 K_177_1
COR= 5.383E−01
log10(array)
log10(NGS)

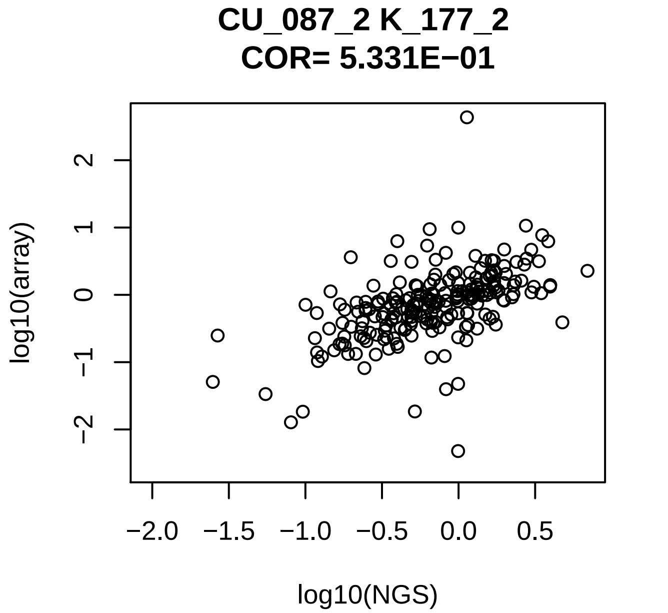
CU_087_2 K_177_2
COR= 5.331E−01
log10(array)
log10(NGS)

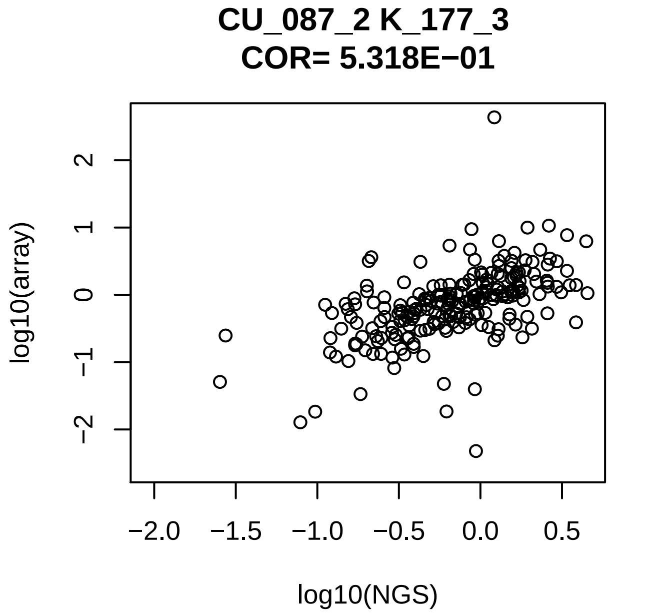
CU_087_2 K_177_3
COR= 5.318E−01
log10(array)
log10(NGS)

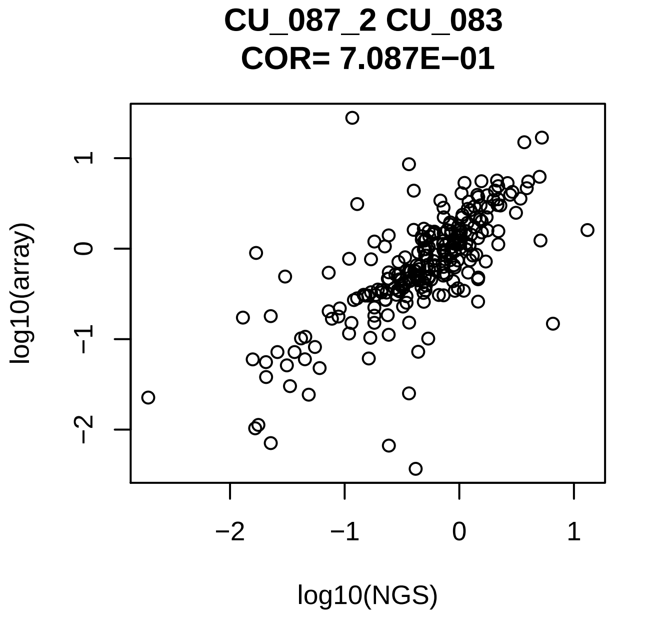
CU_087_2 CU_083
COR= 7.087E−01
log10(array)
log10(NGS)

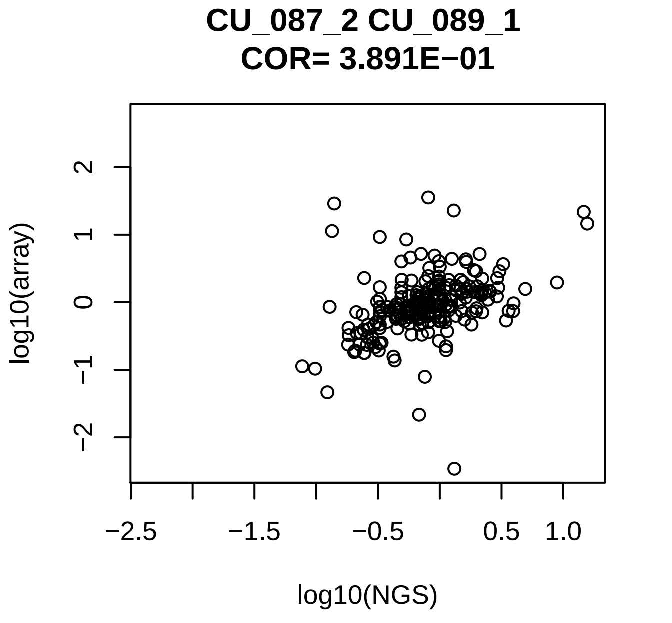
CU_087_2 CU_089_1
COR= 3.891E−01
log10(array)
log10(NGS)

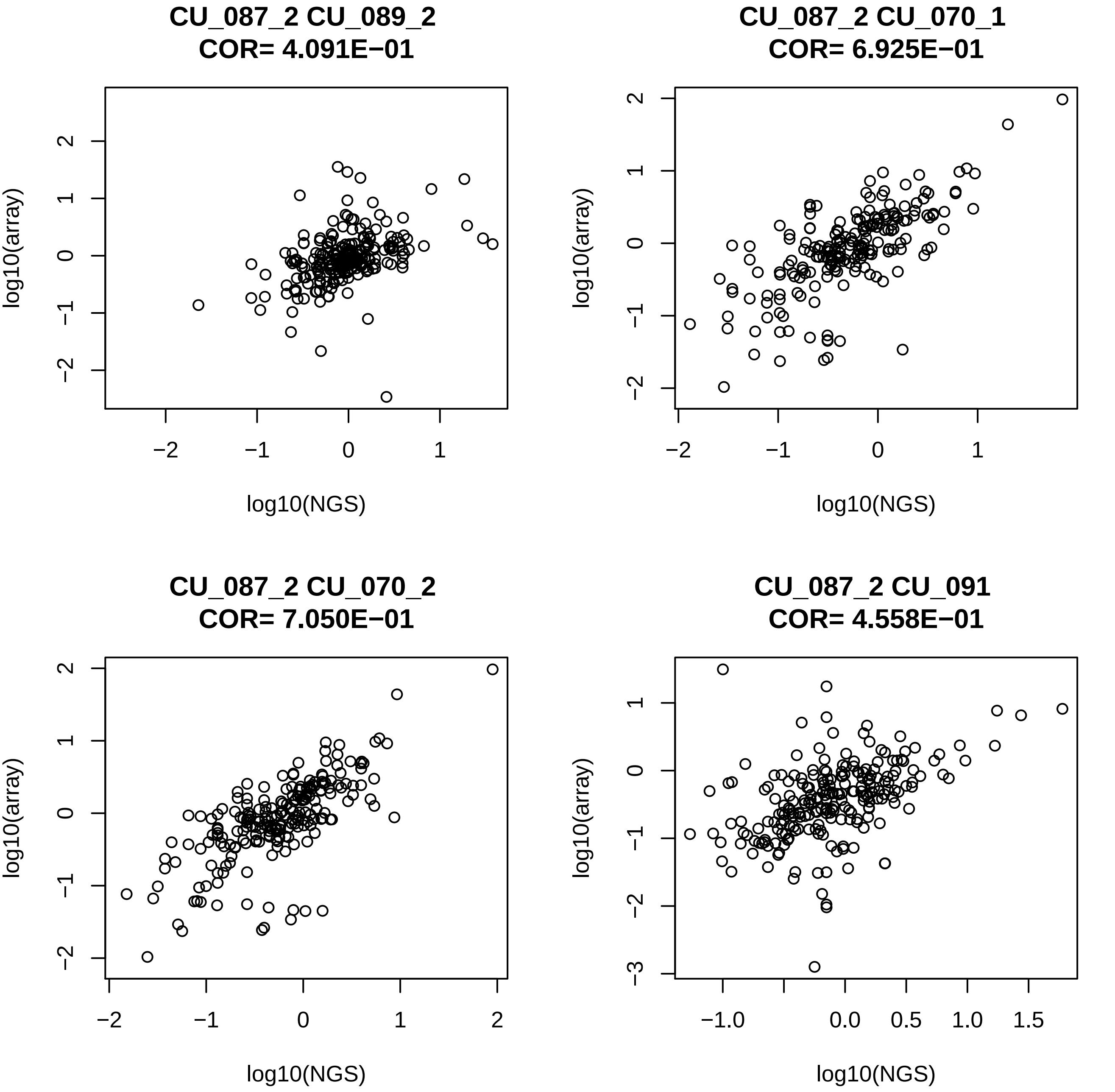
CU_087_2 CU_089_2
COR= 4.091E−01
log10(array)
log10(NGS)
CU_087_2 CU_070_1
COR= 6.925E−01
log10(array)
log10(NGS)
CU_087_2 CU_070_2
COR= 7.050E−01
log10(array)
log10(NGS)
CU_087_2 CU_091
COR= 4.558E−01
log10(array)
log10(NGS)

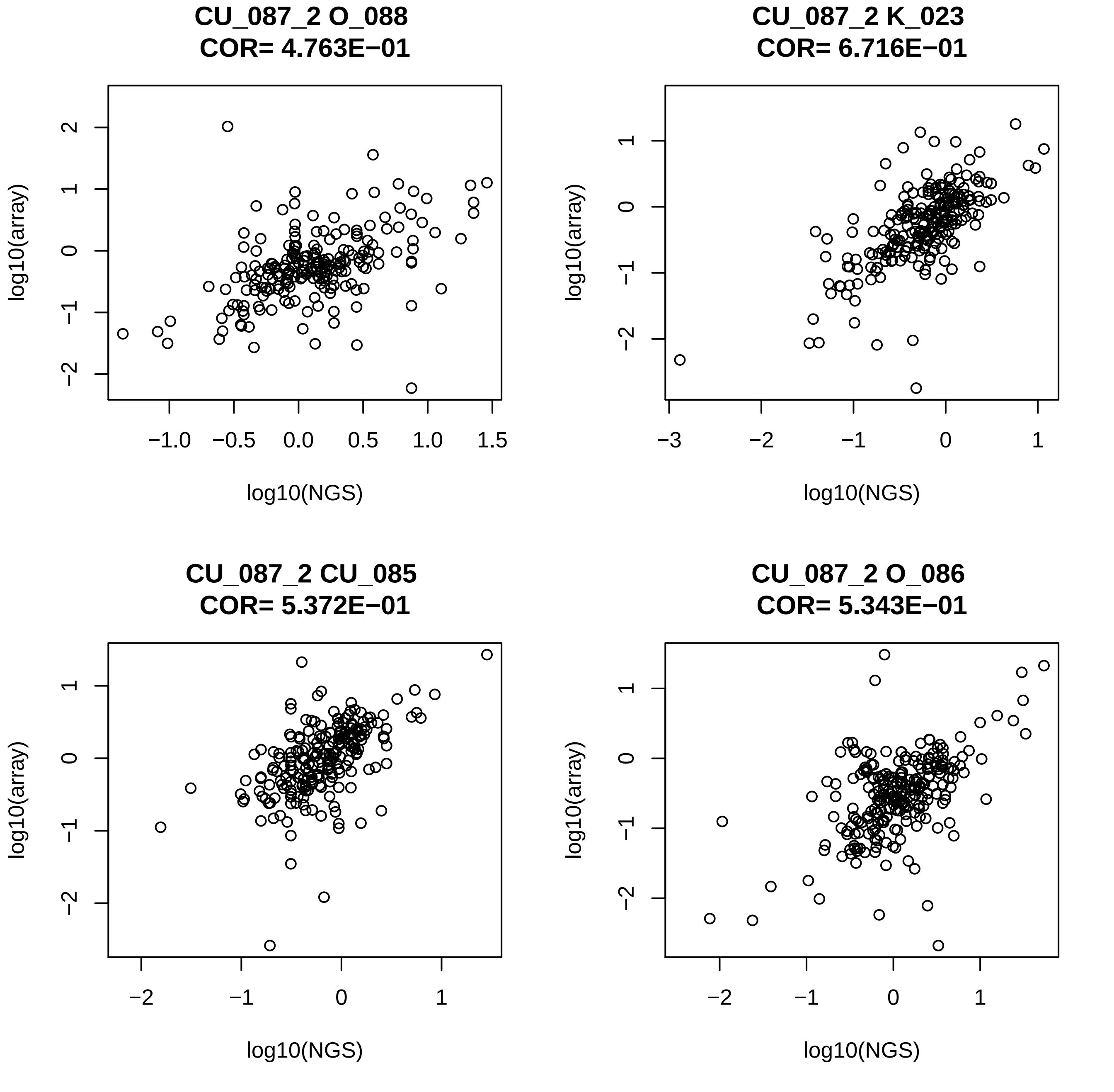
CU_087_2 O_088
COR= 4.763E−01
log10(array)
log10(NGS)
CU_087_2 K_023
COR= 6.716E−01
log10(array)
log10(NGS)
CU_087_2 CU_085
COR= 5.372E−01
log10(array)
log10(NGS)
CU_087_2 O_086
COR= 5.343E−01
log10(array)
log10(NGS)

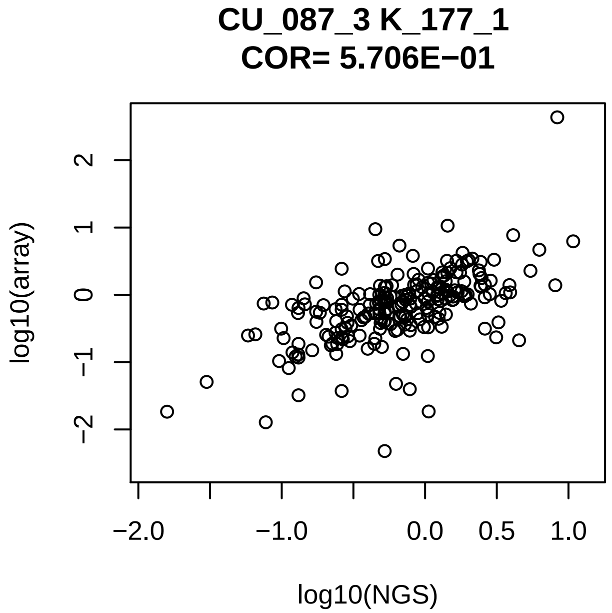
CU_087_3 K_177_1
COR= 5.706E−01
log10(array)
log10(NGS)
−2
−1
0
1
2
−2.0
−1.0
0.0
0.5
1.0

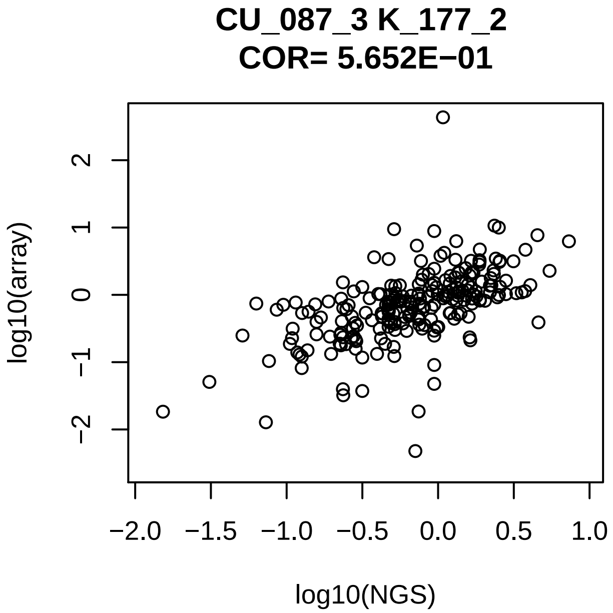
CU_087_3 K_177_2
COR= 5.652E−01
log10(array)
log10(NGS)
−2
−1
0
1
2
−2.0
−1.5
−1.0
−0.5
0.0
0.5
1.0

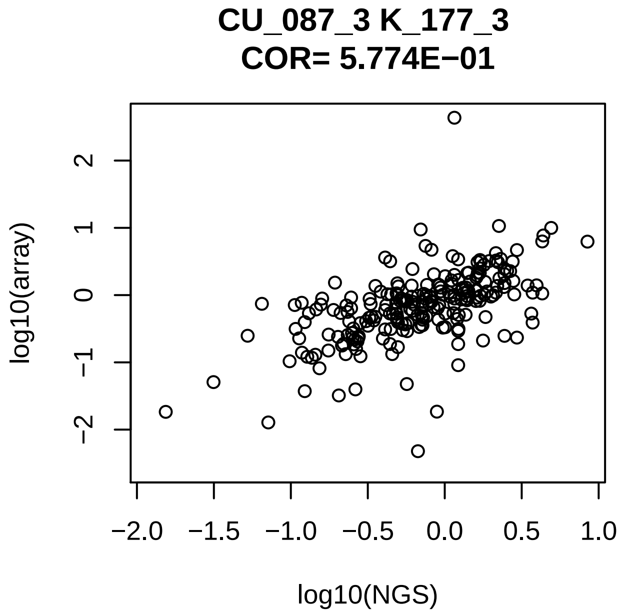
CU_087_3 K_177_3
COR= 5.774E−01
log10(array)
log10(NGS)
−2
−1
0
1
2
−2.0
−1.5
−1.0
−0.5
0.0
0.5
1.0

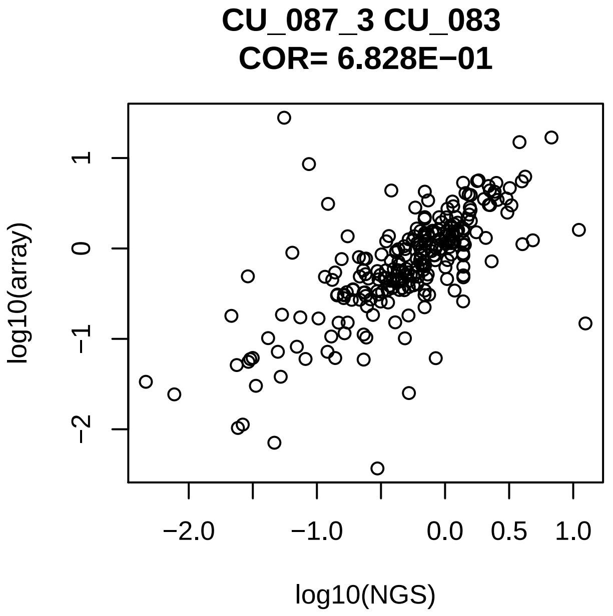
CU_087_3 CU_083
COR= 6.828E−01
log10(array)
log10(NGS)
−2
−1
0
1
−2.0
−1.0
0.0
0.5
1.0

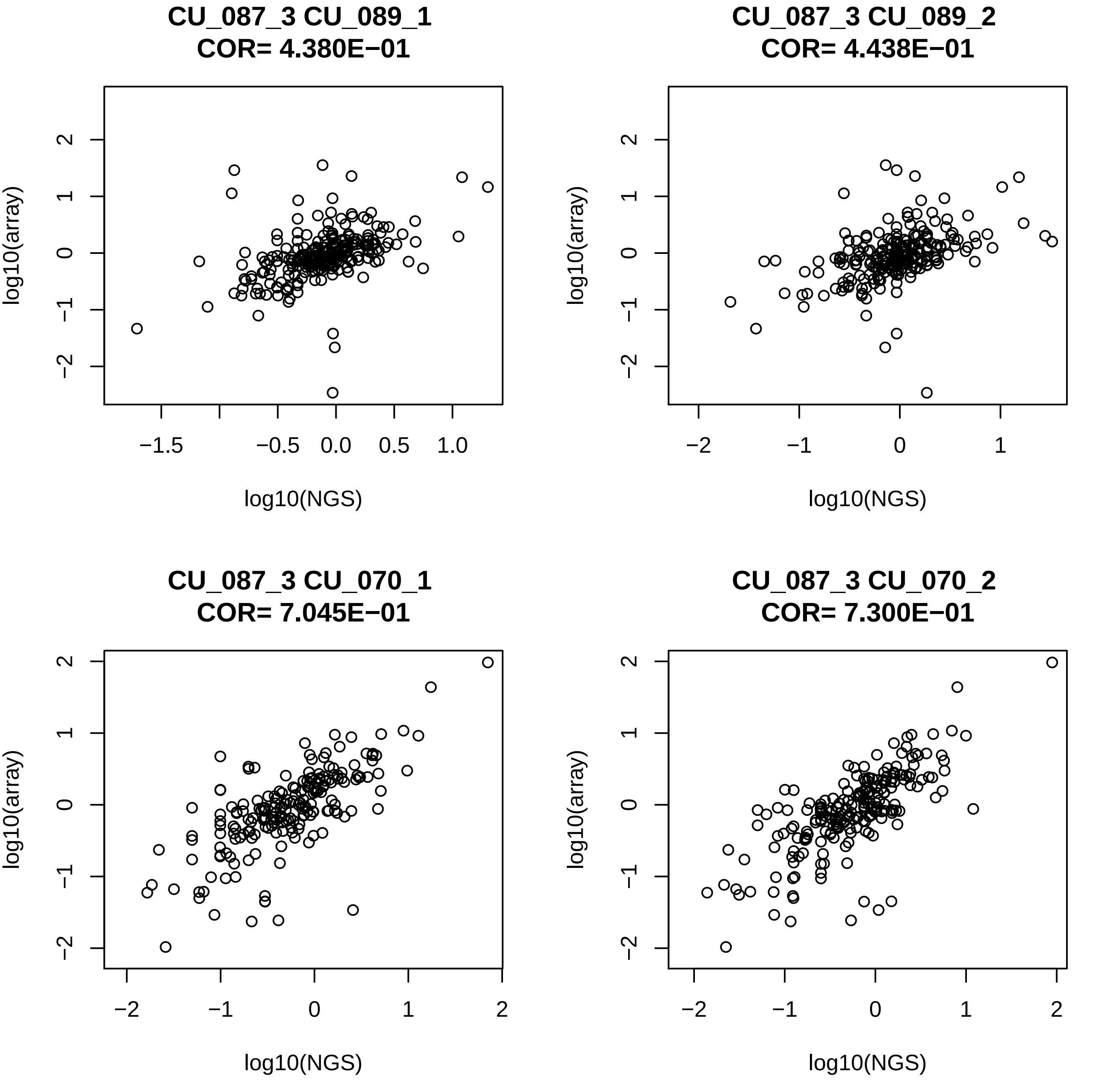
CU_087_3 CU_089_1
COR= 4.380E−01
CU_087_3 CU_089_2
COR= 4.438E−01
CU_087_3 CU_070_1
COR= 7.045E−01
CU_087_3 CU_070_2
COR= 7.300E−01
log10(array)
log10(NGS)

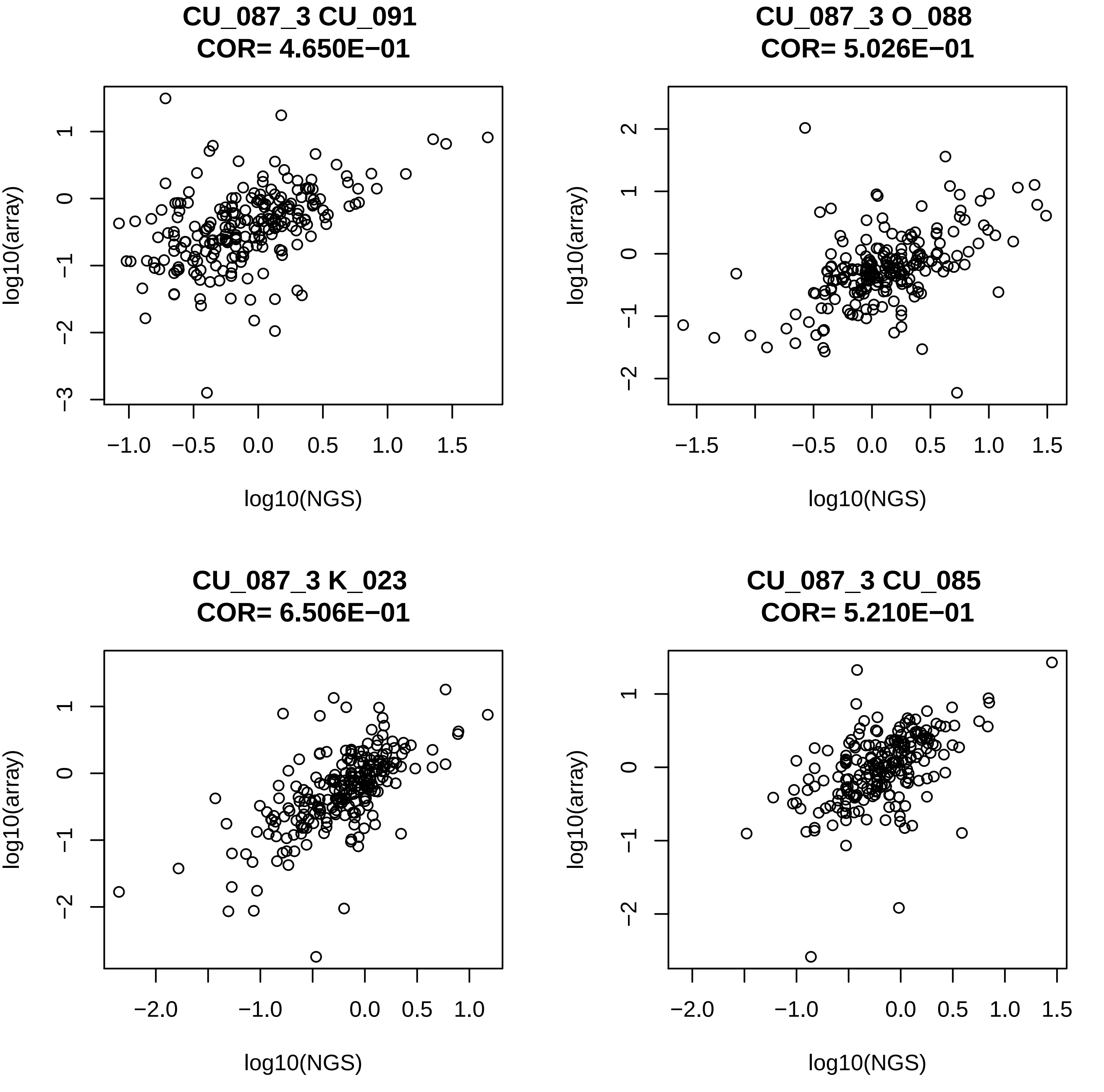
CU_087_3 CU_091
COR= 4.650E−01
CU_087_3 O_088
COR= 5.026E−01
CU_087_3 K_023
COR= 6.506E−01
CU_087_3 CU_085
COR= 5.210E−01
log10(array)
log10(NGS)

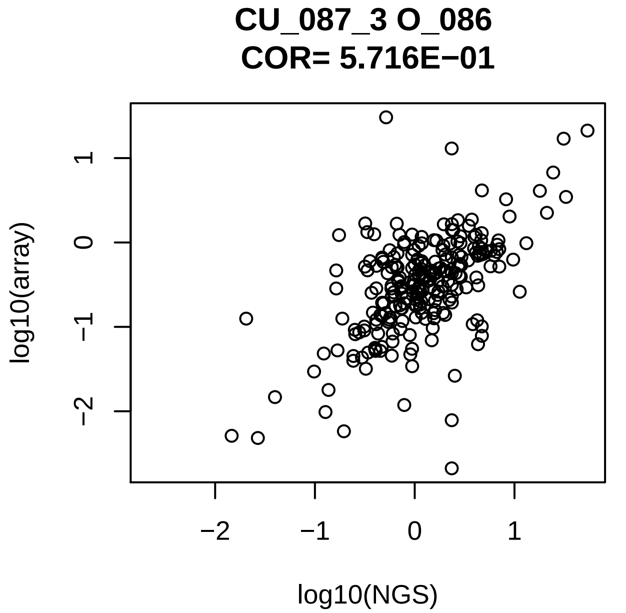
CU_087_3 O_086
COR= 5.716E−01
log10(array)
log10(NGS)

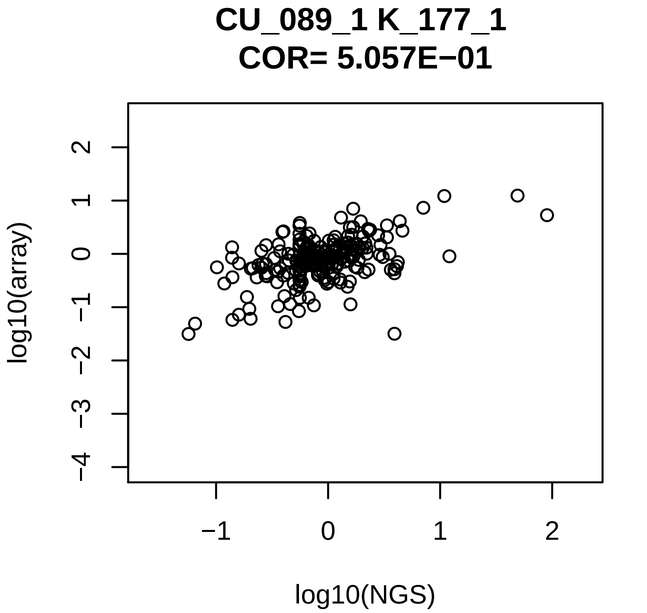
CU_089_1 K_177_1
COR= 5.057E−01
log10(array)
log10(NGS)

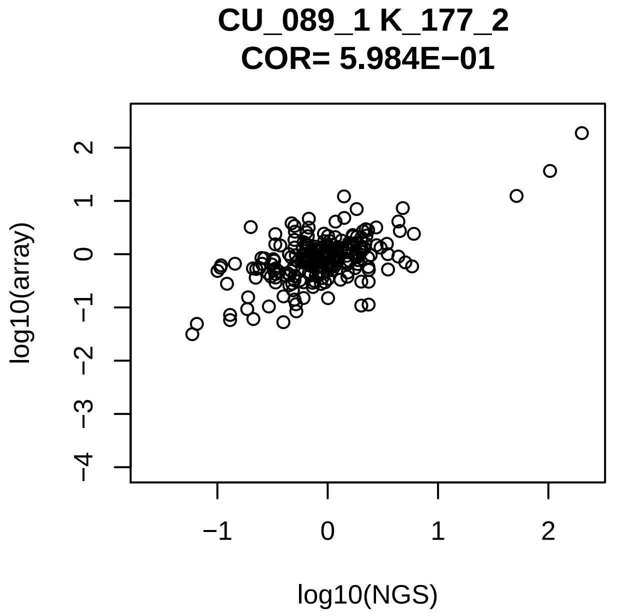
CU_089_1 K_177_2
COR= 5.984E−01
log10(array)
log10(NGS)

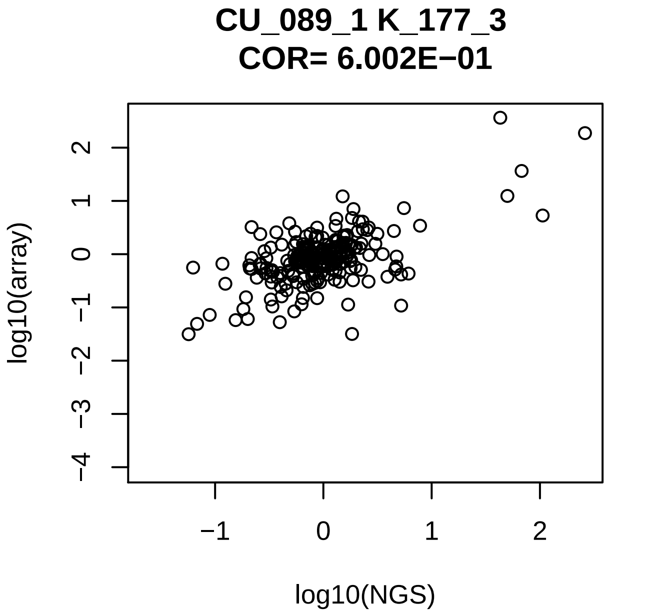
CU_089_1 K_177_3
COR= 6.002E−01
log10(array)
log10(NGS)

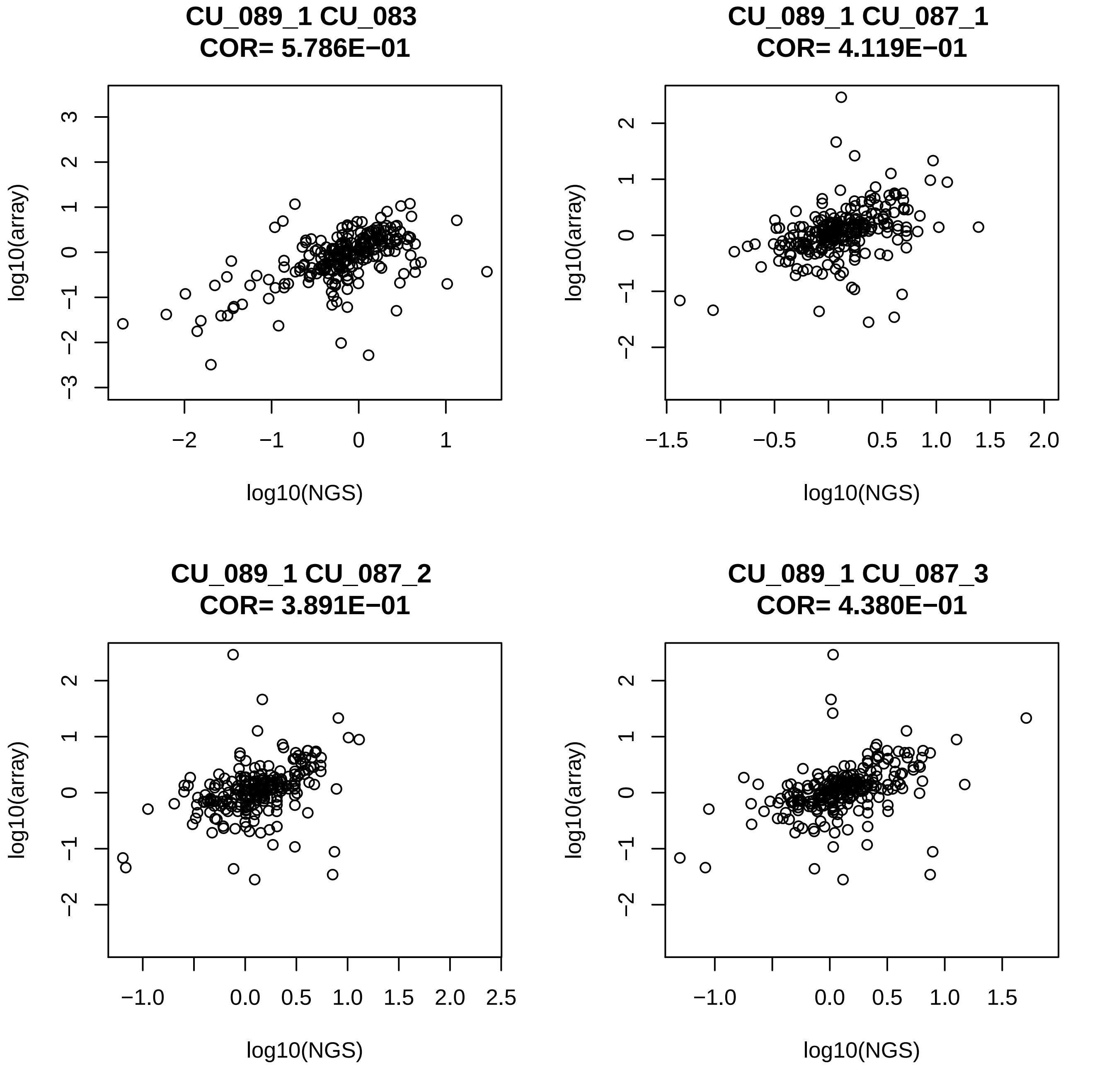
CU_089_1 CU_083
COR= 5.786E−01
log10(array)
log10(NGS)
CU_089_1 CU_087_1
COR= 4.119E−01
log10(array)
log10(NGS)
CU_089_1 CU_087_2
COR= 3.891E−01
log10(array)
log10(NGS)
CU_089_1 CU_087_3
COR= 4.380E−01
log10(array)
log10(NGS)

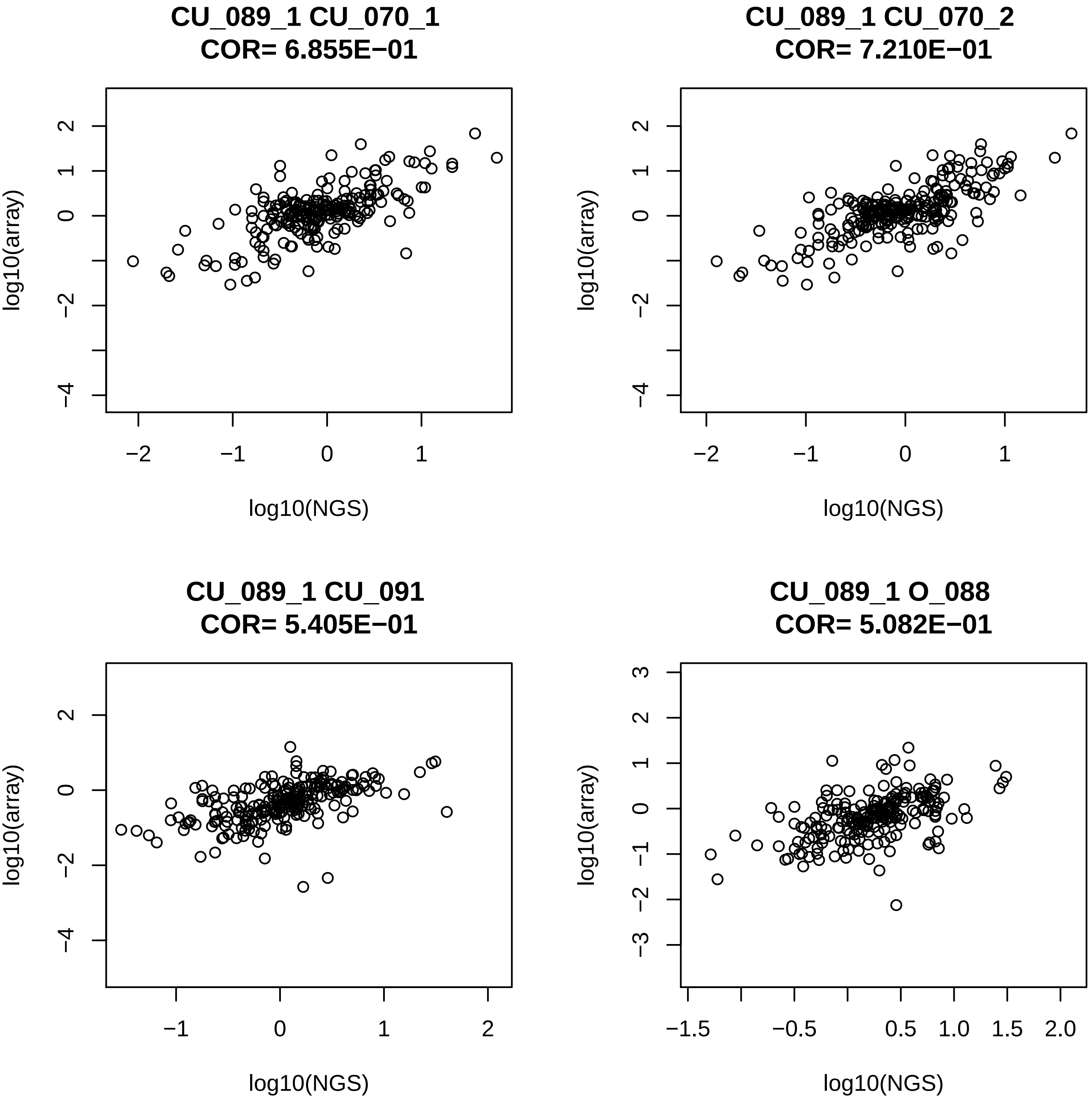
CU_089_1 CU_070_1
COR= 6.855E−01
CU_089_1 CU_070_2
COR= 7.210E−01
CU_089_1 CU_091
COR= 5.405E−01
CU_089_1 O_088
COR= 5.082E−01
log10(array)
log10(NGS)
−2
−1
0
1
2
−4
−3
−1.5
−0.5
0.5
1.0
1.5
2.0
3

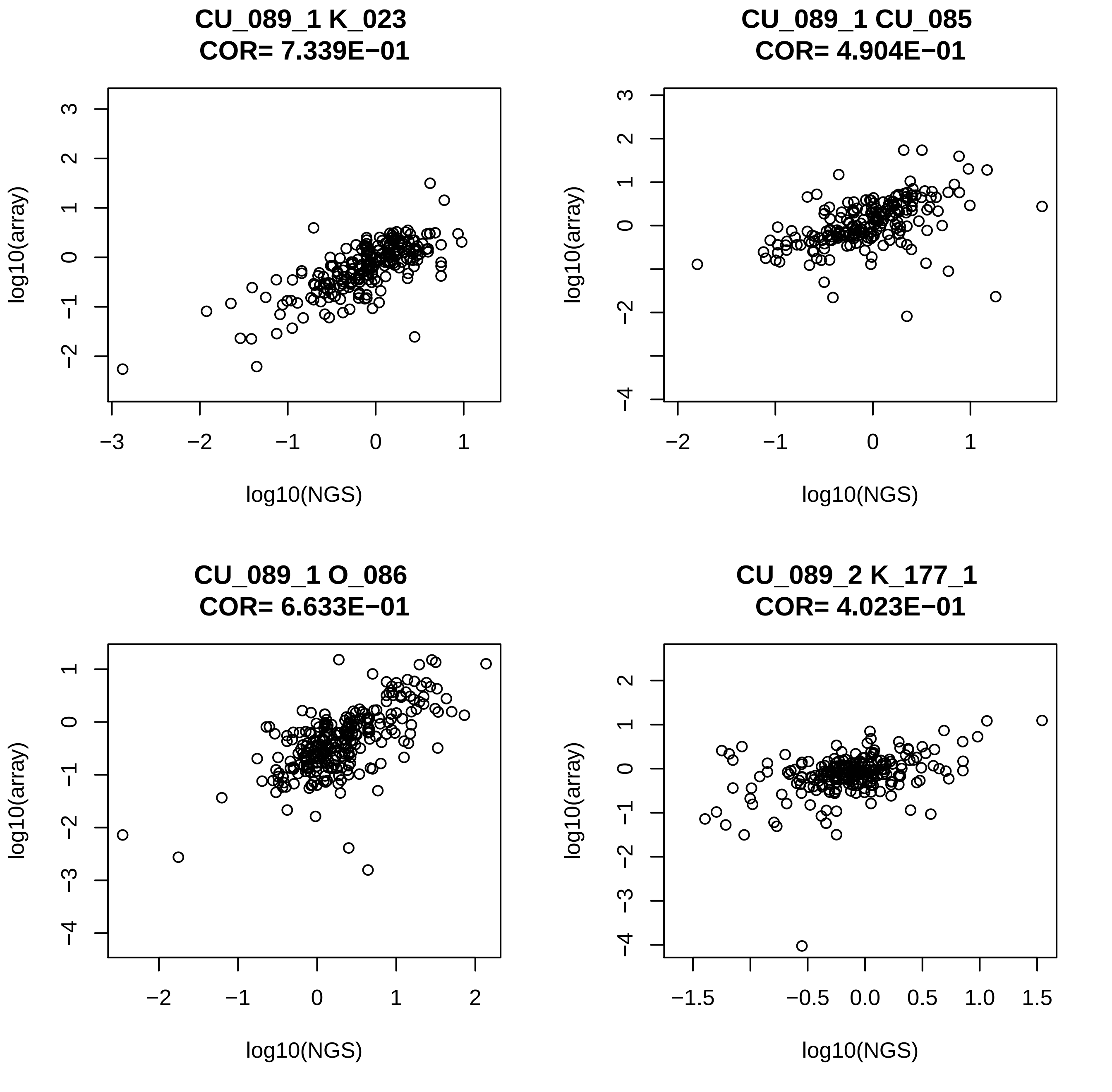
CU_089_1 K_023
COR= 7.339E−01
CU_089_1 CU_085
COR= 4.904E−01
CU_089_1 O_086
COR= 6.633E−01
CU_089_2 K_177_1
COR= 4.023E−01
log10(array)
log10(NGS)

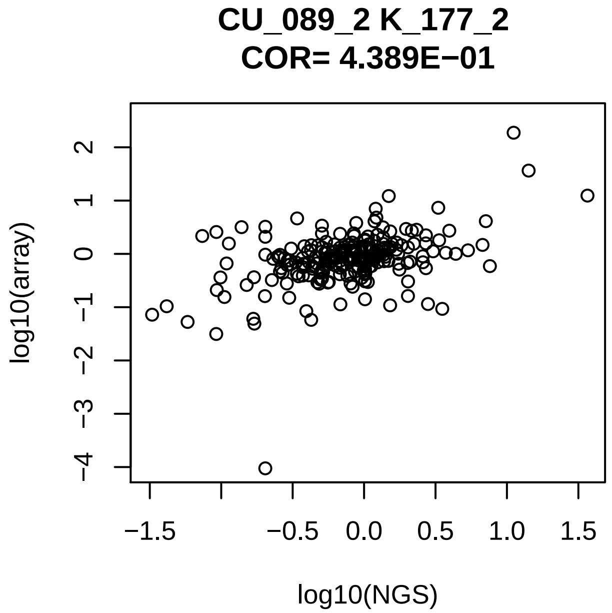
CU_089_2 K_177_2
COR= 4.389E−01
log10(array)
log10(NGS)

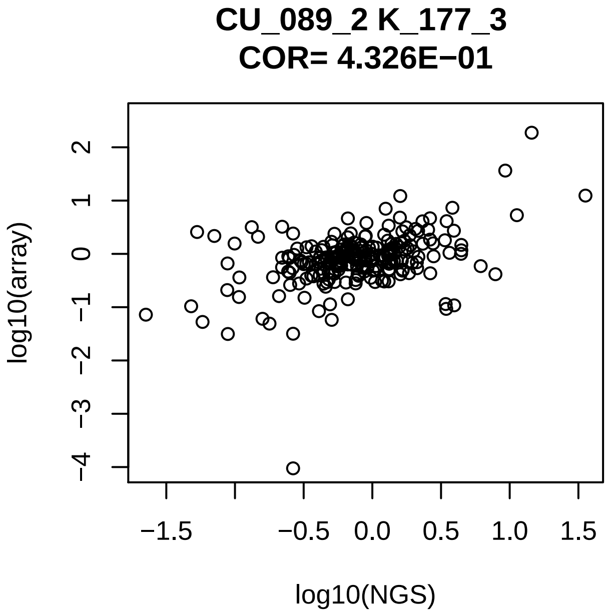
CU_089_2 K_177_3
COR= 4.326E−01
log10(array)
log10(NGS)

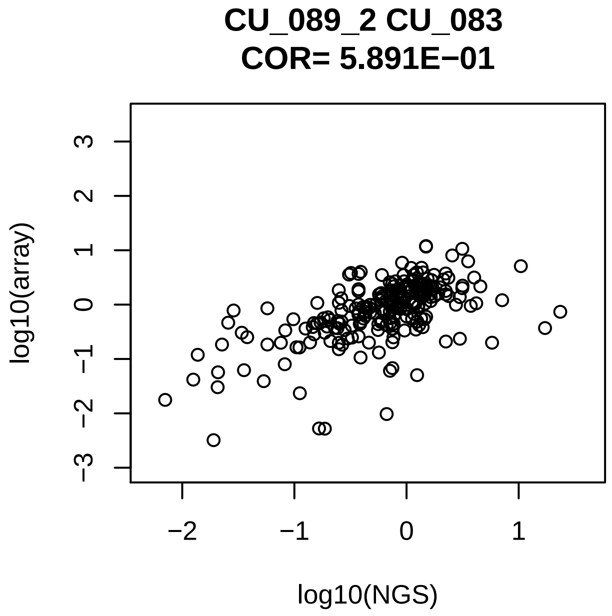
CU_089_2 CU_083
COR= 5.891E−01
log10(array)
log10(NGS)

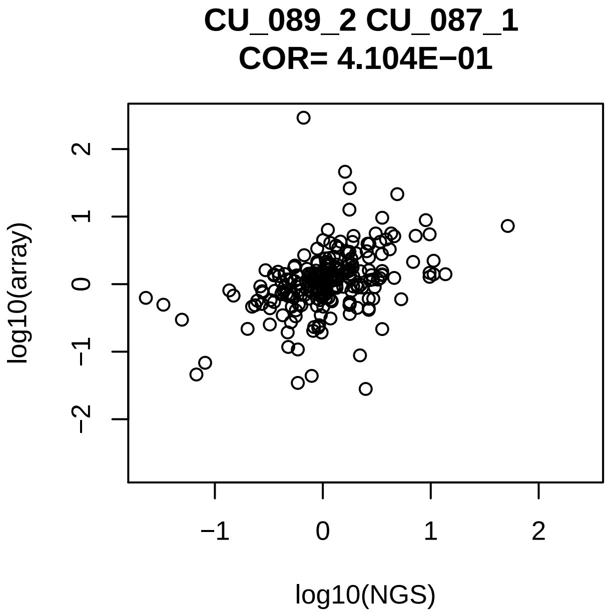
CU_089_2 CU_087_1
COR= 4.104E−01
log10(array)
log10(NGS)

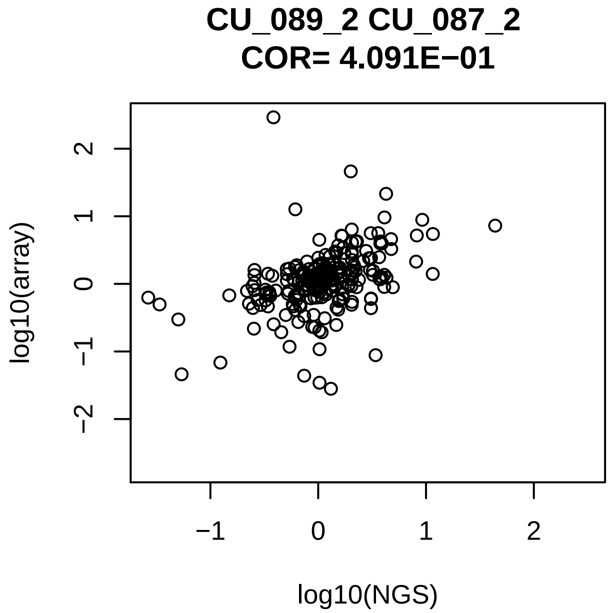

CU_089_2 CU_087_2
COR= 4.091E−01
log10(array)
log10(NGS)

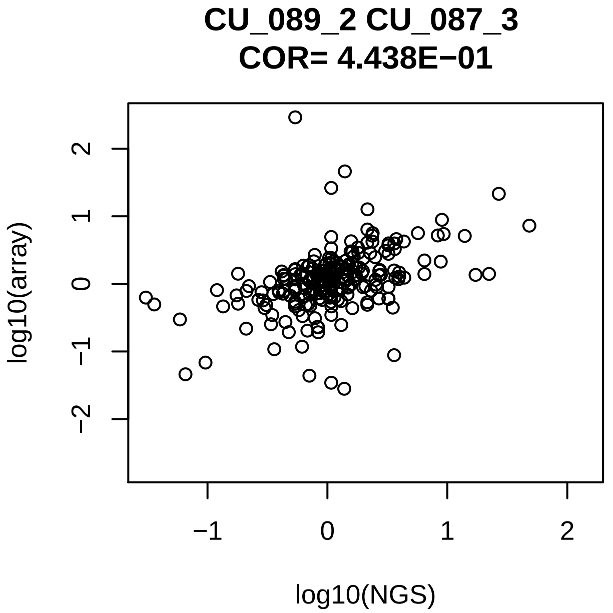

CU_089_2 CU_087_3
COR= 4.438E−01
log10(array)
log10(NGS)

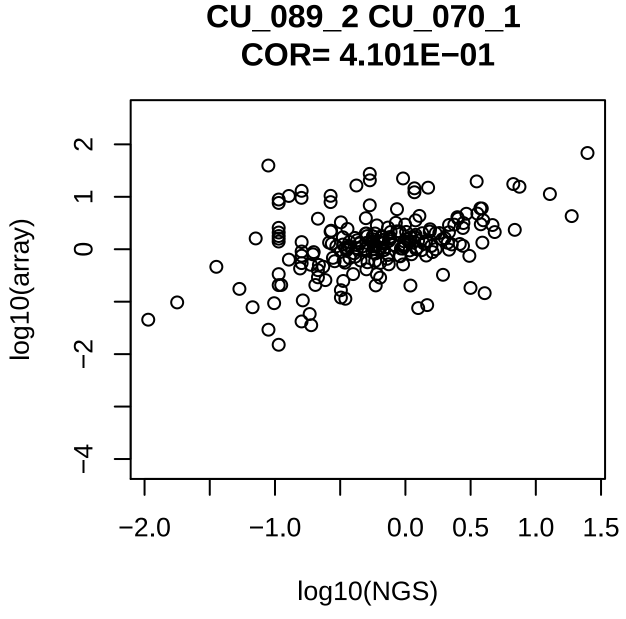

CU_089_2 CU_070_1
COR= 4.101E−01
log10(array)
log10(NGS)

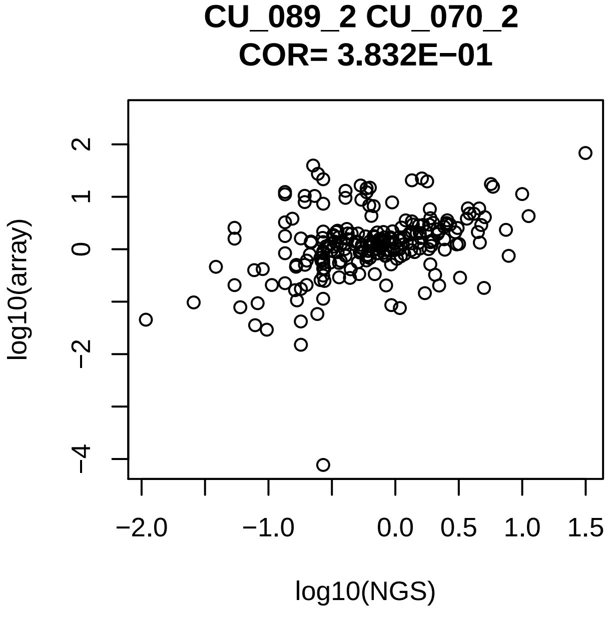

CU_089_2 CU_070_2
COR= 3.832E−01
log10(array)
log10(NGS)

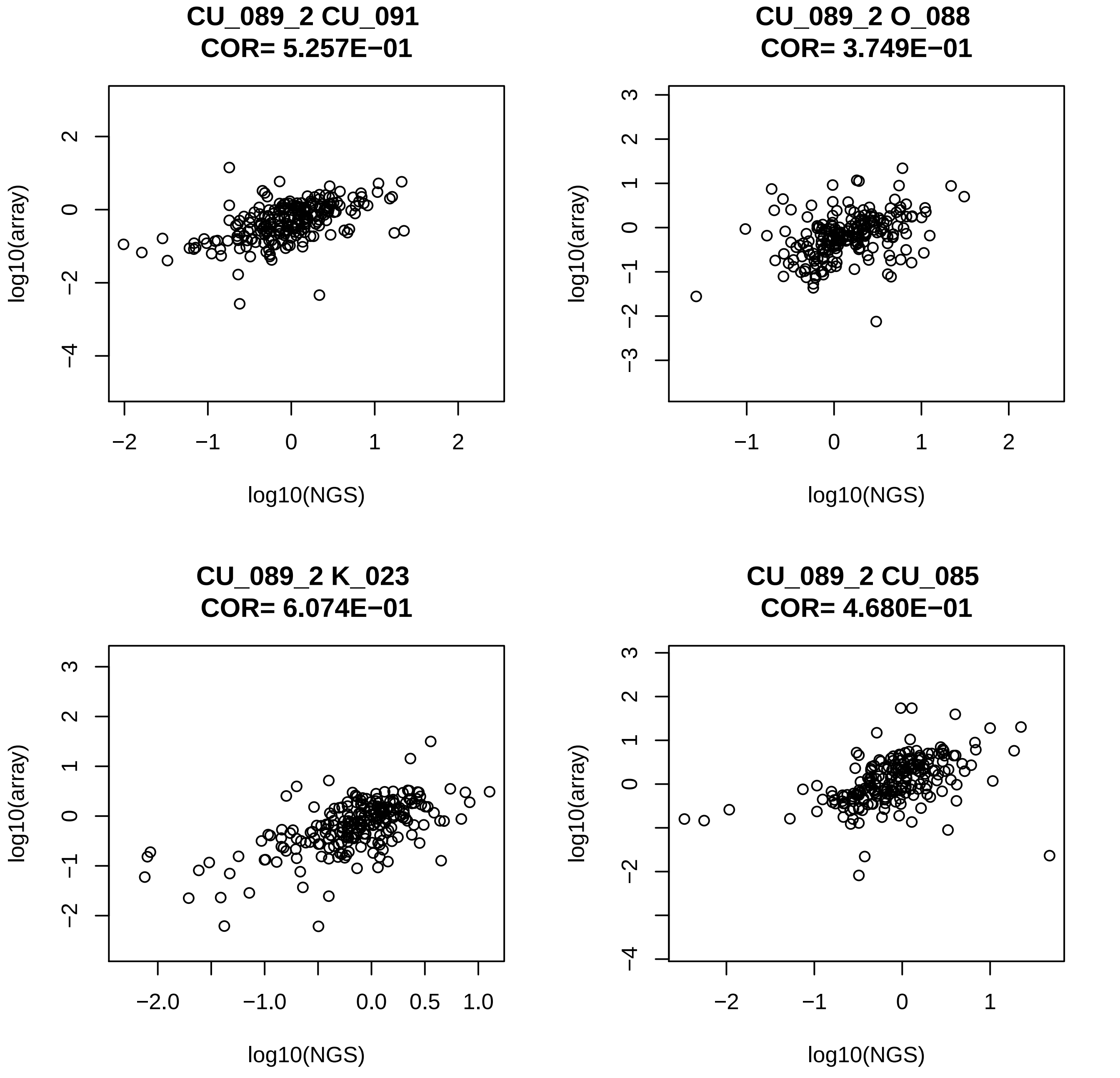
CU_089_2 CU_091
COR= 5.257E−01
CU_089_2 O_088
COR= 3.749E−01
CU_089_2 K_023
COR= 6.074E−01
CU_089_2 CU_085
COR= 4.680E−01
log10(array)
log10(NGS)

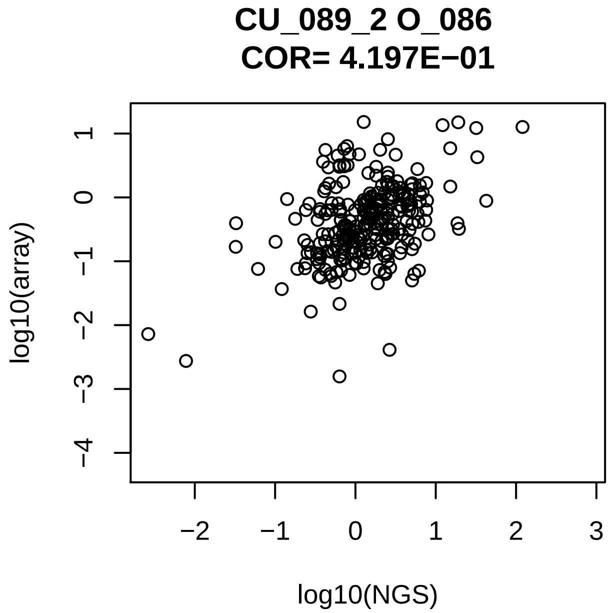
CU_089_2 O_086
COR= 4.197E−01
log10(array)
log10(NGS)
1
0
−1
−2
−3
−4
−2
−1
0
1
2
3
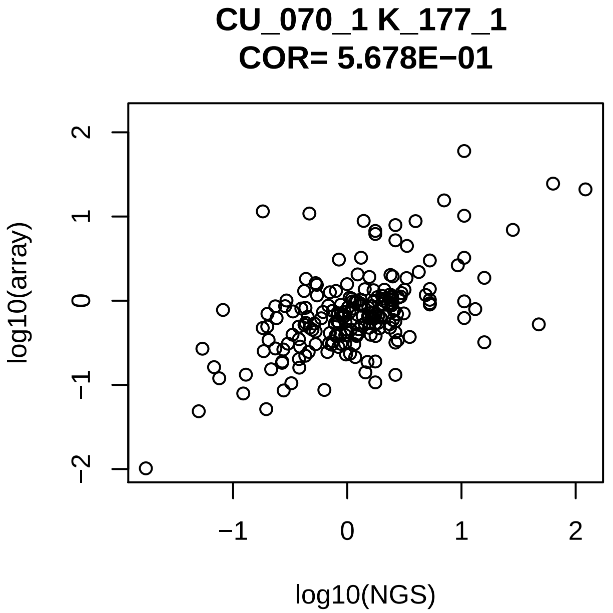
CU_070_1 K_177_1
COR= 5.678E−01
log10(array)
log10(NGS)
2
1
0
−1
−2
−1
0
1
2
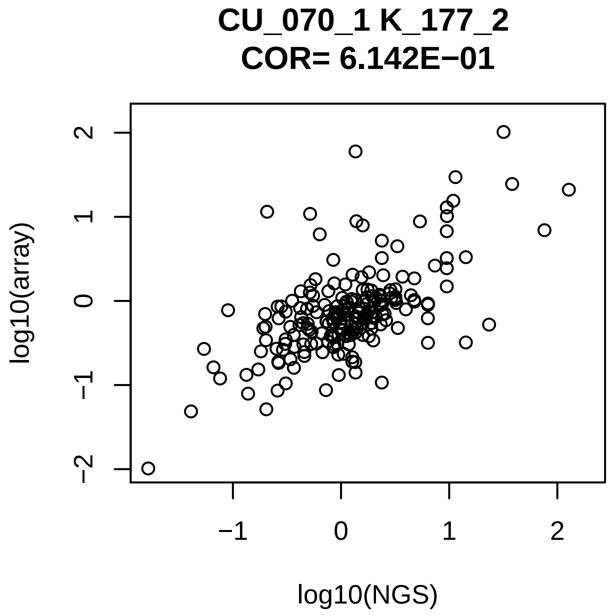
CU_070_1 K_177_2
COR= 6.142E−01
log10(array)
log10(NGS)
2
1
0
−1
−2
−1
0
1
2
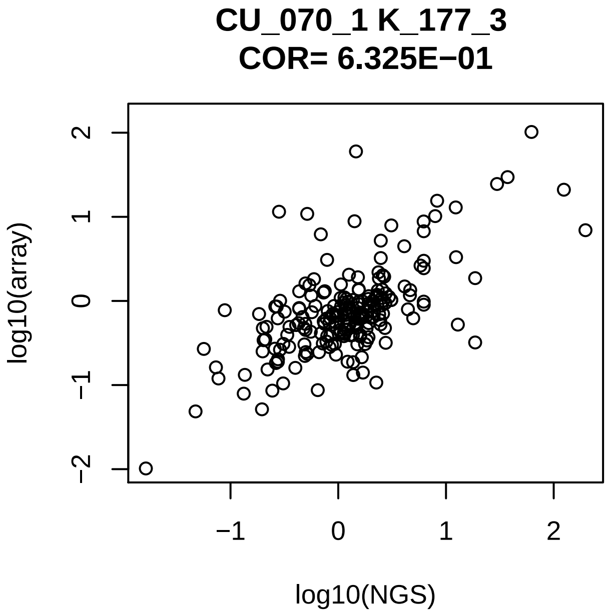
CU_070_1 K_177_3
COR= 6.325E−01
log10(array)
log10(NGS)
2
1
0
−1
−2
−1
0
1
2

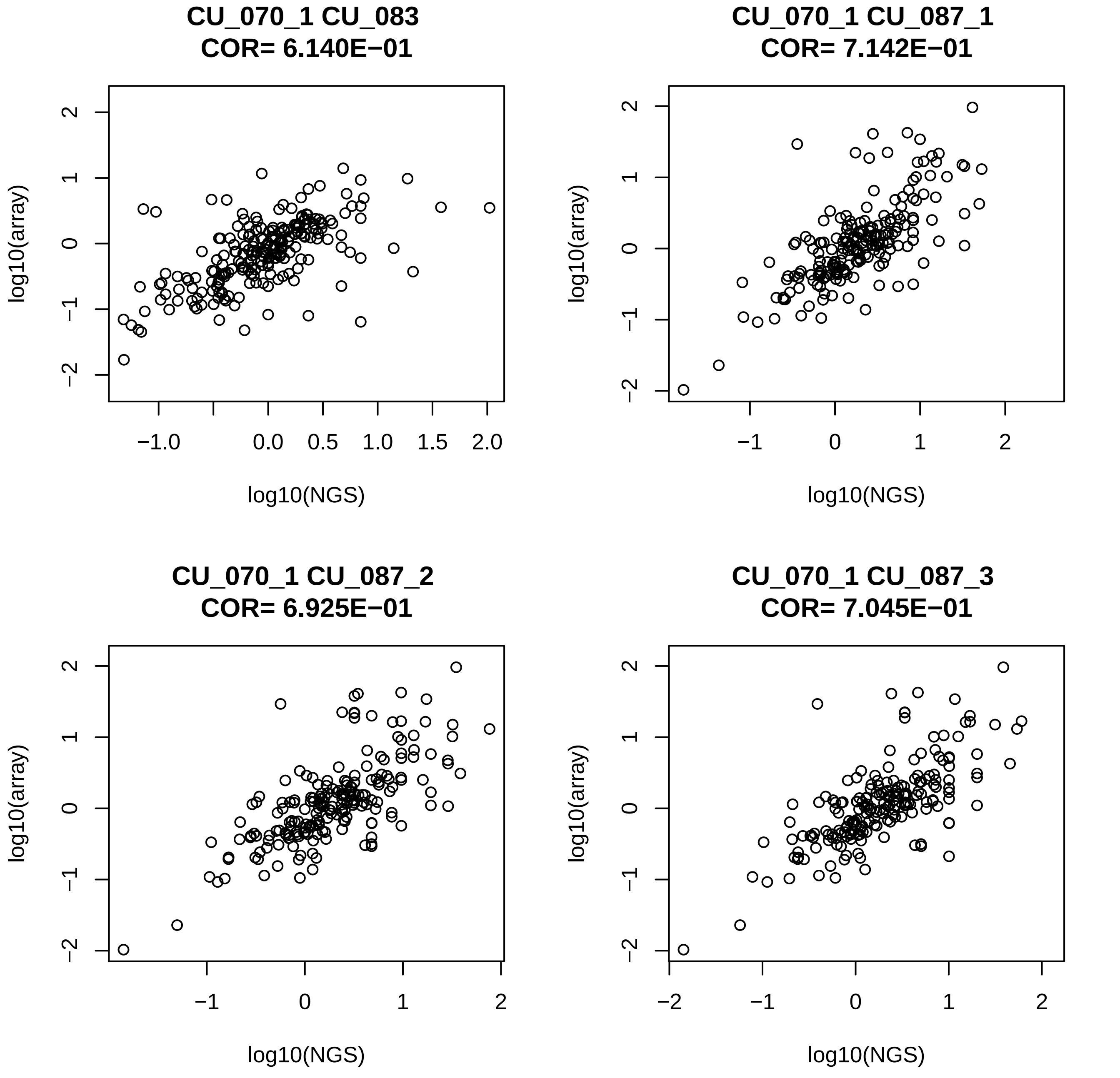
CU_070_1 CU_083
COR= 6.140E−01
log10(array)
log10(NGS)
CU_070_1 CU_087_1
COR= 7.142E−01
log10(array)
log10(NGS)
CU_070_1 CU_087_2
COR= 6.925E−01
log10(array)
log10(NGS)
CU_070_1 CU_087_3
COR= 7.045E−01
log10(array)
log10(NGS)

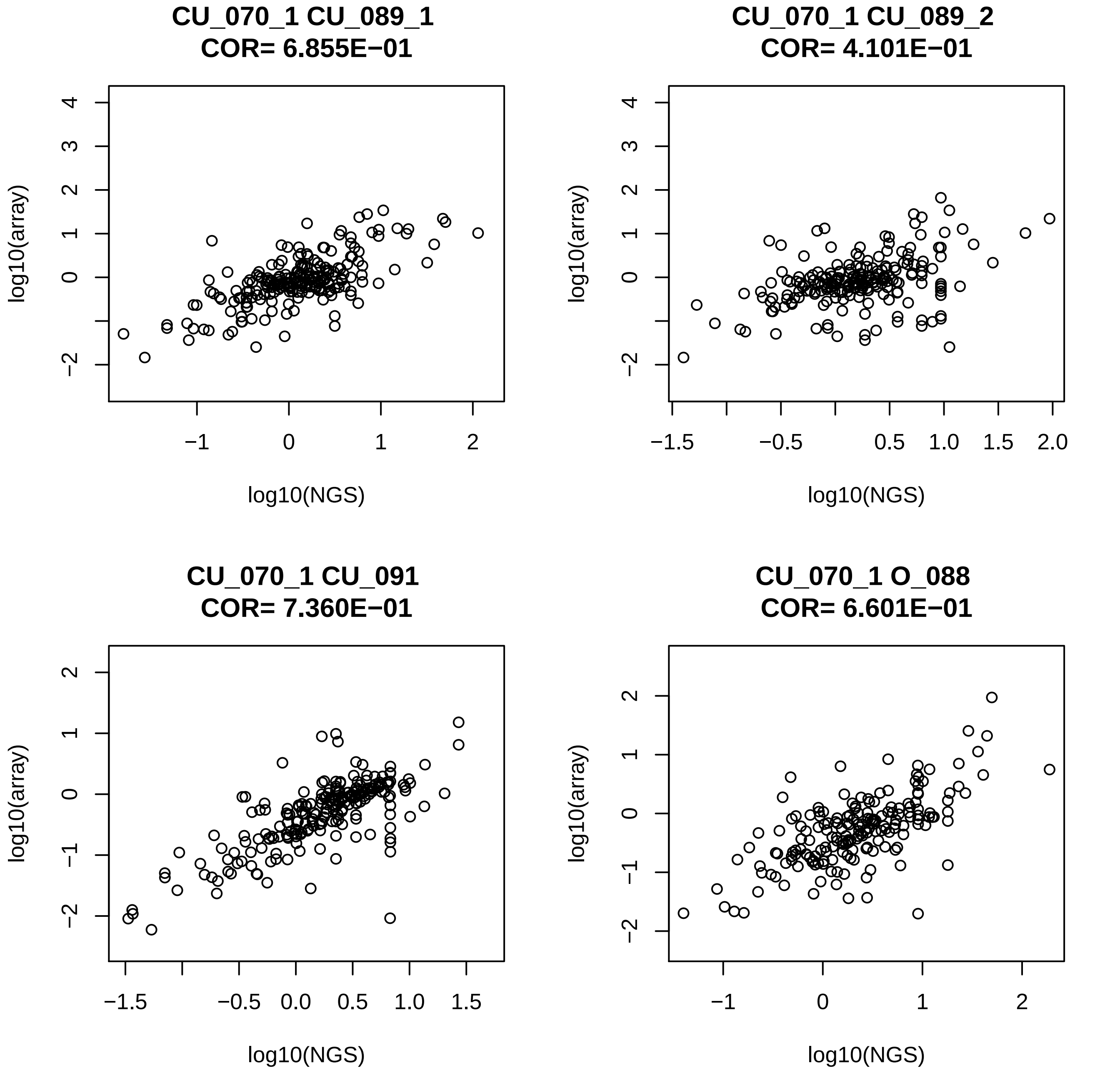
CU_070_1 CU_089_1
COR= 6.855E−01
CU_070_1 CU_089_2
COR= 4.101E−01
CU_070_1 CU_091
COR= 7.360E−01
CU_070_1 O_088
COR= 6.601E−01
log10(array)
log10(NGS)

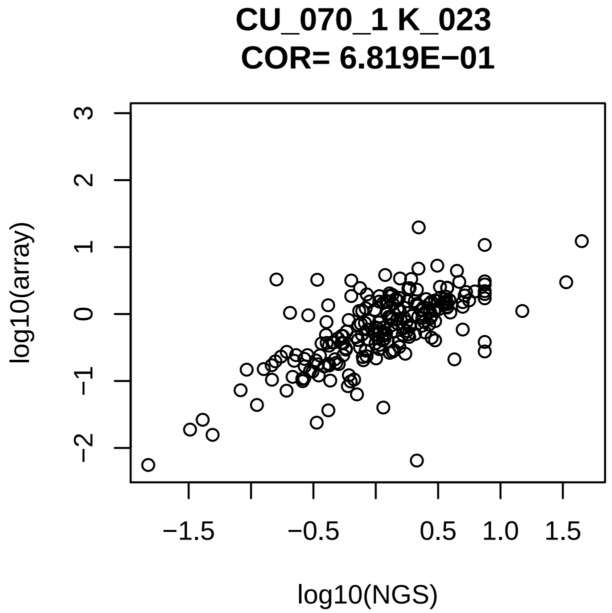
CU_070_1 K_023
COR= 6.819E−01
log10(array)
log10(NGS)

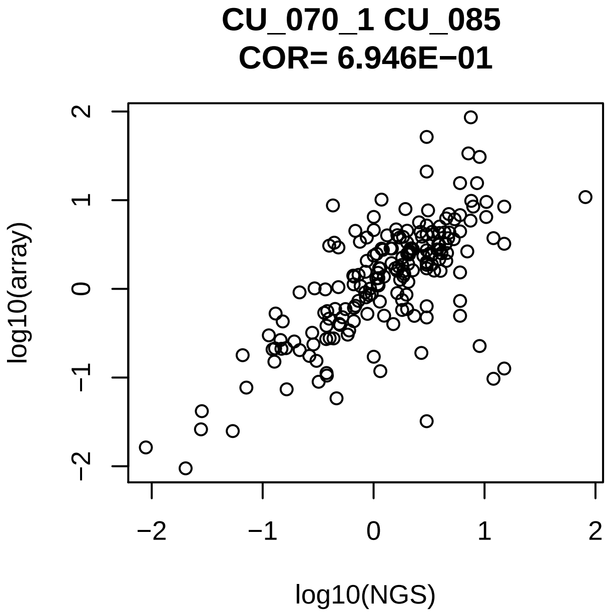
CU_070_1 CU_085
COR= 6.946E−01
log10(array)
log10(NGS)

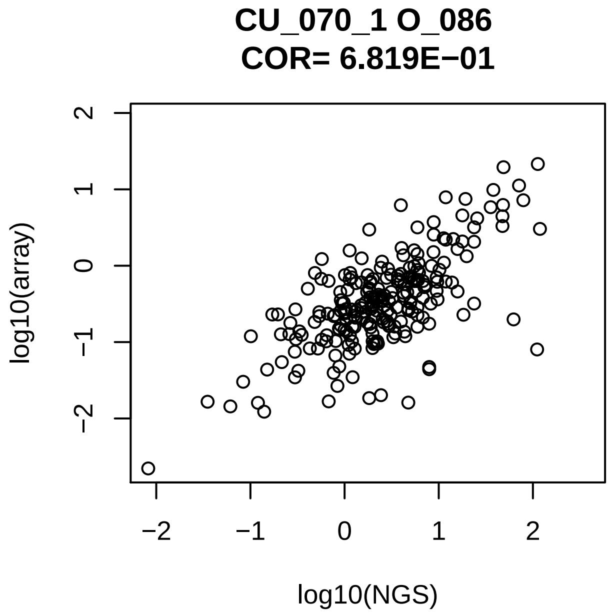
CU_070_1 O_086
COR= 6.819E−01
log10(array)
log10(NGS)

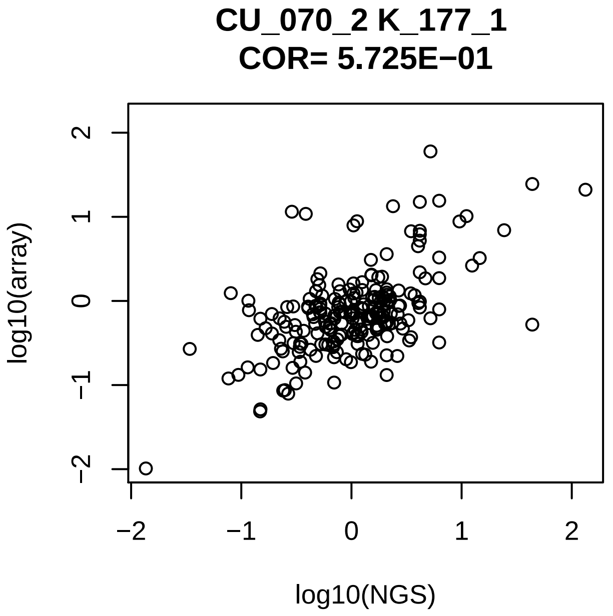
CU_070_2 K_177_1
COR= 5.725E−01
log10(array)
log10(NGS)

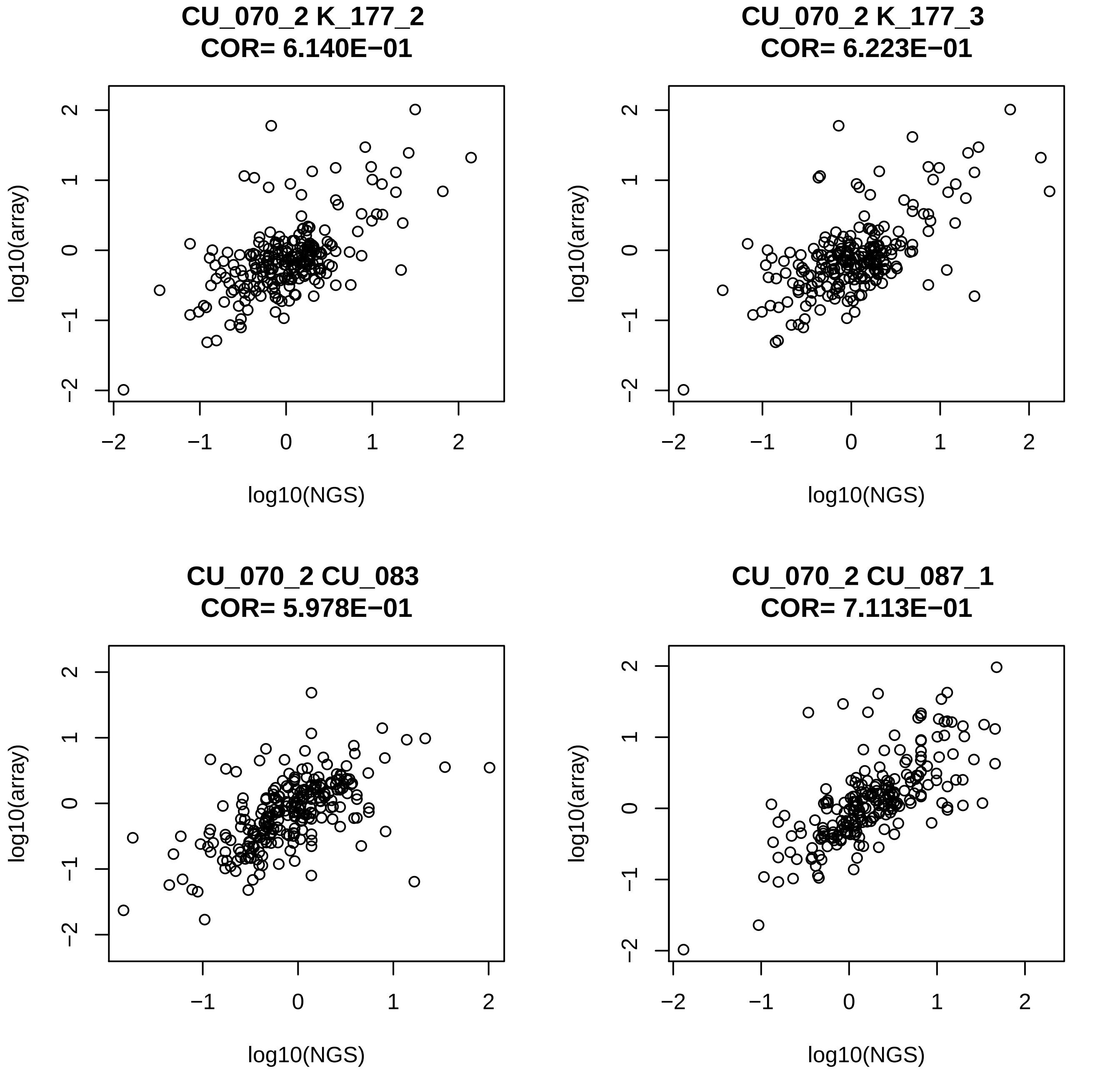
CU_070_2 K_177_2
COR= 6.140E−01
CU_070_2 K_177_3
COR= 6.223E−01
CU_070_2 CU_083
COR= 5.978E−01
CU_070_2 CU_087_1
COR= 7.113E−01
log10(array)
log10(NGS)
−2
−1
0
1
2

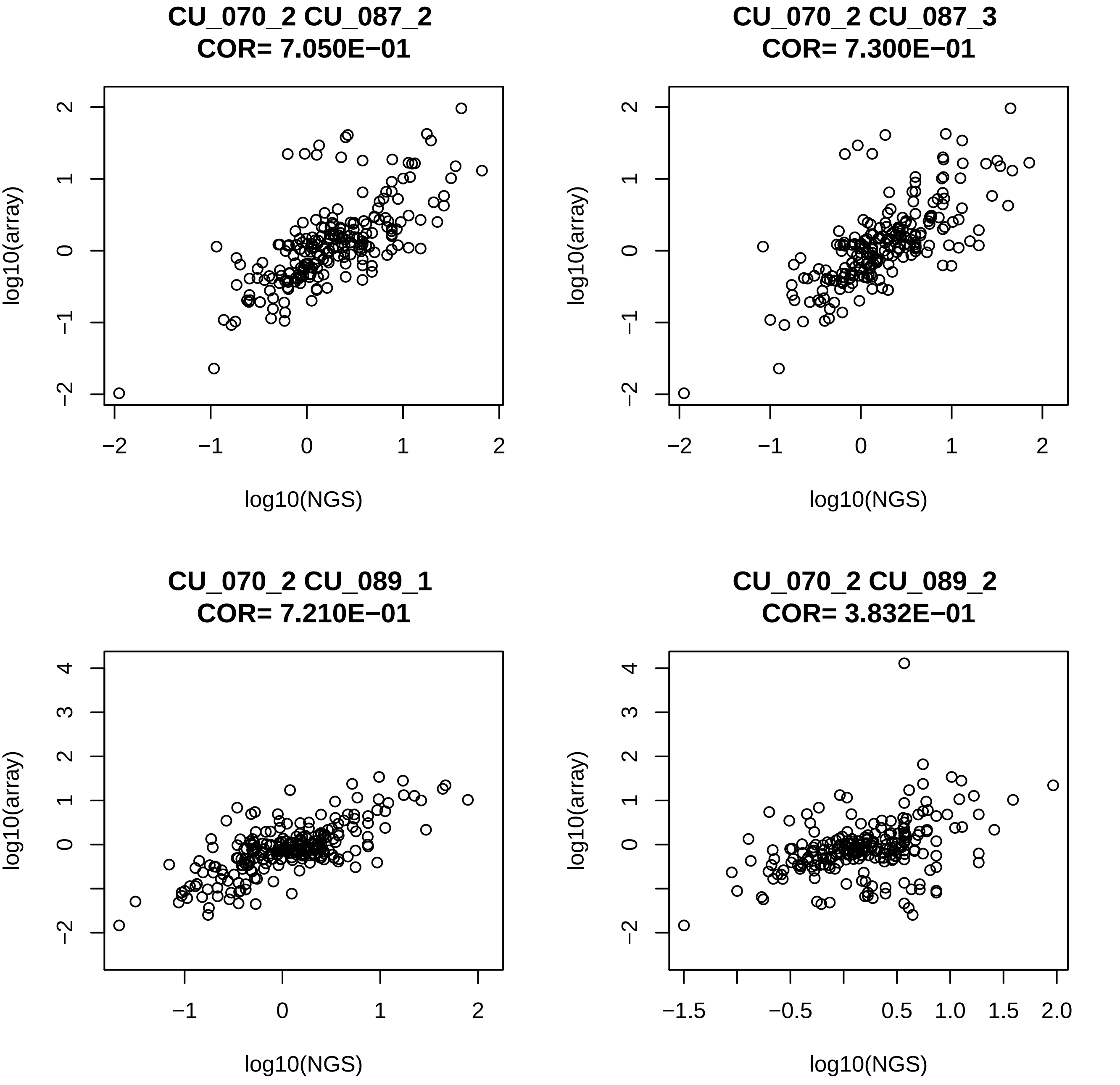
CU_070_2 CU_087_2
COR= 7.050E−01
log10(array)
log10(NGS)
CU_070_2 CU_087_3
COR= 7.300E−01
log10(array)
log10(NGS)
CU_070_2 CU_089_1
COR= 7.210E−01
log10(array)
log10(NGS)
CU_070_2 CU_089_2
COR= 3.832E−01
log10(array)
log10(NGS)

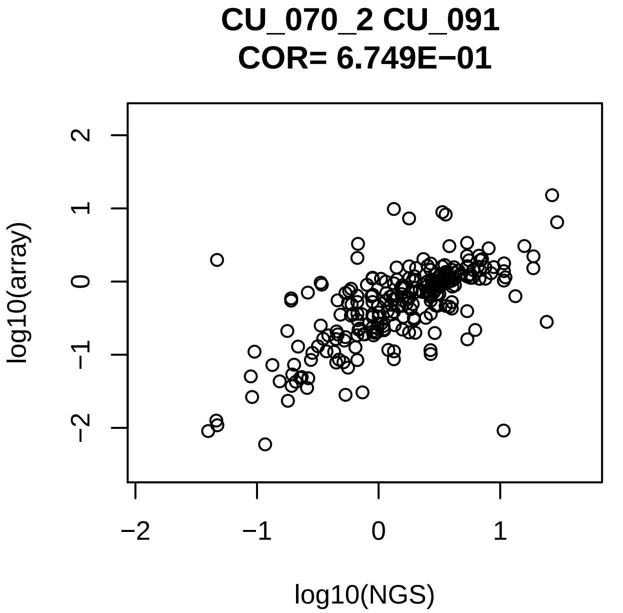
CU_070_2 CU_091
COR= 6.749E−01
log10(array)
log10(NGS)
2
1
0
−1
−2
−2
−1
0
1

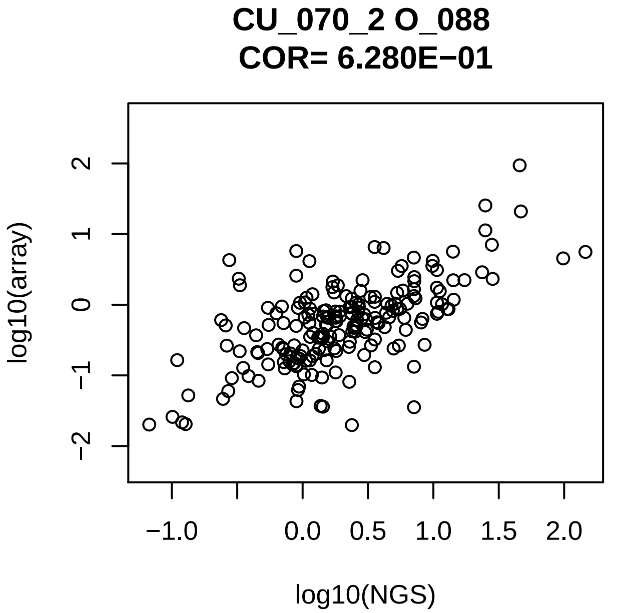
CU_070_2 O_088
COR= 6.280E−01
log10(array)
log10(NGS)
2
1
0
−1
−2
−1.0
0.0
0.5
1.0
1.5
2.0

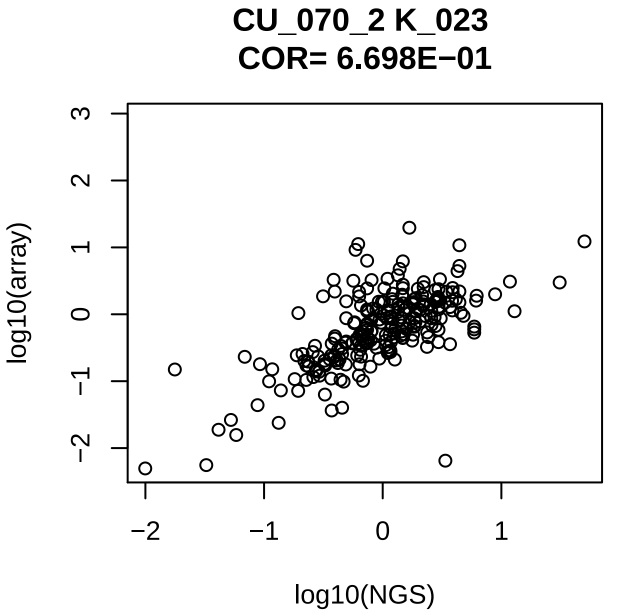
CU_070_2 K_023
COR= 6.698E−01
log10(array)
log10(NGS)
3
2
1
0
−1
−2
−2
−1
0
1

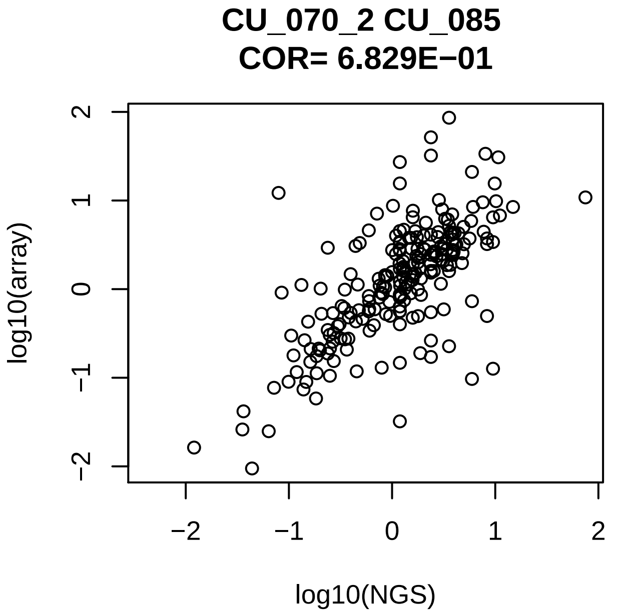
CU_070_2 CU_085
COR= 6.829E−01
log10(array)
log10(NGS)
2
1
0
−1
−2
−2
−1
0
1
2

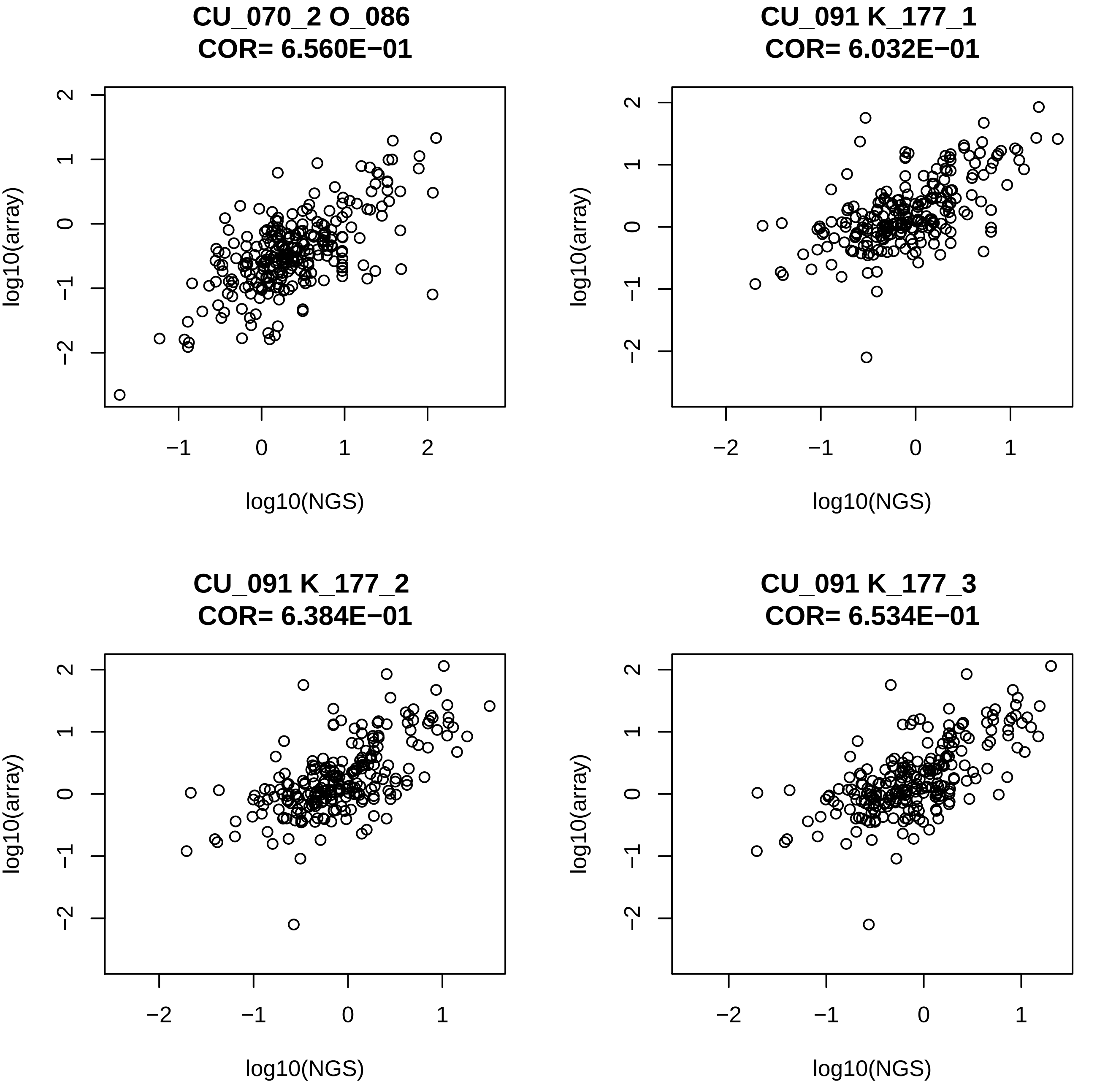
CU_070_2 O_086
COR= 6.560E−01
log10(array)
log10(NGS)
CU_091 K_177_1
COR= 6.032E−01
log10(array)
log10(NGS)
CU_091 K_177_2
COR= 6.384E−01
log10(array)
log10(NGS)
CU_091 K_177_3
COR= 6.534E−01
log10(array)
log10(NGS)

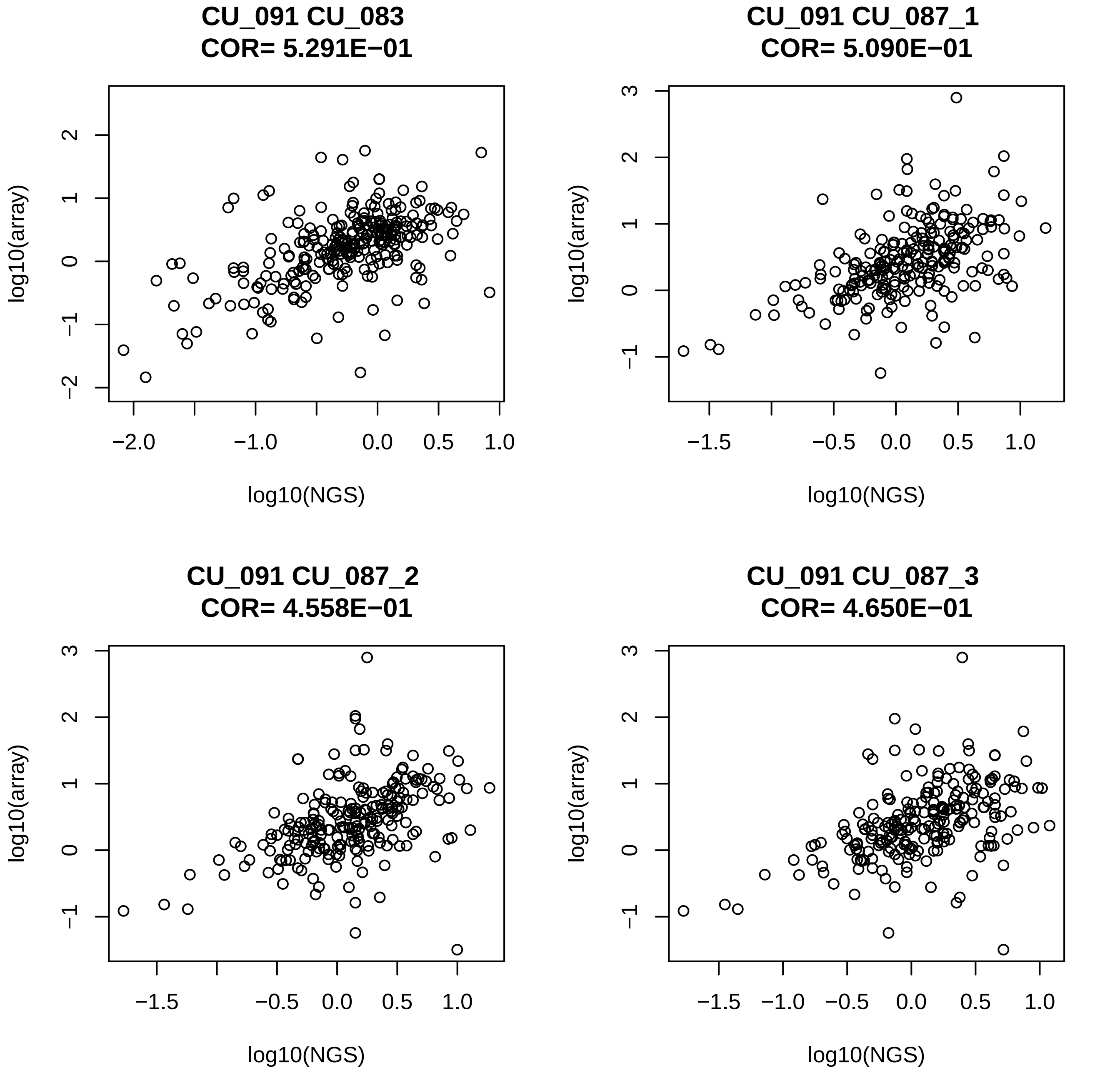
CU_091 CU_083
COR= 5.291E−01
CU_091 CU_087_1
COR= 5.090E−01
CU_091 CU_087_2
COR= 4.558E−01
CU_091 CU_087_3
COR= 4.650E−01
log10(array)
log10(NGS)

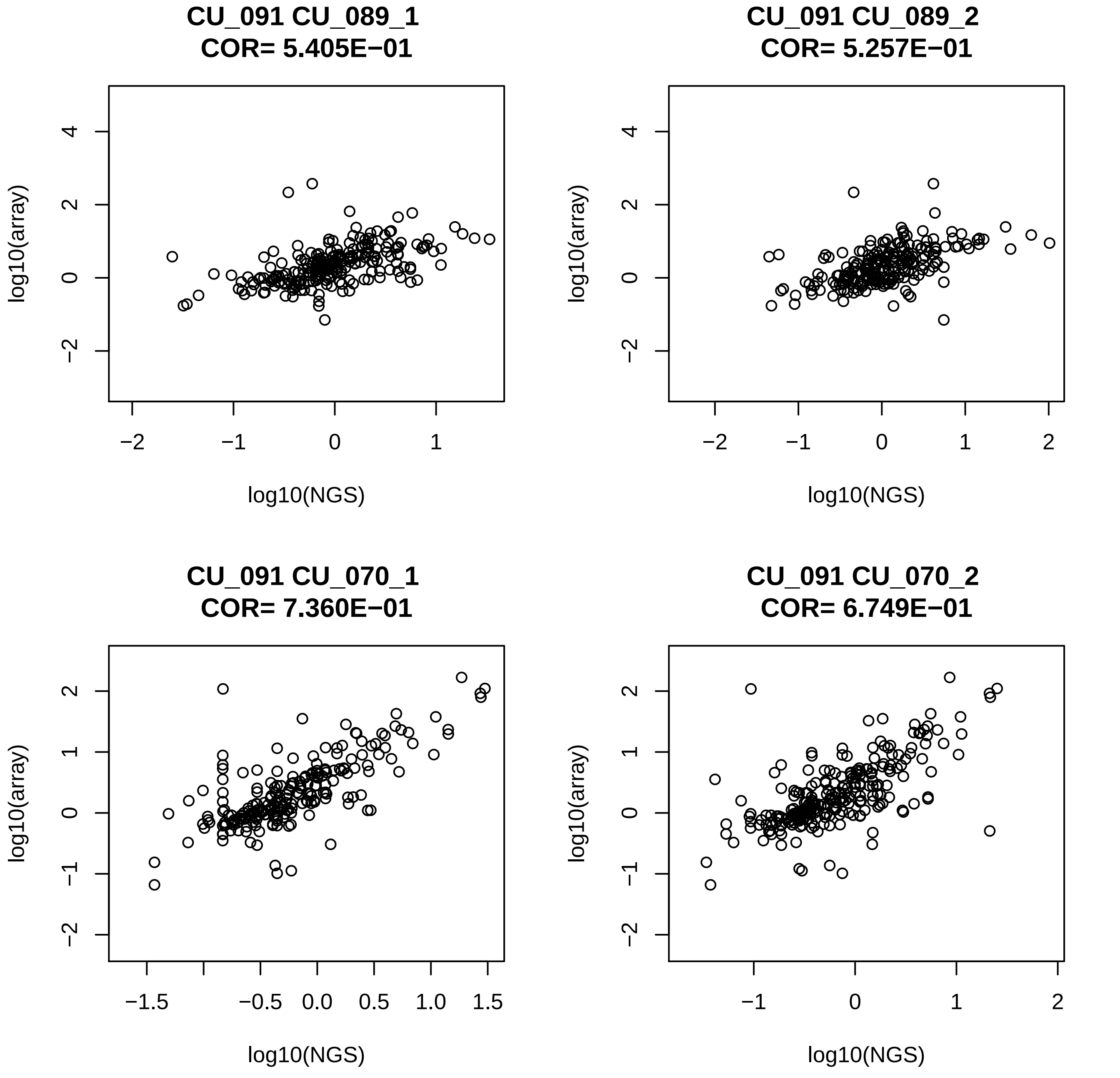

CU_091 CU_089_1
COR= 5.405E−01
log10(array)
log10(NGS)
CU_091 CU_089_2
COR= 5.257E−01
log10(array)
log10(NGS)
CU_091 CU_070_1
COR= 7.360E−01
log10(array)
log10(NGS)
CU_091 CU_070_2
COR= 6.749E−01
log10(array)
log10(NGS)

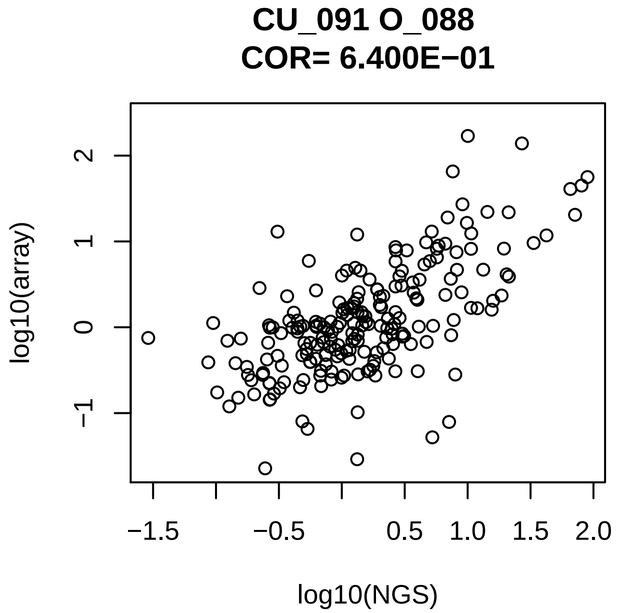
CU_091 O_088
COR= 6.400E−01
log10(array)
log10(NGS)
2
1
0
−1
−1.5
−0.5
0.5
1.0
1.5
2.0

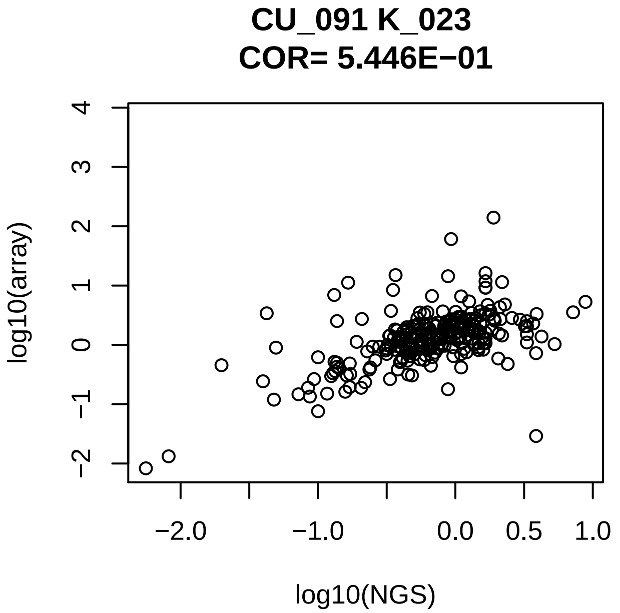
CU_091 K_023
COR= 5.446E−01
log10(array)
log10(NGS)
4
3
2
1
0
−1
−2
−2.0
−1.0
0.0
0.5
1.0

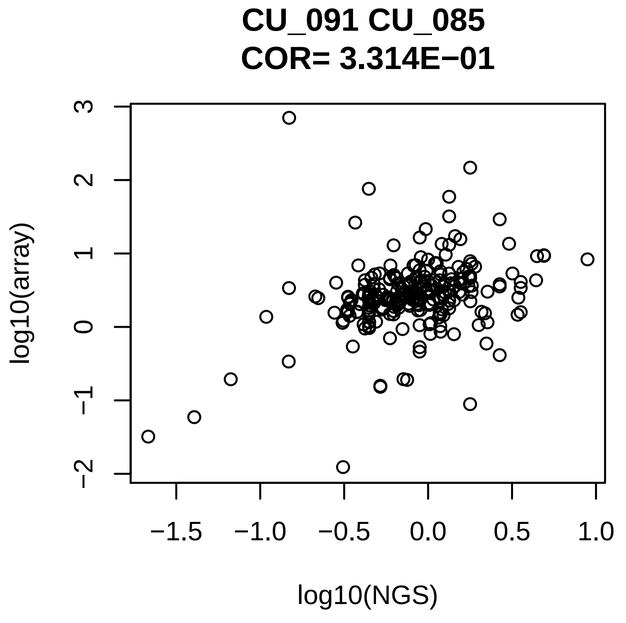
CU_091 CU_085
COR= 3.314E−01
log10(array)
log10(NGS)
3
2
1
0
−1
−2
−1.5
−1.0
−0.5
0.0
0.5
1.0

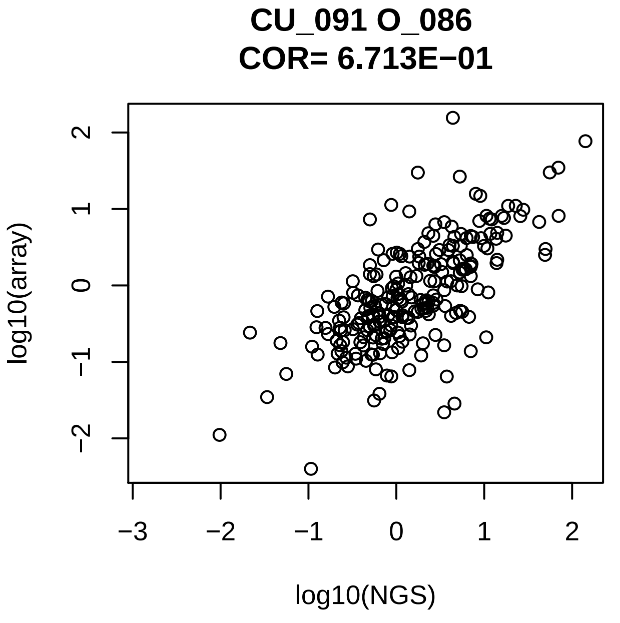
CU_091 O_086
COR= 6.713E−01
log10(array)
log10(NGS)
2
1
0
−1
−2
−3
−2
−1
0
1
2

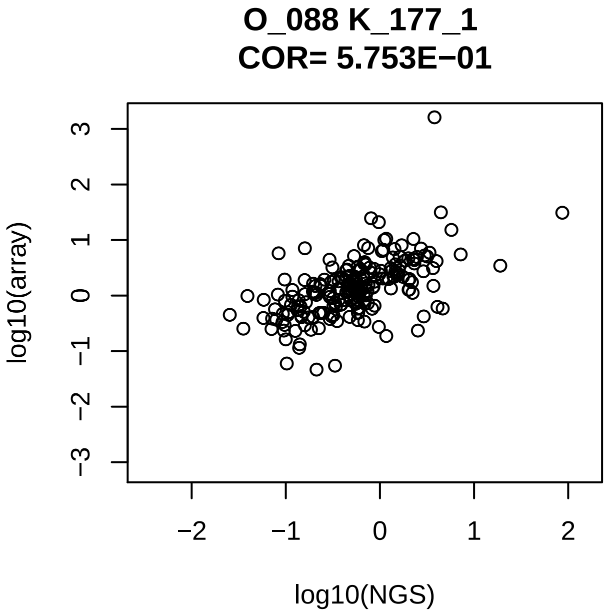
O_088 K_177_1
COR= 5.753E−01
log10(array)
log10(NGS)

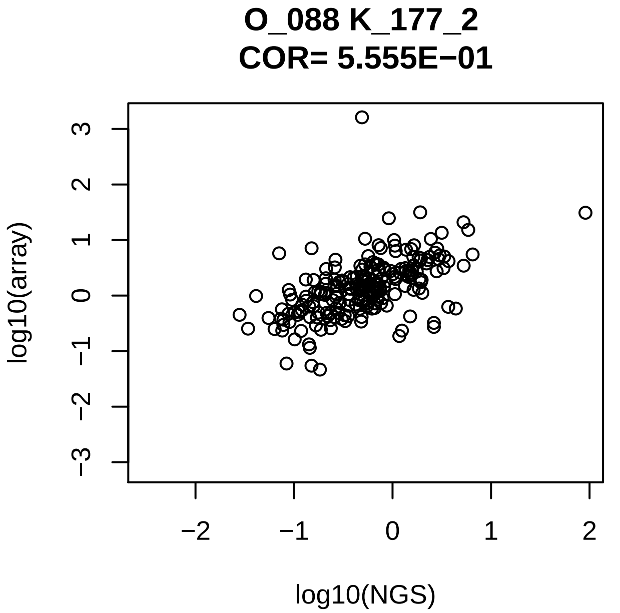
O_088 K_177_2
COR= 5.555E−01
log10(array)
log10(NGS)

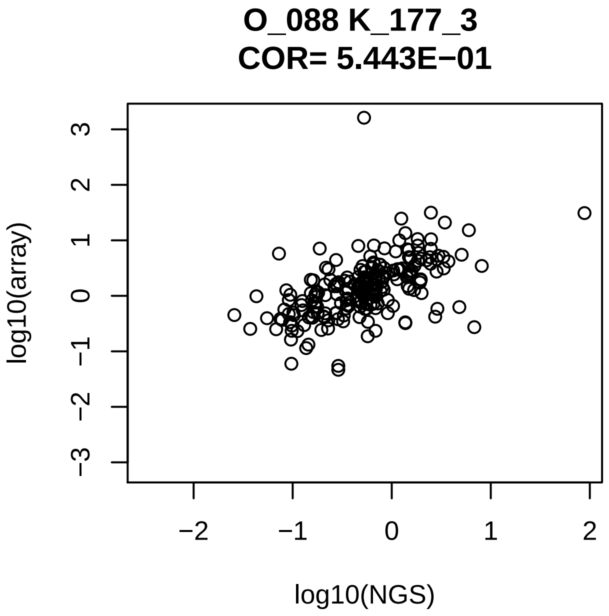
O_088 K_177_3
COR= 5.443E−01
log10(array)
log10(NGS)

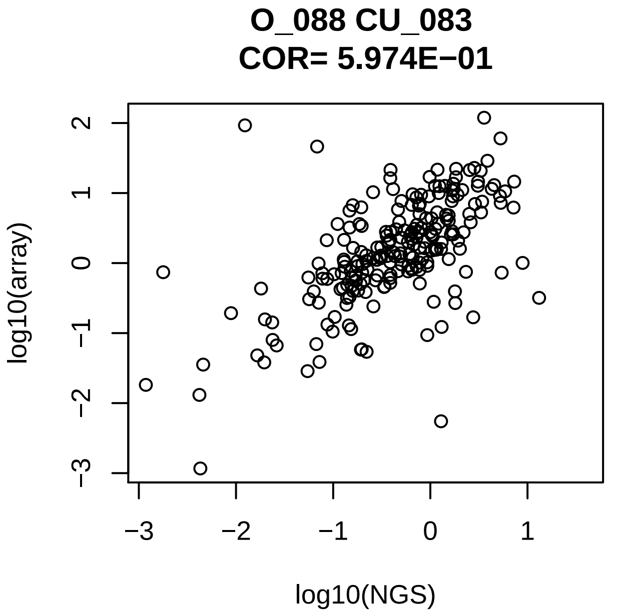
O_088 CU_083
COR= 5.974E−01
log10(array)
log10(NGS)

O_088 CU_087_1
COR= 4.963E−01

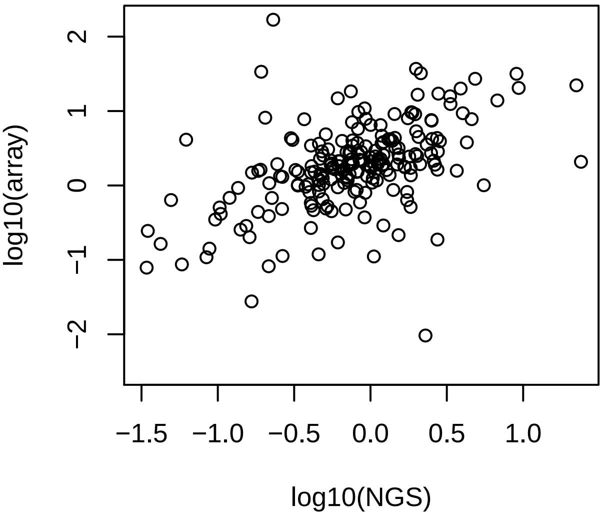

O_088 CU_087_2
COR= 4.763E−01

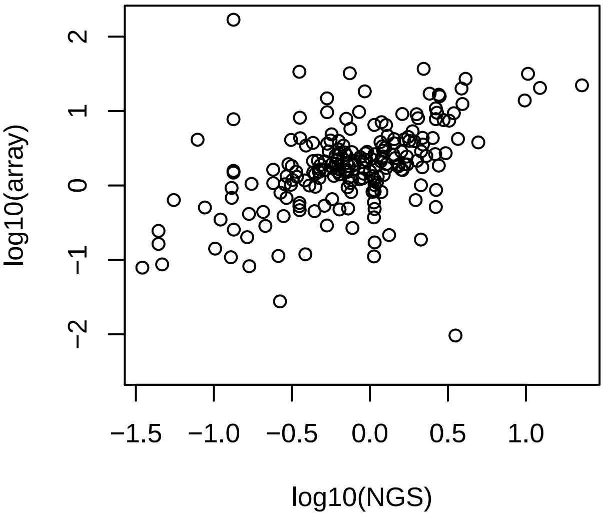

O_088 CU_087_3
COR= 5.026E−01

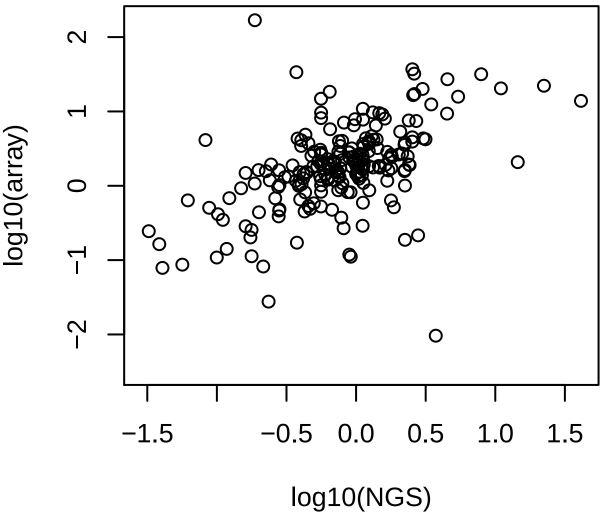

O_088 CU_089_1
COR= 5.082E−01

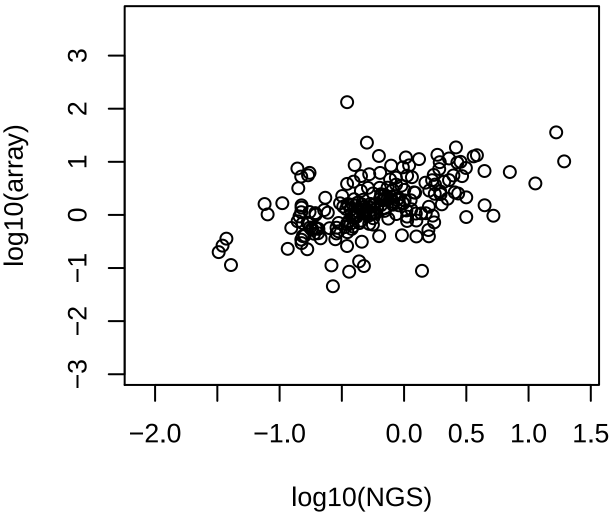

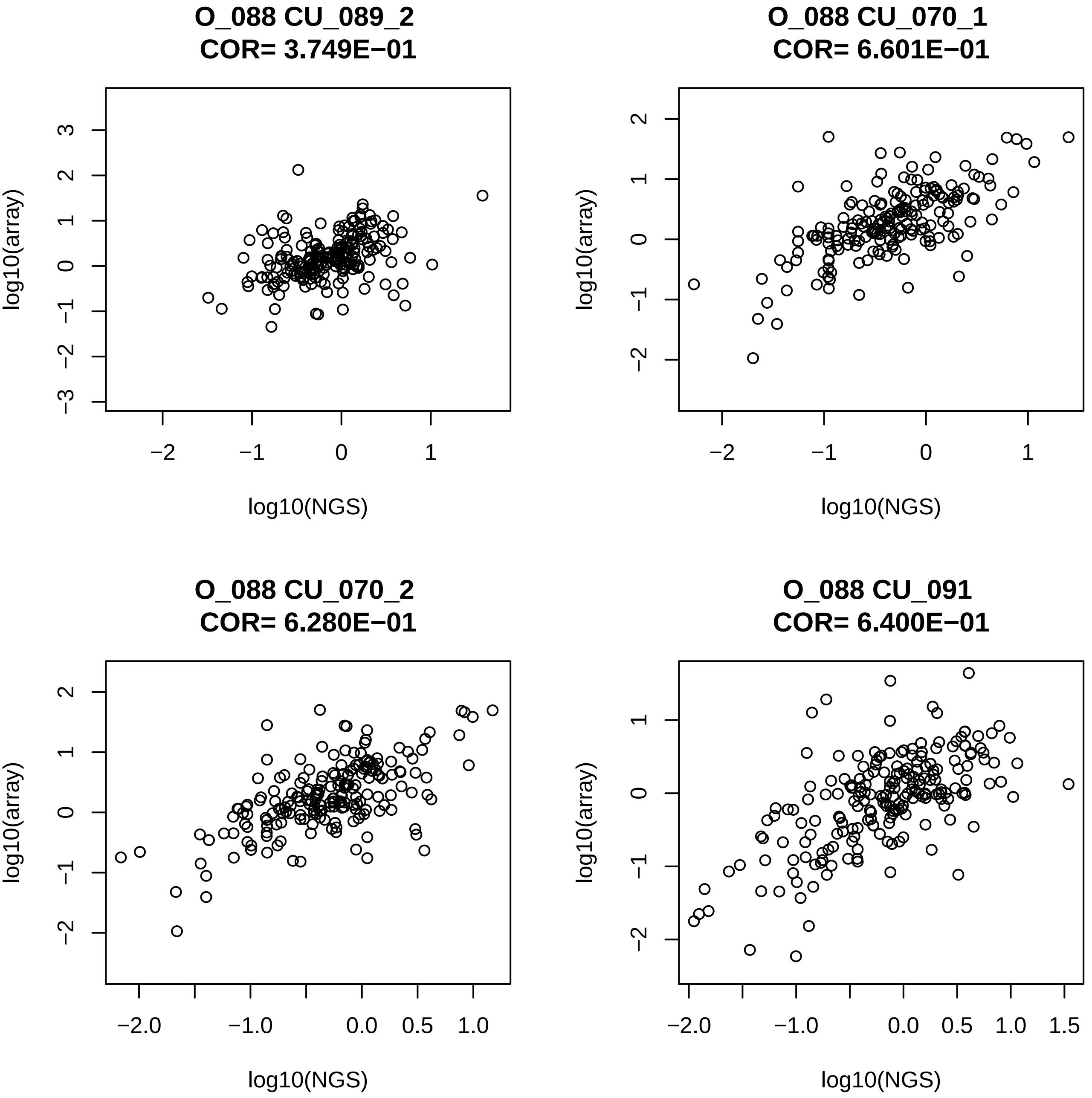
O_088 CU_089_2
COR= 3.749E−01
O_088 CU_070_1
COR= 6.601E−01
O_088 CU_070_2
COR= 6.280E−01
O_088 CU_091
COR= 6.400E−01
log10(array)
log10(NGS)

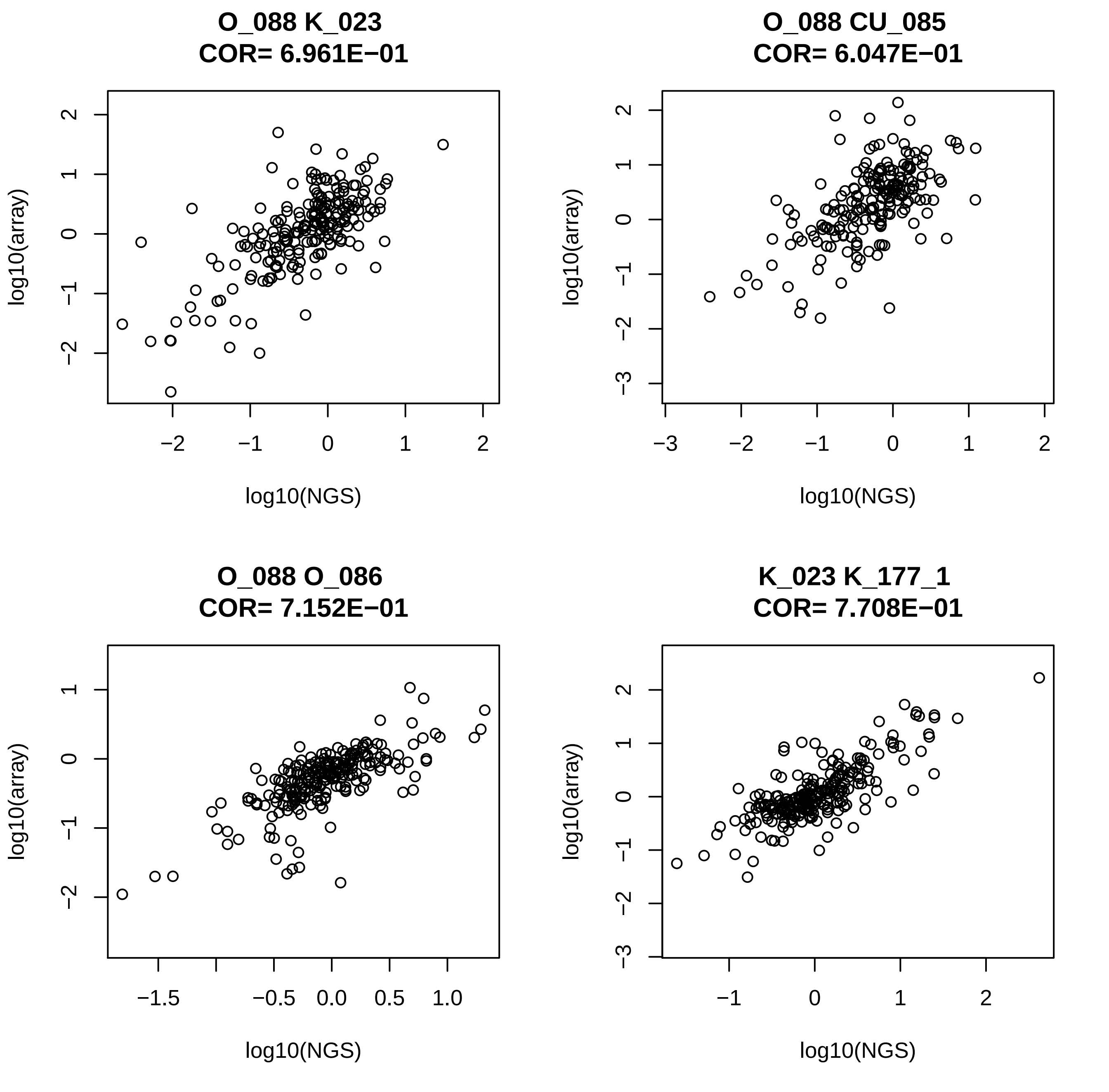

O_088 K_023
COR= 6.961E−01
log10(array)
log10(NGS)
O_088 CU_085
COR= 6.047E−01
log10(array)
log10(NGS)
O_088 O_086
COR= 7.152E−01
log10(array)
log10(NGS)
K_023 K_177_1
COR= 7.708E−01
log10(array)
log10(NGS)

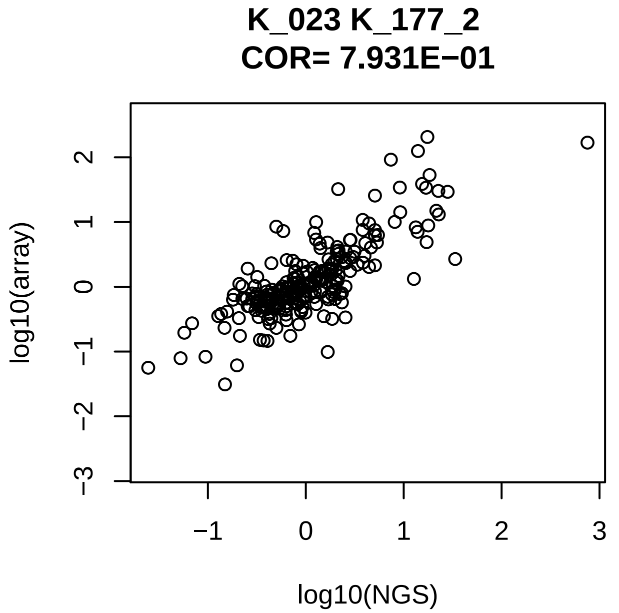
K_023 K_177_2
COR= 7.931E−01
log10(array)
log10(NGS)
−3 −2 −1 0 1 2
−1 0 1 2 3

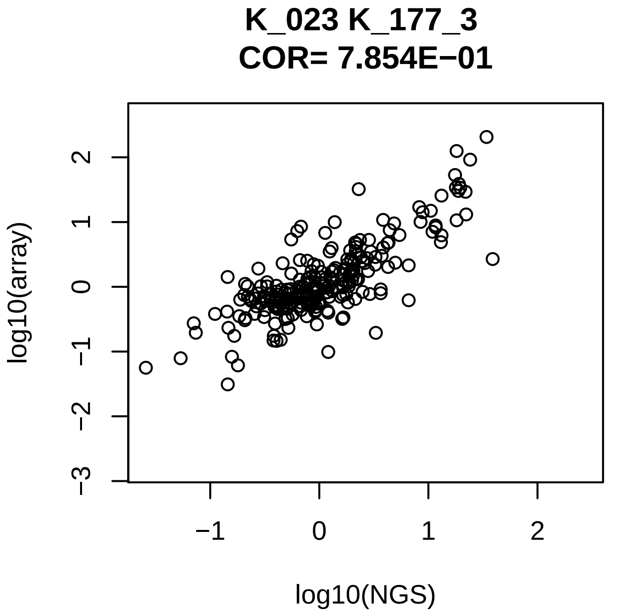
K_023 K_177_3
COR= 7.854E−01
log10(array)
log10(NGS)
−3 −2 −1 0 1 2
−1 0 1 2

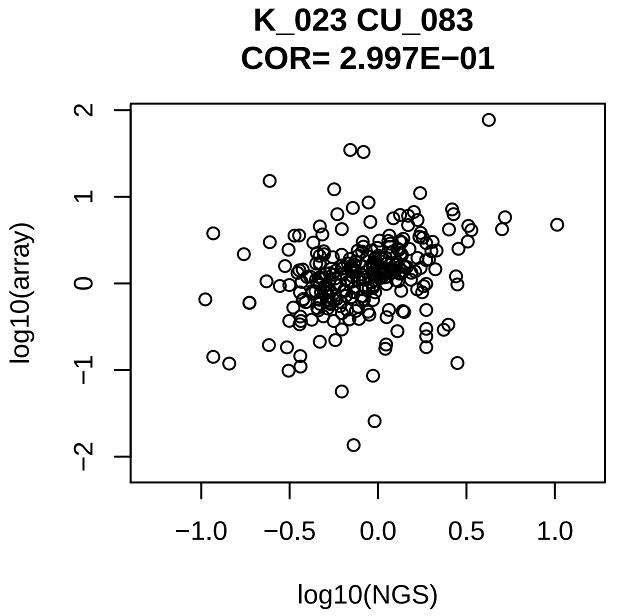
K_023 CU_083
COR= 2.997E−01
log10(array)
log10(NGS)
−2 −1 0 1 2
−1.0 −0.5 0.0 0.5 1.0

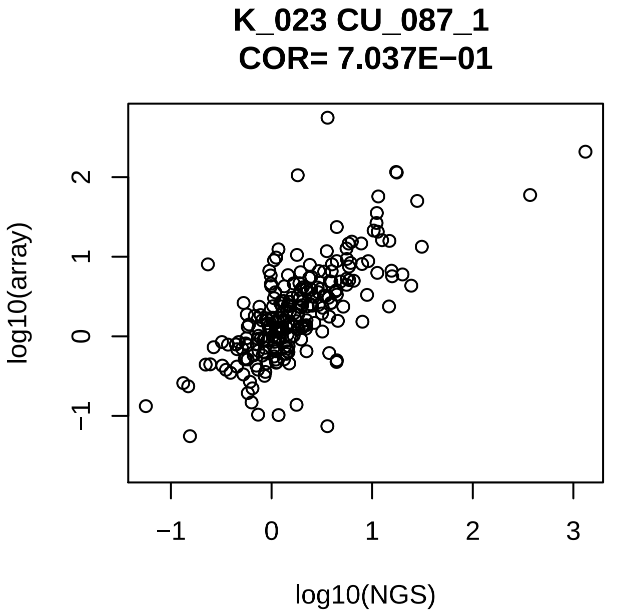
K_023 CU_087_1
COR= 7.037E−01
log10(array)
log10(NGS)
−1 0 1 2
−1 0 1 2 3

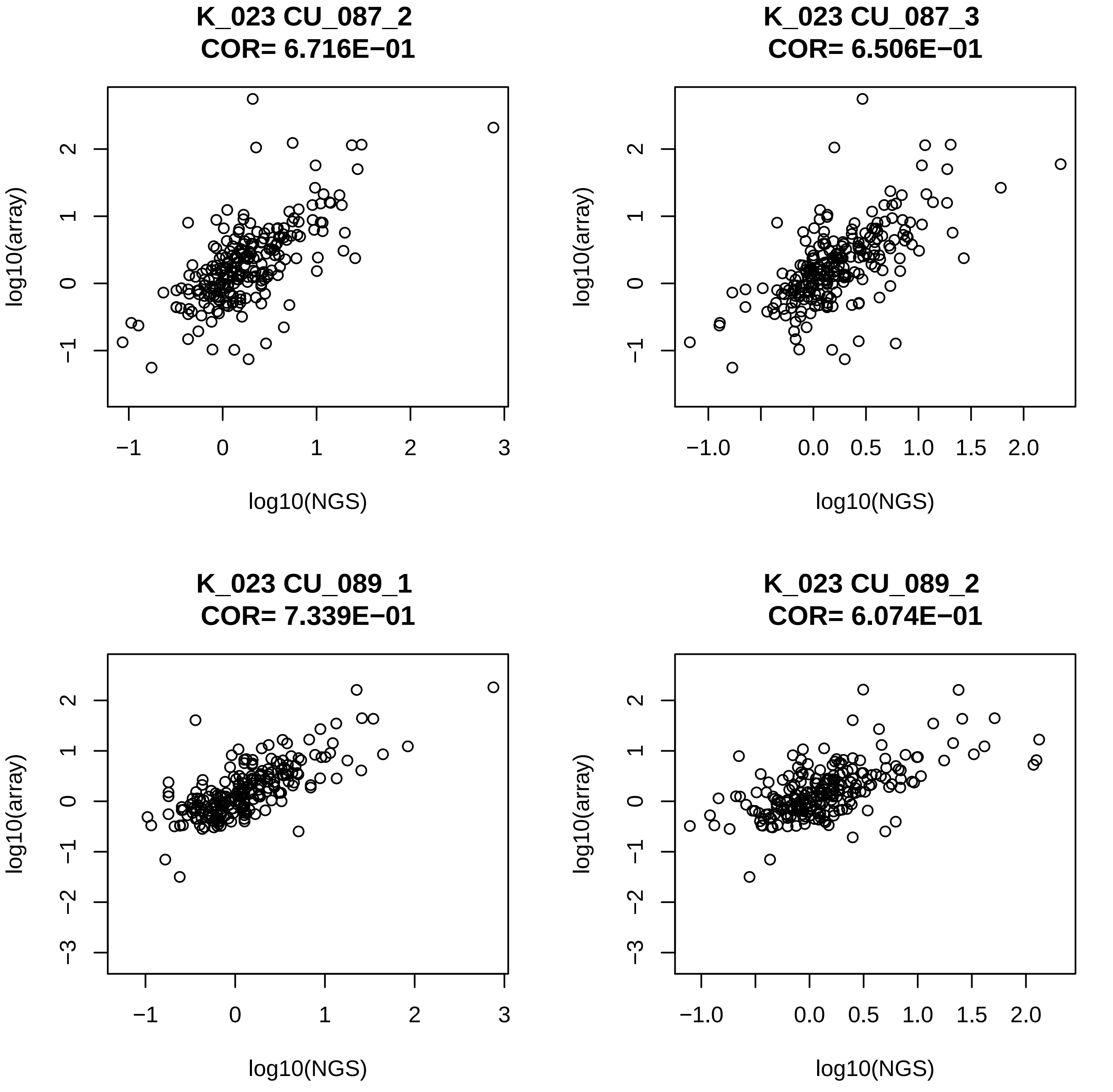
K_023 CU_087_2
COR= 6.716E−01
log10(array)
log10(NGS)
K_023 CU_087_3
COR= 6.506E−01
log10(array)
log10(NGS)
K_023 CU_089_1
COR= 7.339E−01
log10(array)
log10(NGS)
K_023 CU_089_2
COR= 6.074E−01
log10(array)
log10(NGS)

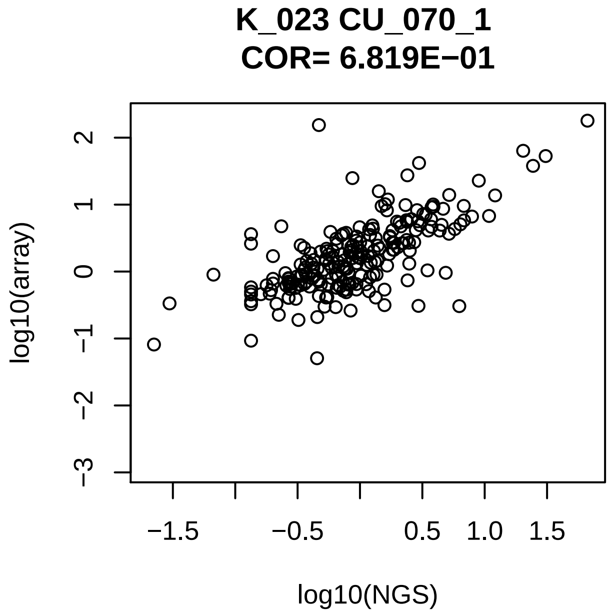
K_023 CU_070_1
COR= 6.819E−01
log10(array)
log10(NGS)
2
1
0
−1
−2
−3
−1.5
−0.5
0.5
1.0
1.5

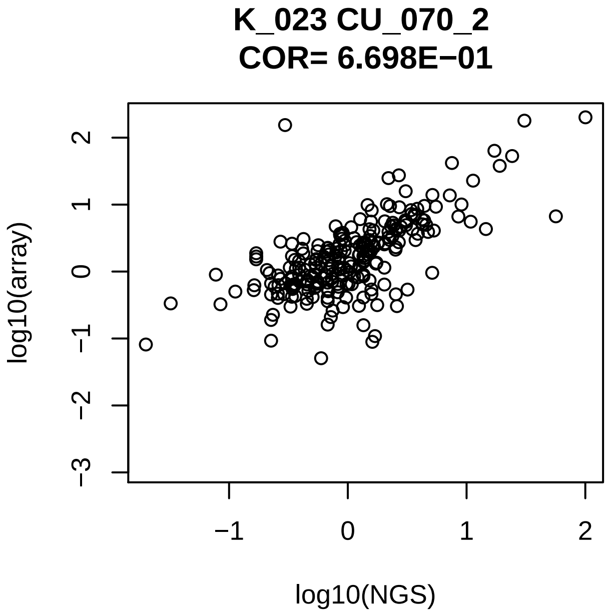
K_023 CU_070_2
COR= 6.698E−01
log10(array)
log10(NGS)
2
1
0
−1
−2
−3
−1
0
1
2

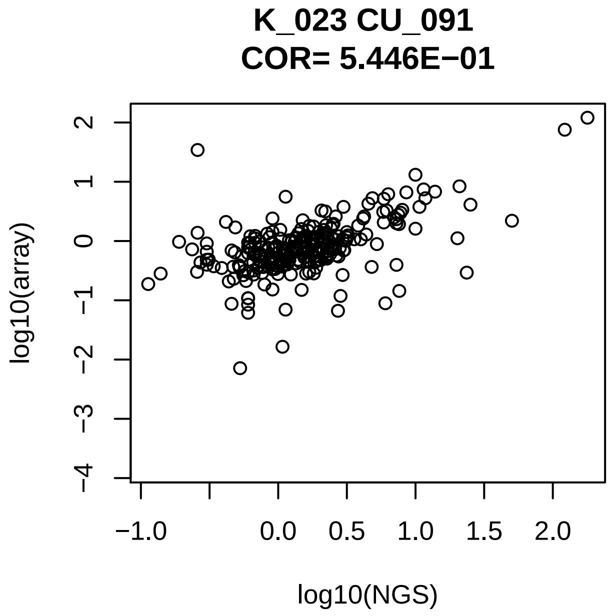
K_023 CU_091
COR= 5.446E−01
log10(array)
log10(NGS)
2
1
0
−1
−2
−3
−4
−1.0
0.0
0.5
1.0
1.5
2.0

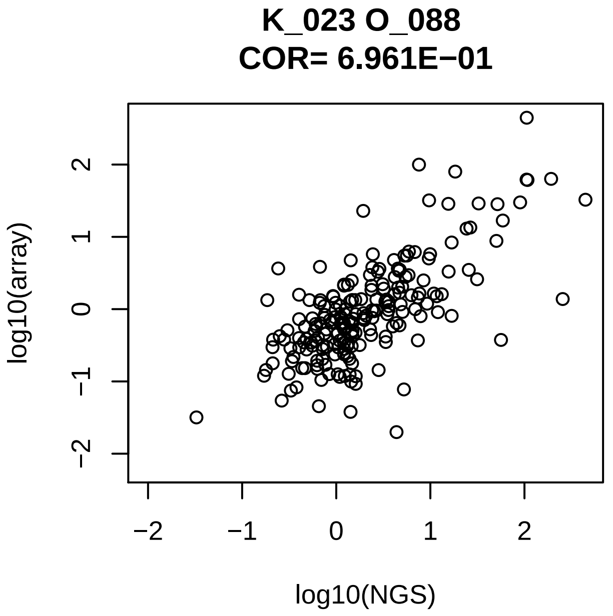
K_023 O_088
COR= 6.961E−01
log10(array)
log10(NGS)
2
1
0
−1
−2
−2
−1
0
1
2

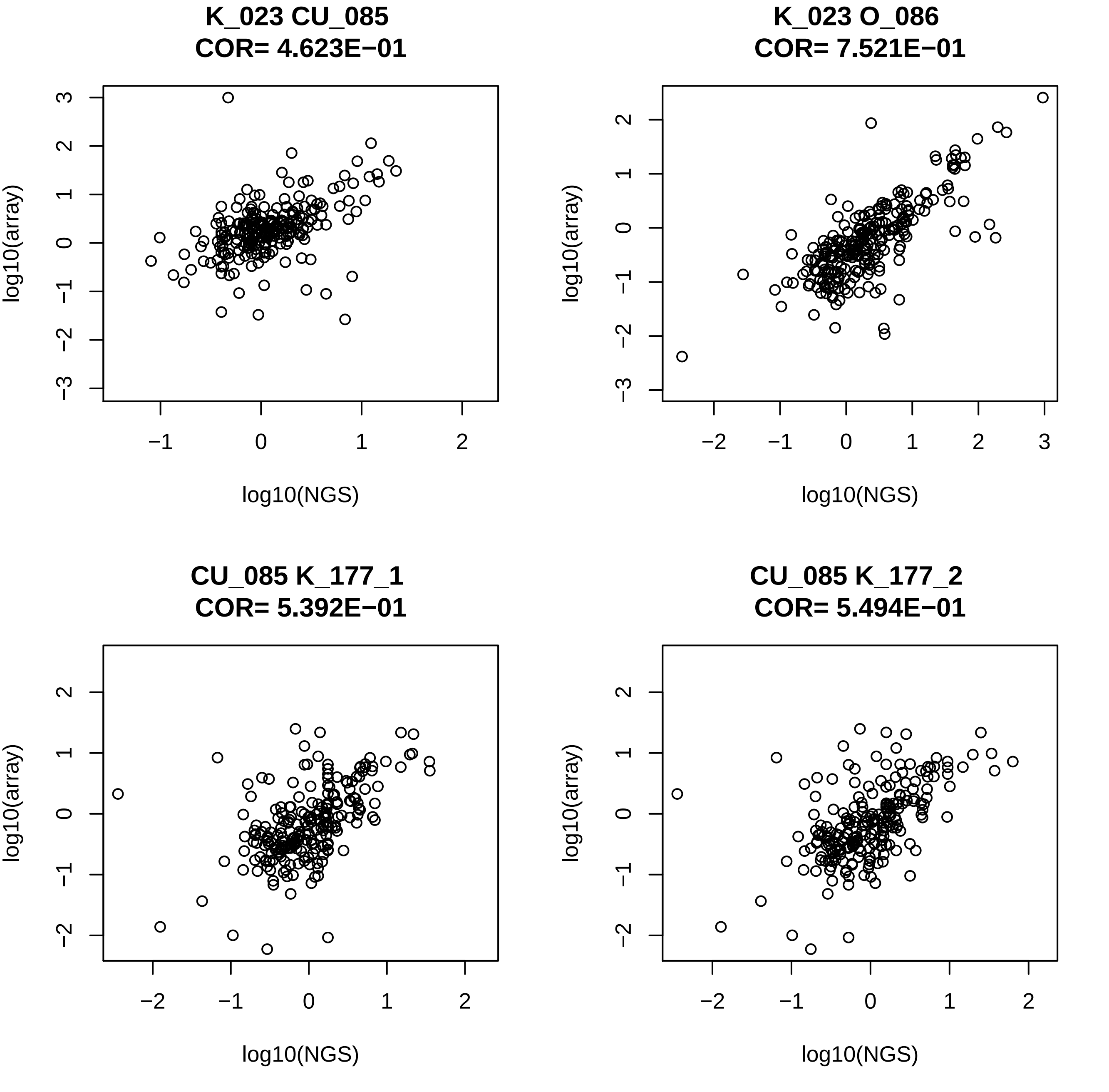
K_023 CU_085
COR= 4.623E−01
K_023 O_086
COR= 7.521E−01
CU_085 K_177_1
COR= 5.392E−01
CU_085 K_177_2
COR= 5.494E−01
log10(array)
log10(NGS)

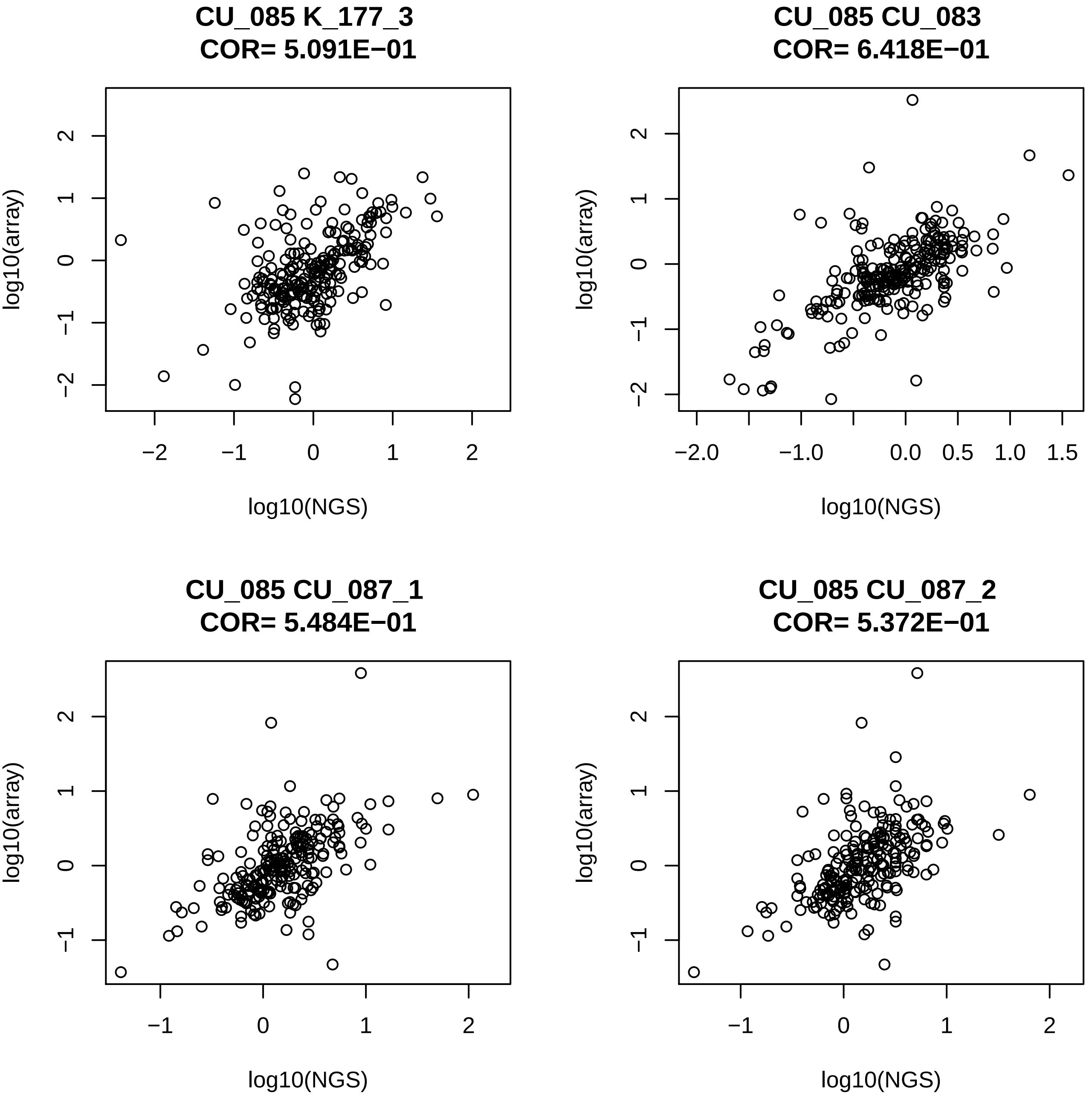
CU_085 K_177_3
COR= 5.091E−01
log10(array)
log10(NGS)
CU_085 CU_083
COR= 6.418E−01
log10(array)
log10(NGS)
CU_085 CU_087_1
COR= 5.484E−01
log10(array)
log10(NGS)
CU_085 CU_087_2
COR= 5.372E−01
log10(array)
log10(NGS)

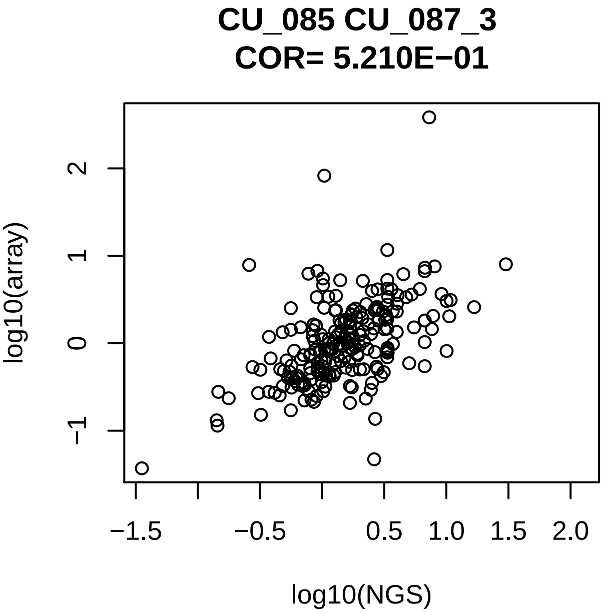
CU_085 CU_087_3
COR= 5.210E−01
log10(array)
log10(NGS)
−1.5 −0.5 0.5 1.0 1.5 2.0
−1 0 1 2

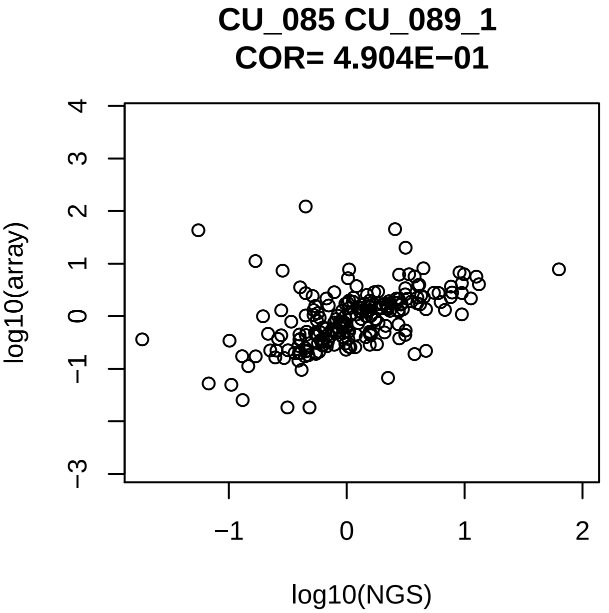
CU_085 CU_089_1
COR= 4.904E−01
log10(array)
log10(NGS)
−1 0 1 2
−3 −1 0 1 2 3 4

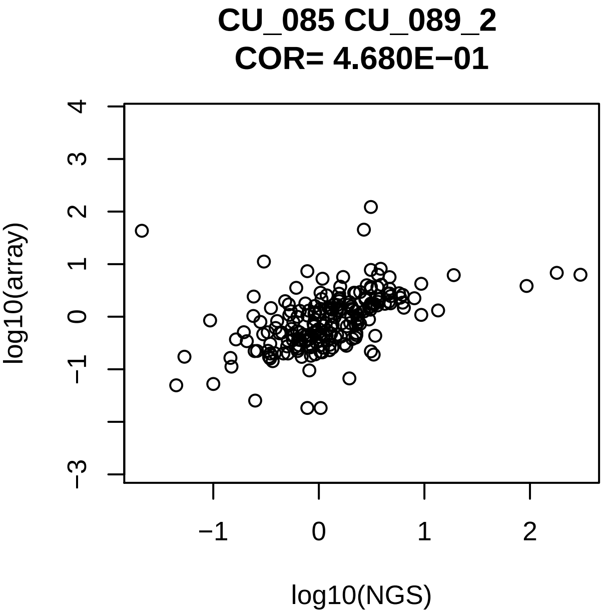
CU_085 CU_089_2
COR= 4.680E−01
log10(array)
log10(NGS)
−1 0 1 2
−3 −1 0 1 2 3 4

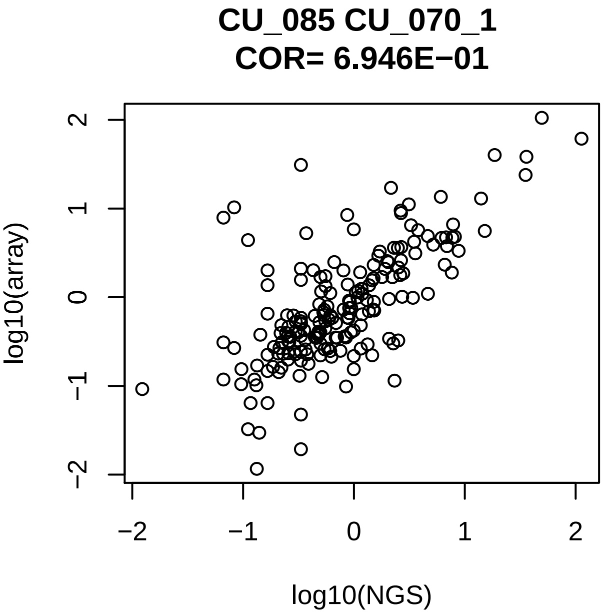
CU_085 CU_070_1
COR= 6.946E−01
log10(array)
log10(NGS)
−2 −1 0 1 2
−2 −1 0 1 2

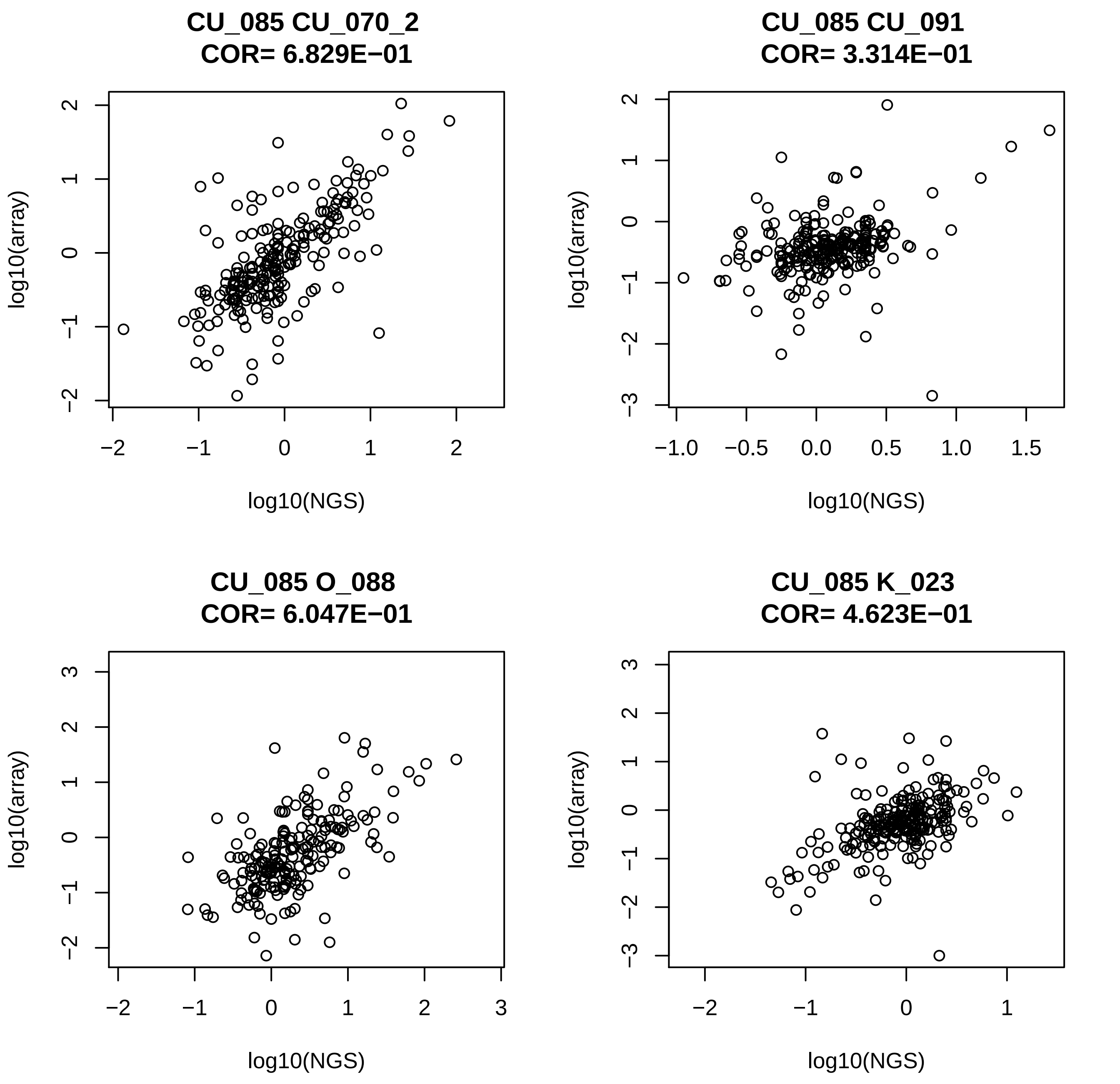
CU_085 CU_070_2
COR= 6.829E−01
log10(array)
log10(NGS)
CU_085 CU_091
COR= 3.314E−01
log10(array)
log10(NGS)
CU_085 O_088
COR= 6.047E−01
log10(array)
log10(NGS)
CU_085 K_023
COR= 4.623E−01
log10(array)
log10(NGS)

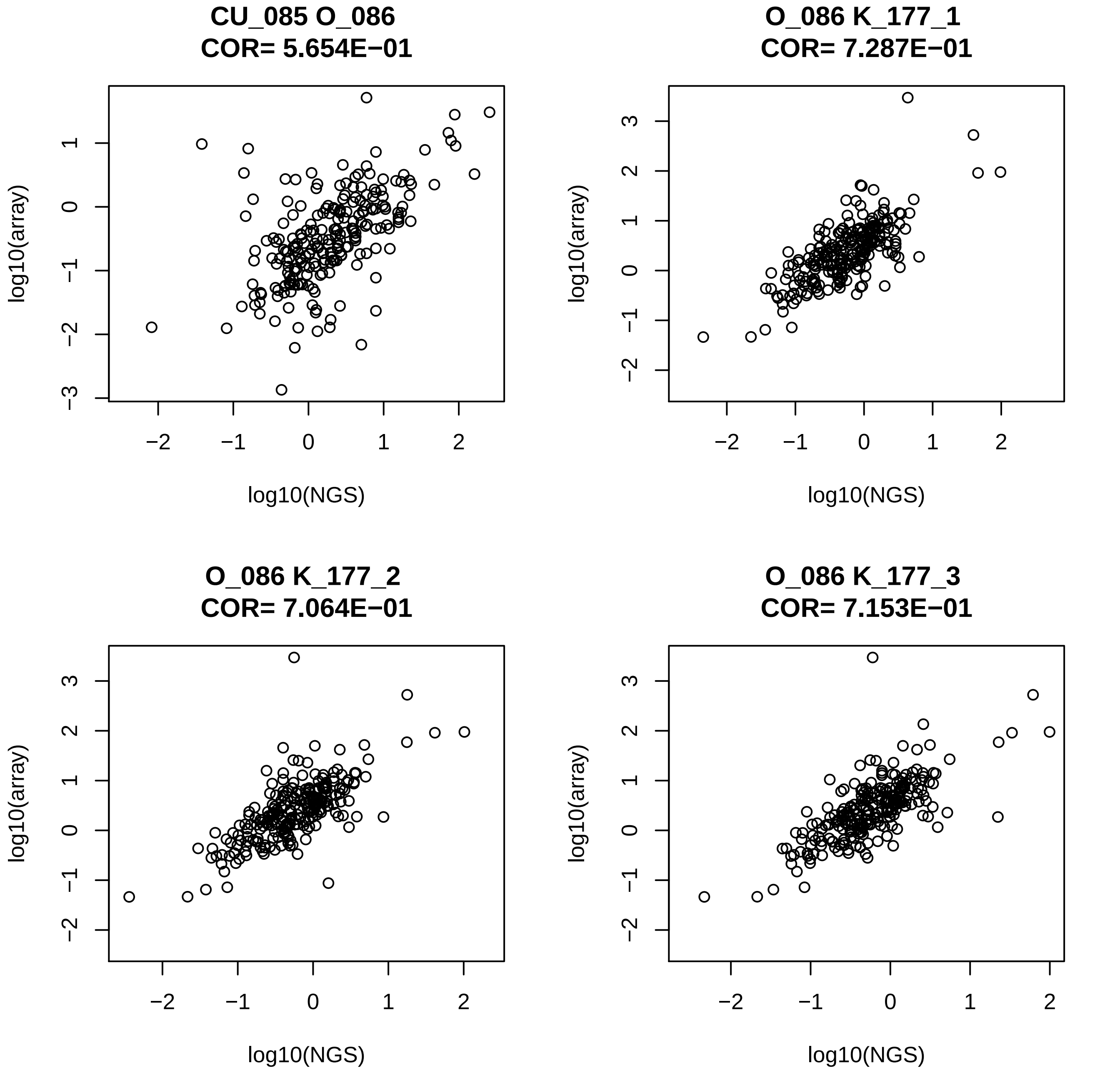
CU_085 O_086
COR= 5.654E−01
log10(array)
log10(NGS)
O_086 K_177_1
COR= 7.287E−01
log10(array)
log10(NGS)
O_086 K_177_2
COR= 7.064E−01
log10(array)
log10(NGS)
O_086 K_177_3
COR= 7.153E−01
log10(array)
log10(NGS)

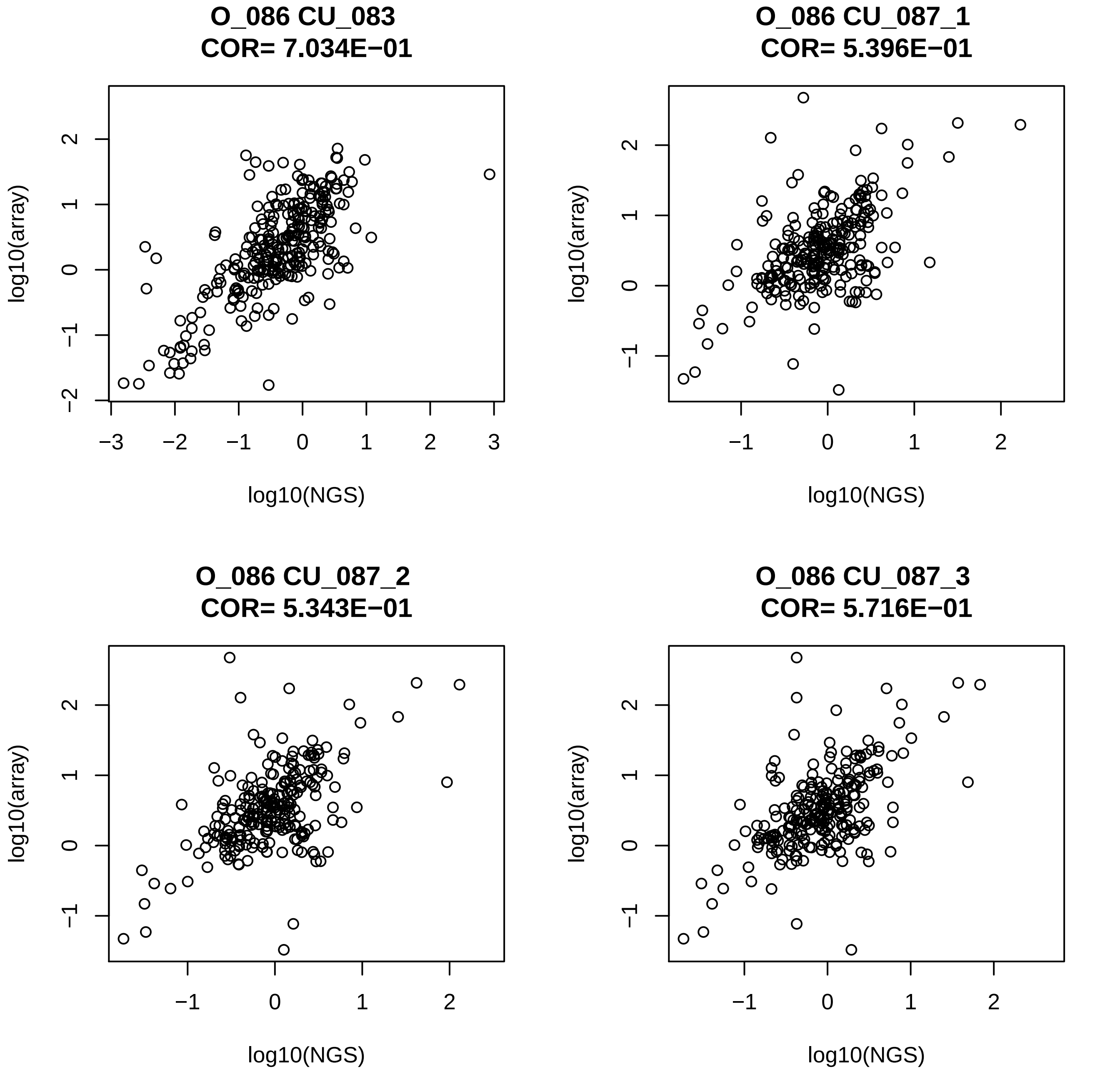
O_086 CU_083
COR= 7.034E−01
log10(array)
log10(NGS)
O_086 CU_087_1
COR= 5.396E−01
log10(array)
log10(NGS)
O_086 CU_087_2
COR= 5.343E−01
log10(array)
log10(NGS)
O_086 CU_087_3
COR= 5.716E−01
log10(array)
log10(NGS)

O_086 CU_089_1
COR= 6.633E−01

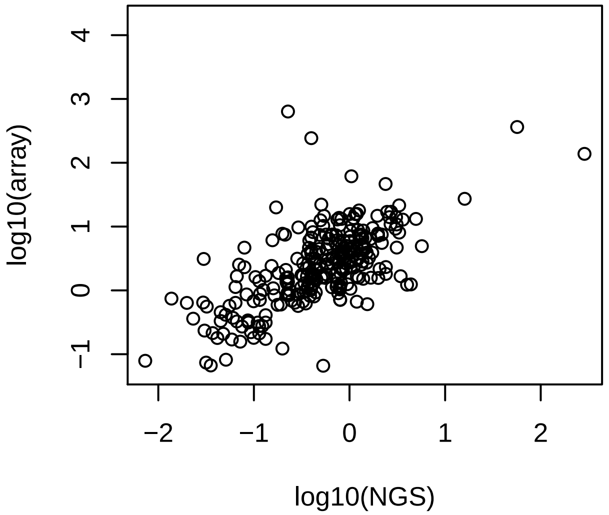

O_086 CU_089_2
COR= 4.197E−01

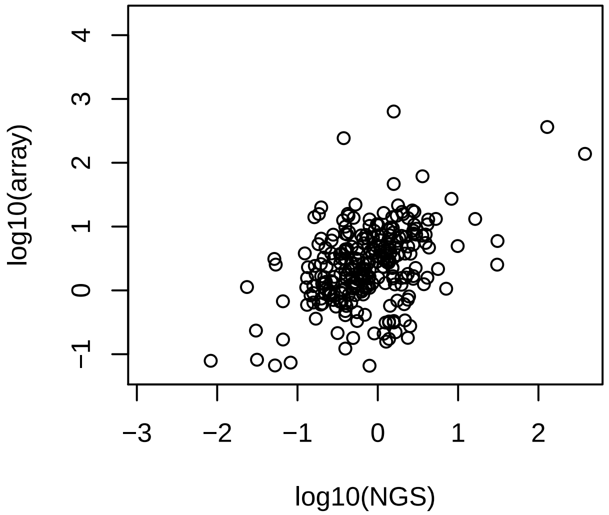

O_086 CU_070_1
COR= 6.819E−01

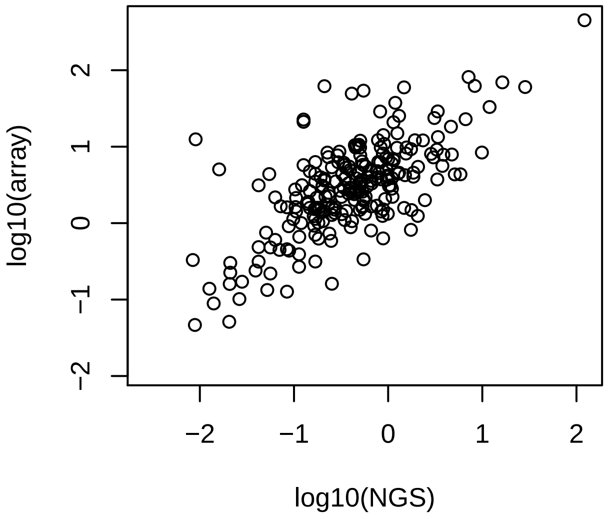

O_086 CU_070_2
COR= 6.560E−01

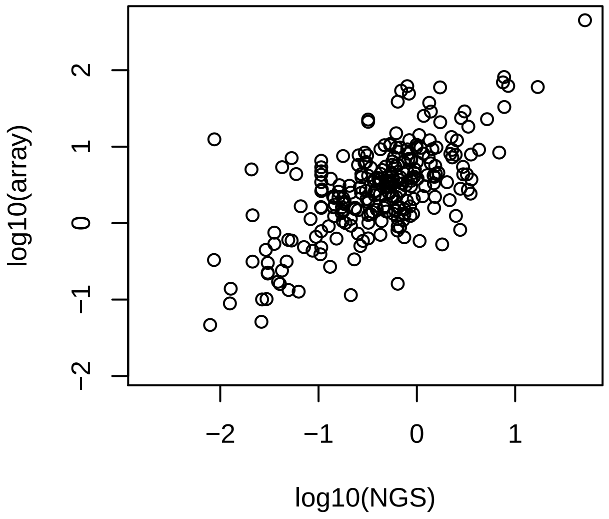

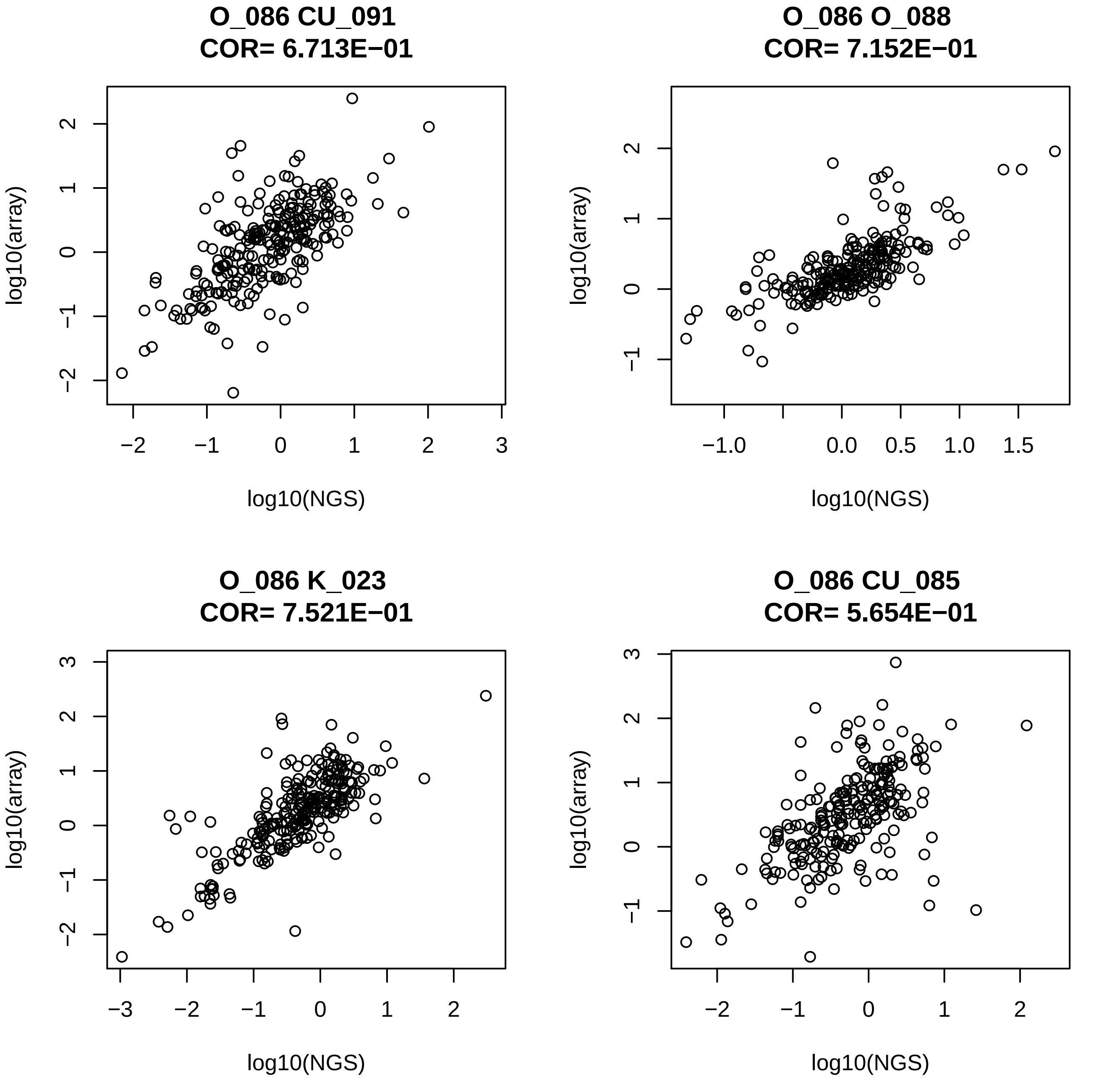
O_086 CU_091
COR= 6.713E−01
log10(array)
log10(NGS)
O_086 O_088
COR= 7.152E−01
log10(array)
log10(NGS)
O_086 K_023
COR= 7.521E−01
log10(array)
log10(NGS)
O_086 CU_085
COR= 5.654E−01
log10(array)
log10(NGS)
