# Supplementary figures and images for: Comparison of Hepatocellular Carcinoma miRNA Expression Profiling as Evaluated by Next Generation Sequencing and Microarray
Source: PLoS One. 2014 Sep 12;9(9):e106314. doi: 10.1371/journal.pone.0106314 (PMC4162537; doi:10.1371/journal.pone.0106314)

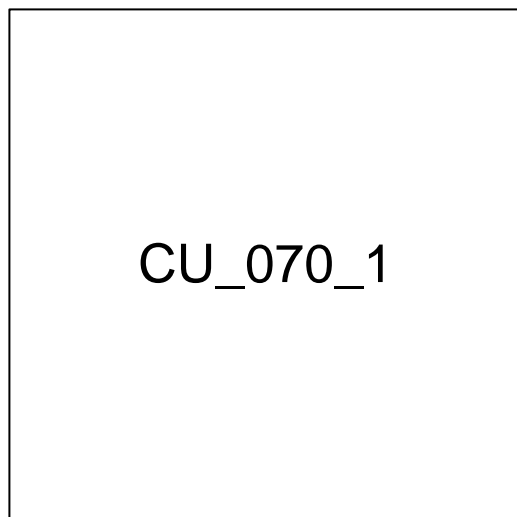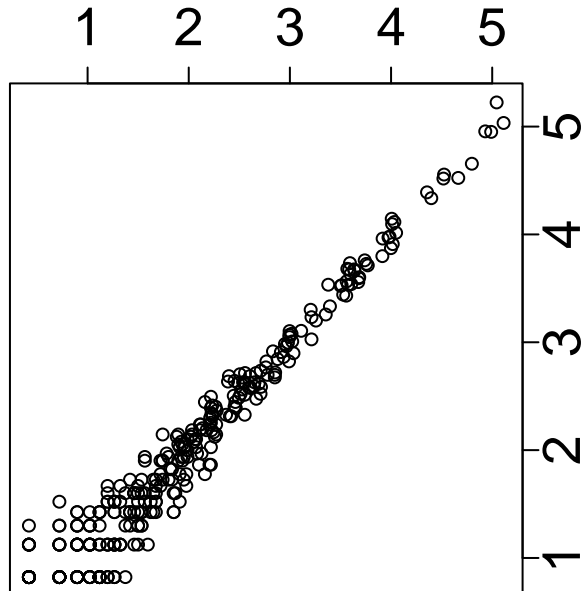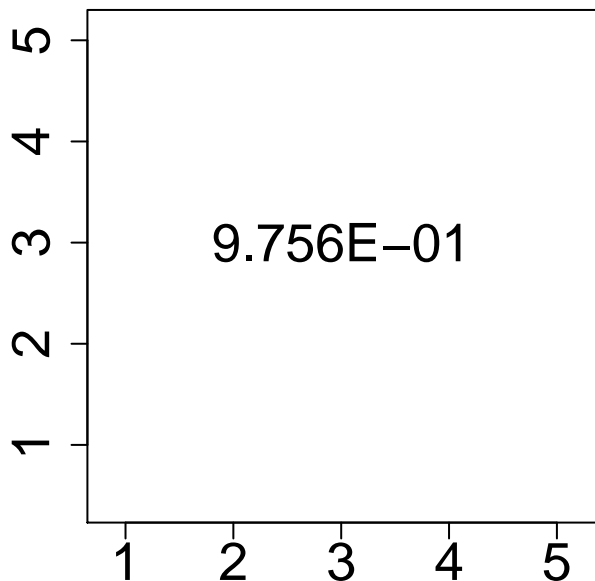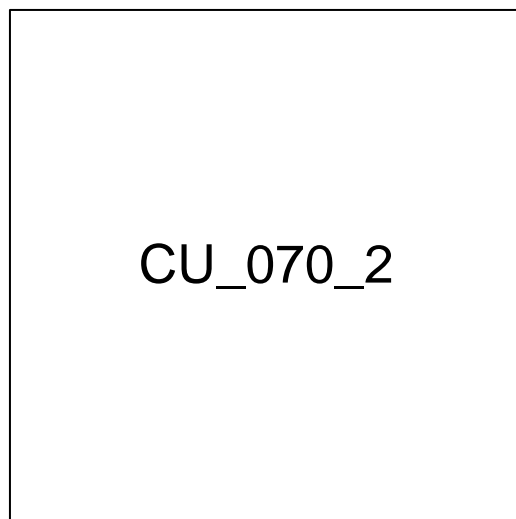

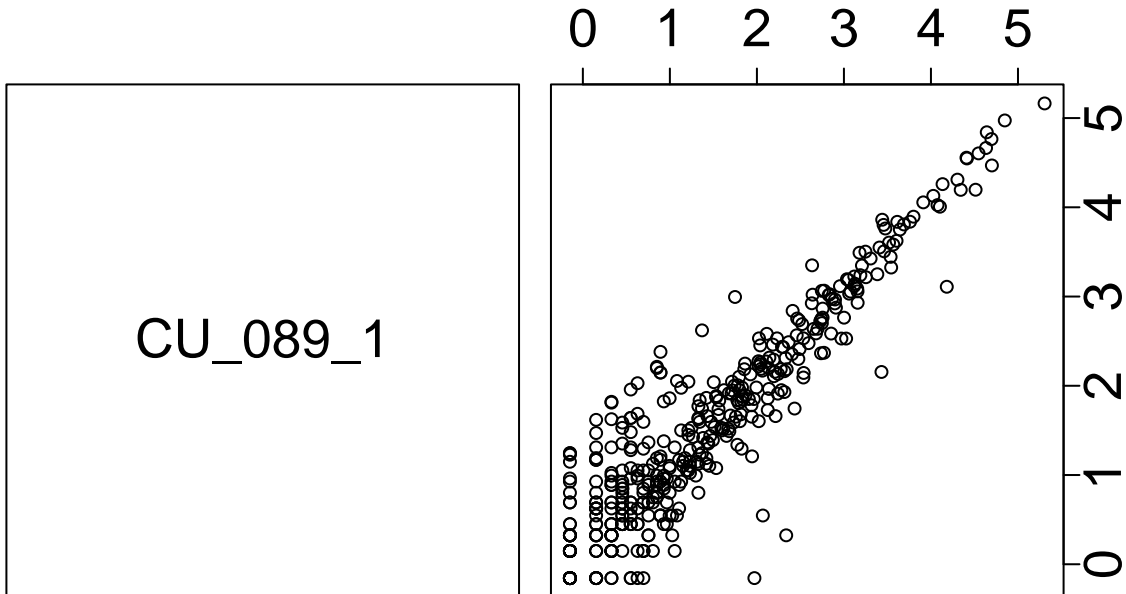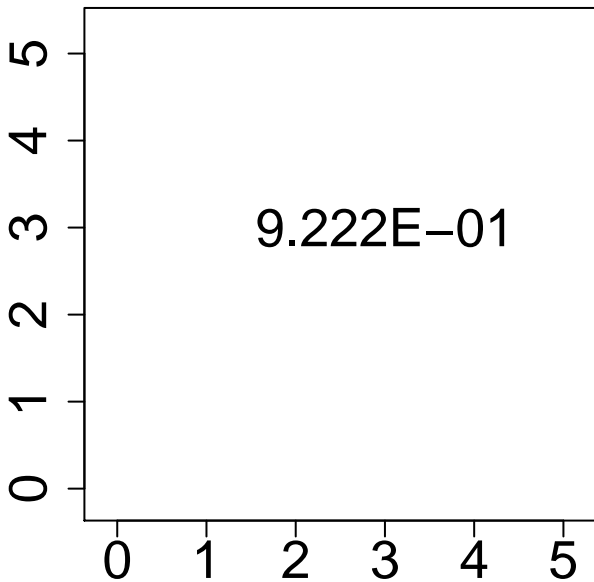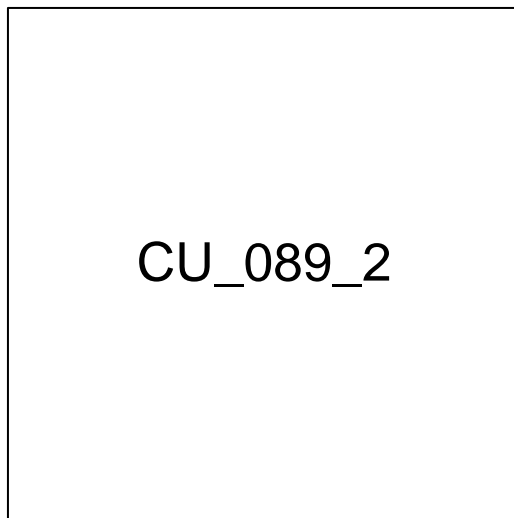

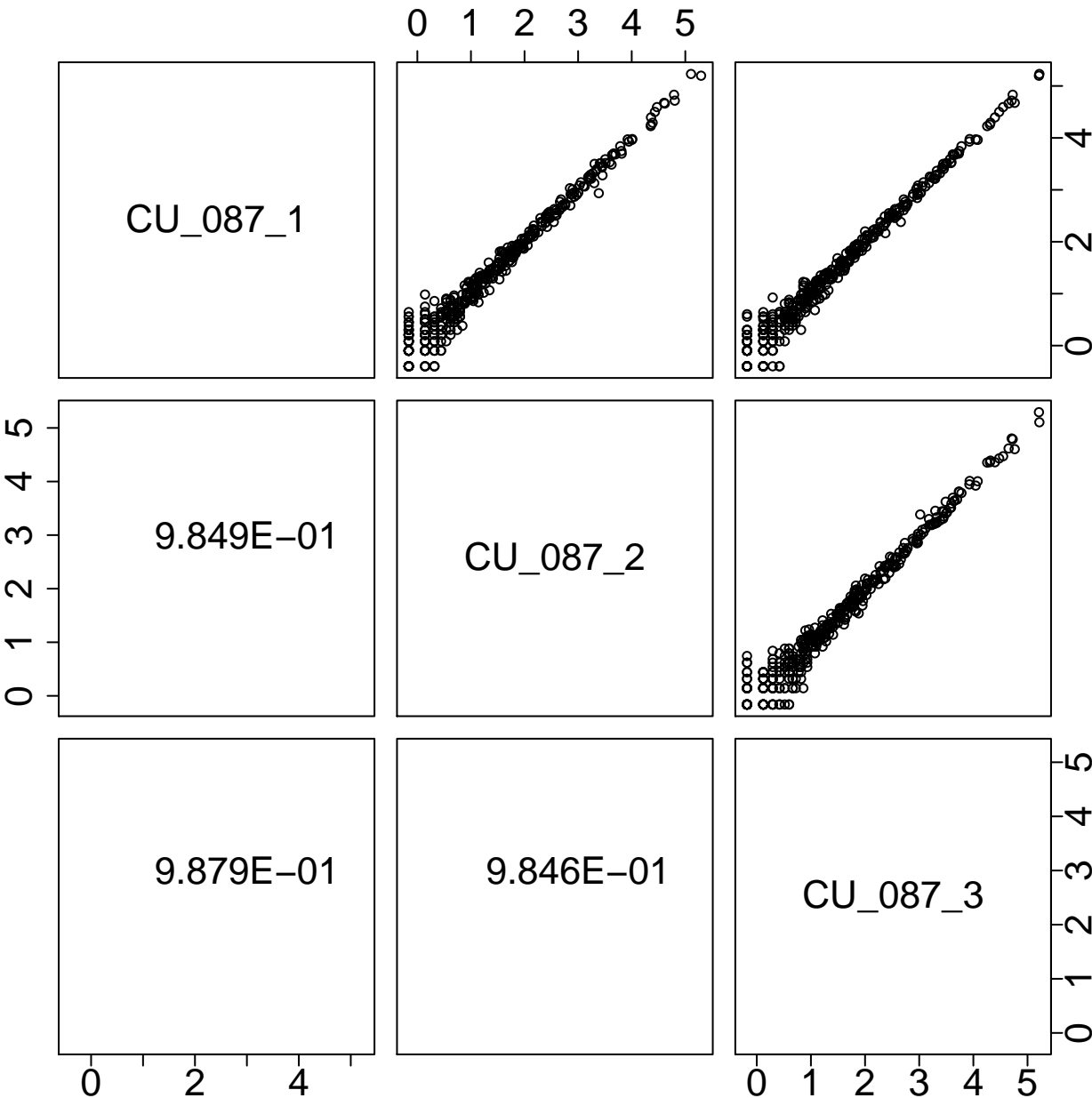

Supplement: Figure S3 — Comparison of logarithmic miRNA expression in HCC for NGS technical replicates not included in Fig. 3. Comparison between differential logarithmic HCC miRNA expression in NGS (horizontal axis) and microarray (vertical) analysis. One black circle showed one miRNA. (PDF) [file pone.0106314.s003.pdf]

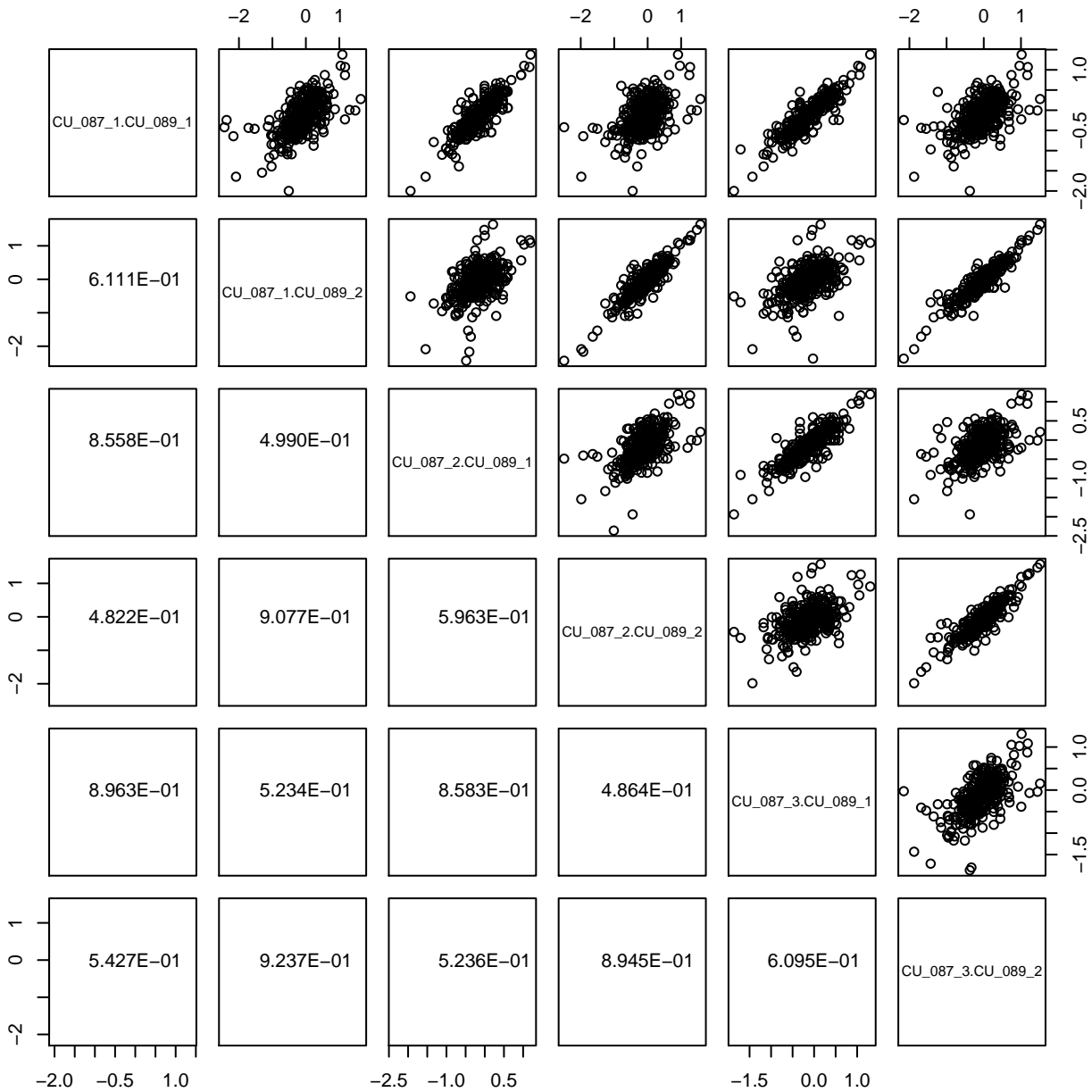

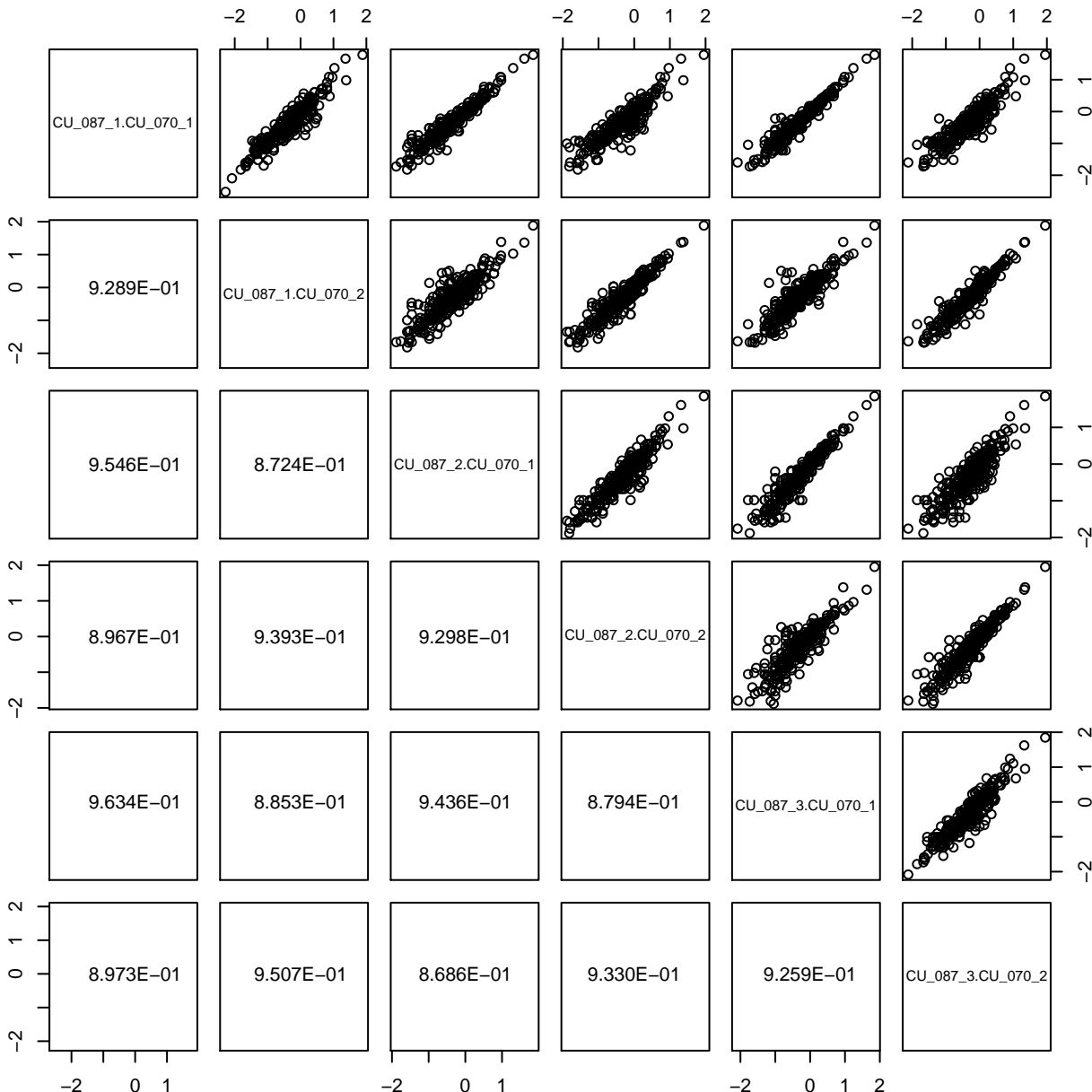

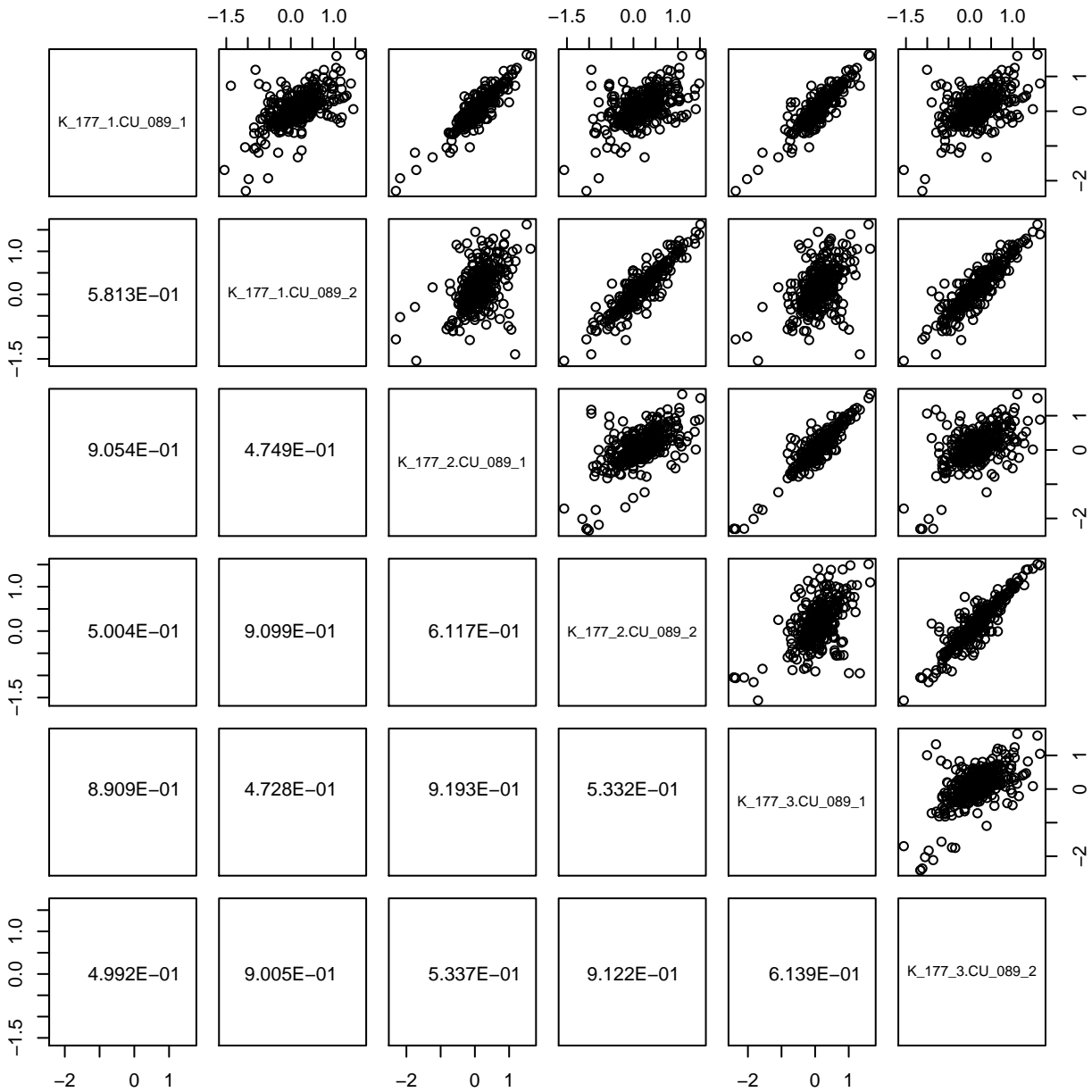

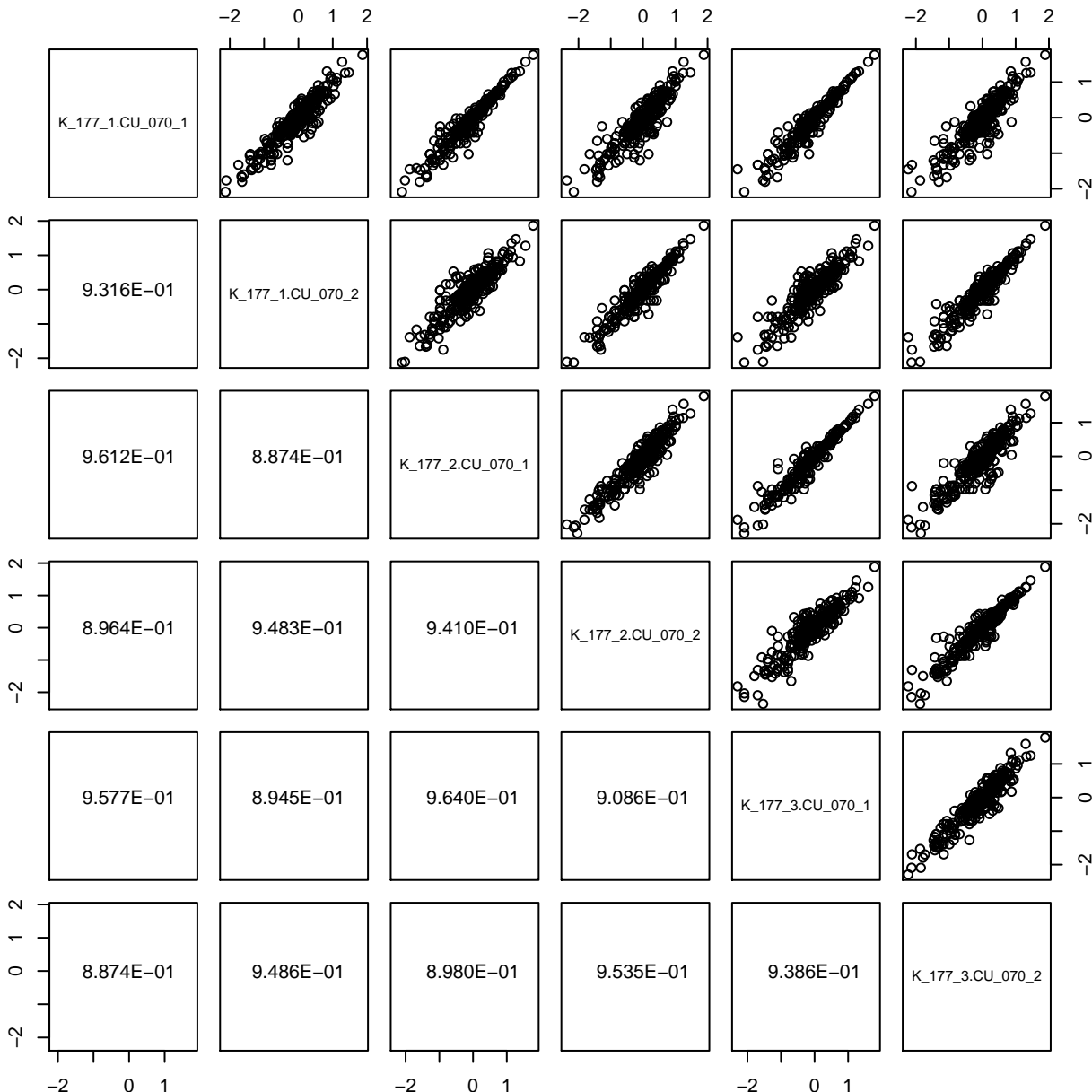

Supplement: Figure S4 — Comparison of differential logarithmic miRNA expression in HCC for NGS technical replicates not included in Fig. 4. Comparison between differential logarithmic HCC miRNA expression in NGS (horizontal axis) and microarray (vertical) analysis. One black circle showed one miRNA. (PDF) [file pone.0106314.s004.pdf]
