# Supplementary material for: Comparison of Hepatocellular Carcinoma miRNA Expression Profiling as Evaluated by Next Generation Sequencing and Microarray
Source: PLoS One. 2014 Sep 12;9(9):e106314. doi: 10.1371/journal.pone.0106314 (PMC4162537; doi:10.1371/journal.pone.0106314)
Supplement: Table S2 — Detailed NGS analysis of HCC and non-tumorous tissue samples. (PDF) [file pone.0106314.s006.pdf]

Table S2. Detailed NGS analysis of HCC and non tumorous tussue samples

		data of analysis				total read	ter adapter trimmi	total	mapped	mapped %
new8	5	20121010	4	K_147T	A01	236669	165189	124370	31193	0.251
new8			5	K_175N	B02	513159	500291	411059	270287	0.658
new8			6	K_175T	C03					
new8			7	K_177N	D04	1132511	1117585	1002655	899808	0.897
new8			8	K_177T	E05	1041353	1028339	937914	752837	0.803
new8			9	K_181N	F06					
new9	6	20121129	5	K_175N	A01	848734	827669	683835	443188	0.648
new9			6	K_175T	B02	1120403	1075142	882415	592130	0.671
new9			7	K_177N	C03	2085193	2056282	1842982	1659437	0.9
new9			8	K_177T	D04	1898556	1873391	1709628	1370515	0.802
new10	7	20130116	7	K_177N	A01	4873149	4817016	4327925	3863410	0.893
new10			8	K_177T	B02	2470578	2443094	2236088	1777582	0.795
new10			9	K_181N	C03	926097	905574	877517	408563	0.534
new10			10	K_181T	D04	3018936	2910741	2801661	2277665	0.813
new11	11	20130131	21	CU_083N	A01	1870430	1805841	1608246	1250467	0.778
new11			22	CU_83T	B02	2754006	2699756	2548095	1115085	0.438
new11			25	CU_87N	C03	4654332	4492341	4076840	1656764	0.406
new11			26	CU_87T	D04	3140381	3070449	2872307	2323261	0.809
new12	12	20130206	25	CU_87N	A01	3858103	3755826	3494016	1019216	0.292
new12			26	CU_87T	B02	1989055	1953136	1853488	1293434	0.698
new12			27	CU_89N	C03	2255561	2207264	2045983	1515610	0.741
new12			28	CU_89T	D04	2020708	1911504	1724445	1302904	0.756
new12	13	20130404	20	CU_70T	A01	1234937	1194349	802318	324006	0.404
new12			26	CU_87T	B02	2072748	1993177	1870675	1375184	0.735
new12			30	CU_91T	C03	2337144	2234907	2152538	816614	0.621
new12			41	O_088T	D04	2003938	1813351	1654822	1242229	0.751
new12			43	O_089T	E05	1762310	1554010	1407269	1171099	0.832
new12	14	20130425	18	K_023T	A01	2028373	1990347	1898619	977110	0.515
new12			20	CU_070T	B02	2980176	2913472	1931102	788274	0.408
new12			24	CU_085T	C03	1339174	1300369	1224320	448060	0.366
new12			38	O_086T	D04	5618507	5224066	4642687	3491017	0.752

data of analysis	sample name	total read	after adapter trimming	total	mapped	mapped %
20121010	K_147T	236669	165189	124370	31193	0.251
	K_175N	513159	500291	411059	270287	0.658
	K_175T					
	K_177N	1132511	1117585	1002655	899808	0.897
	K_177T	1041353	1028339	937914	752837	0.803
	K_181N					
20121129	K_175N	848734	827669	683835	443188	0.648
	K_175T	1120403	1075142	882415	592130	0.671
	K_177N	2085193	2056282	1842982	1659437	0.9
	K_177T	1898556	1873391	1709628	1370515	0.802
20130116	K_177N	4873149	4817016	4327925	3863410	0.893
	K_177T	2470578	2443094	2236088	1777582	0.795
	K_181N	926097	905574	877517	408563	0.534
	K_181T	3018936	2910741	2801661	2277665	0.813
20130131	CU_083N	1870430	1805841	1608246	1250467	0.778
	CU_83T	2754006	2699756	2548095	1115085	0.438
	CU_87N	4654332	4492341	4076840	1656764	0.406
	CU_87T	3140381	3070449	2872307	2323261	0.809
20130206	CU_87N	3858103	3755826	3494016	1019216	0.292
	CU_87T	1989055	1953136	1853488	1293434	0.698
	CU_89N	2255561	2207264	2045983	1515610	0.741
	CU_89T	2020708	1911504	1724445	1302904	0.756
20130404	CU_70T	1234937	1194349	802318	324006	0.404
	CU_87T	2072748	1993177	1870675	1375184	0.735
	CU_91T	2337144	2234907	2152538	816614	0.621
	O_088T	2003938	1813351	1654822	1242229	0.751
	O_089T	1762310	1554010	1407269	1171099	0.832
20130425	K_023T	2028373	1990347	1898619	977110	0.515
	CU_070T	2980176	2913472	1931102	788274	0.408
	CU_085T	1339174	1300369	1224320	448060	0.366
	O_086T	5618507	5224066	4642687	3491017	0.752
