# Supplementary material for: Comparison of Hepatocellular Carcinoma miRNA Expression Profiling as Evaluated by Next Generation Sequencing and Microarray
Source: PLoS One. 2014 Sep 12;9(9):e106314. doi: 10.1371/journal.pone.0106314 (PMC4162537; doi:10.1371/journal.pone.0106314)
Supplement: Table S1 — Extracted read counts of each miRNA obtained by NGS analysis. (PDF) [file pone.0106314.s005.pdf]

Table S1. Extracted reads counts of each miRNA obtained by NGS analysis

miRNA	K_177_1	K_177_2	K_177_3	CU_083	CU_087_1	CU_087_2	CU_087_3	CU_089_1	CU_089_2	CU_070_1	CU_070_2	CU_091	O_088	K_023	CU_085	O_086
hsa-miR-21	44814.17	46645.22	47105.32	33106.88	39236.7	29647.11	35409.87	15697.37	21979.85	90332.75	85372.1	31167.23	30826.68	33646.98	20337.84	21467.17
hsa-miR-451	4320.11	4623.84	4899.95	3423.47	1631.6	1750.91	1942.99	2238.66	430.46	1197.43	1037.89	4452.6	546.2	2480.9	2750.15	281.08
hsa-miR-122	15901.72	15894.94	15288.89	7598.21	9509.48	10254.76	8540.86	10155.96	12683.41	21686.78	24982.86	3309.7	25932.93	5473.46	10589.72	7336.01
hsa-miR-720	0	0.7	1.61	1.9	0.4	1.39	1.32	1.41	0.71	0	0	0.98	0.74	0	0	0.28
hsa-let-7a	12713.31	12420.26	12654	16098.88	16880.6	22629.6	17999.93	18196.42	13595.39	6293.16	8205.87	8858.98	17995.3	22223.98	15835.99	17976.27
hsa-miR-923	NA	NA	NA	NA	NA	NA	NA	NA	NA	NA	NA	NA	NA	NA	NA	NA
hsa-miR-22	33156.94	33907.49	34385.93	17630.88	47392.28	40211.47	58068.63	20420.3	20153.04	13989.97	10163.41	22008.26	23043.64	25636.55	26719.5	15549.15
hsa-miR-192	144252.23	147777.42	149416.8	64370.78	170300.19	127830.6	165476.91	145968.97	202213.3	167573.61	110625.6	34266.44	335302.47	60065.71	81470.63	163702.43
hsa-let-7f	15929.45	15216.06	15537.05	19779.11	19591.22	24556.44	20682.89	11365.02	8166.03	7463.98	9968.97	8972.09	22155.87	27930.58	16598.08	35428.23
hsa-miR-16	9208.58	9643.3	9596.76	4072.03	5023.79	4619.01	5003.49	6877.85	4098.97	4743.15	4317.08	3379.53	2789.18	3725.8	2732.48	2468.03
hsa-miR-19b	558.29	558.18	581.54	924.34	324.71	395.48	288.75	1049.31	436.85	1064.38	1634.34	518.34	203.17	1028.67	971.94	229.79
hsa-miR-122*	233.14	228.16	258.88	152.15	193.3	263.19	185.48	145.68	207.08	412.45	491.35	72.78	147.22	176.19	262.87	133.95
hsa-miR-20a	210.46	231.65	220.82	273.88	59.88	41.56	44.73	143.57	161.69	325.97	357.35	84.59	27.97	193.1	97.19	23.54
hsa-miR-26b	4649.03	4566.63	4368.79	3108.7	3877.25	3197.08	3690.62	5632.19	4392.57	3672.12	3639.17	3680.5	4584.58	3433.93	4177.13	5754.36
hsa-miR-142-3p	187.78	208.62	194.56	233.94	110.92	101.81	107.87	125.97	62.41	306.01	312.68	270.48	13.25	415.56	287.16	27.18
hsa-miR-19a	180.21	251.18	270.13	419.38	75.95	87.96	71.69	382.14	129.78	498.93	601.71	167.21	55.21	422.68	236.36	86.59
hsa-miR-15b	173.91	199.55	189.2	138.84	174.01	141.98	149.97	196.35	156.02	419.1	307.42	89.5	58.15	79.2	79.52	77.35
hsa-miR-34a	221.8	230.95	226.18	75.13	356.86	376.78	317.03	123.86	341.82	379.19	409.9	129.83	548.41	207.33	342.39	333.2
hsa-miR-148a	60794.03	58198.9	56632.94	22186.95	67850.74	61604.07	53265.09	58217.14	49572.73	45256.18	62948.49	7107.25	47270.23	21985.5	17114.97	210120.37
hsa-miR-107	1909.27	1970.38	2069.43	875.84	1160.2	1324.27	1304.97	1539.12	1126.15	3386.06	3245.04	1338.63	1351.52	969.05	1062.51	1832.18
hsa-miR-15a	694.39	717.26	749.3	365.17	405.09	385.09	374.26	567.23	290.05	425.75	359.98	402.28	266.48	351.49	225.31	184.68
hsa-miR-29a	1119.09	1103.81	994.25	751.26	1743.32	1540.36	1679.89	1306.88	900.64	1170.82	987.96	834.06	839.18	914.76	1217.13	977.18
hsa-let-7g	2873.35	2775.56	2948.97	6364.81	4975.57	6498.05	5388.93	2227.4	1611.22	2767.39	3363.28	3065.77	3743.19	5997.58	4963.52	4839.11
hsa-miR-215	1238.82	875.65	939.04	250.1	737.84	853.99	753.12	927.56	789.3	731.76	867.1	285.23	1072.53	376.41	865.91	1104.97
hsa-miR-23b	986.77	1084.27	1038.2	3145.79	3280.07	3244.18	3866.9	1779.11	2418.96	4803.02	3696.98	1461.58	2034.65	1960.34	1230.39	672.29
hsa-miR-126	1816.01	1843.4	1735.51	5431.91	3266.41	2665.85	3266.38	1225.25	1420.46	4284.14	3975.5	5922.05	800.9	3763.17	2619.82	1240.32
hsa-miR-27b	60660.44	59805.07	58662.17	29996.27	31663.04	27058.14	29961.08	69375.95	43819.99	32969.23	32936.48	16685.21	31803.52	39729.98	27925.59	30643.76
hsa-let-7b	1092.63	1059.15	1100.37	2317.5	1904.87	2845.24	1979.16	1646.8	1787.8	472.32	709.44	1645.51	2019.93	3013.92	3649.19	1596.5
hsa-miR-223	332.7	344.68	316.23	186.39	86.8	90.73	78.93	76.71	34.04	126.4	97.22	498.67	11.78	301.66	101.61	17.94
hsa-miR-92a	50690.68	51238.36	51057.65	41656.05	6922.23	6104.65	6026.95	29458.69	50152.12	10364.42	11214.43	3305.76	7435.59	23111.16	7291.76	10274.28
hsa-miR-24	482.67	497.48	489.89	918.63	835.89	777.11	776.14	1061.27	687.18	1017.82	1053.65	573.42	739.07	841.8	821.73	443.89
hsa-miR-146b-5p	66162.65	63469.54	67094.77	50810.98	157797.97	196860.13	162505.2	13446.04	10642.43	108054.71	129578.2	11733.94	9175.05	20925.69	22988.58	19724.94
hsa-miR-23a	177.69	205.83	194.56	307.16	287.74	275.66	351.9	225.91	252.46	279.4	144.52	276.38	220.84	351.49	419.7	89.4
hsa-miR-130b	1954.63	1895.03	1861.47	135.99	55.86	47.1	58.54	231.54	545.35	252.79	183.93	73.77	139.86	99.66	24.3	187.2
hsa-miR-103	3712.67	3958.91	4049.34	2482.02	3401.84	3889.69	3940.57	4024.1	3296.2	9160.34	8258.43	2621.2	2798.75	2914.26	2440.89	3483.05
hsa-miR-17	265.91	256.76	272.28	361.37	57.07	46.4	58.54	153.42	219.84	332.62	325.82	70.82	27.97	176.19	83.94	34.47
hsa-miR-1274b	NA	NA	NA	NA	NA	NA	NA	NA	NA	NA	NA	NA	NA	NA	NA	NA
hsa-miR-30b	812.86	840.06	831.31	715.13	1183.11	1254.31	1135.27	750.91	809.16	791.63	1085.18	553.75	2356.33	953.03	1206.09	273.79
hsa-miR-106b	189.04	161.87	177.95	188.29	114.13	88.65	98	154.83	112.05	379.19	394.13	94.42	30.18	101.44	97.19	134.23
hsa-miR-29b	42.85	41.17	47.17	68.47	104.08	98.35	104.58	60.52	86.52	119.74	84.08	96.39	117.04	76.53	181.13	22.98
hsa-miR-320d	10.08	7.68	8.58	1.9	1.61	2.77	1.97	8.45	2.13	13.3	7.88	0	4.42	2.67	0	4.76
hsa-let-7i	3221.18	3162.1	3279.67	7727.54	3067.48	4202.06	3078.26	3095.84	1511.23	2707.52	3602.39	4685.71	1198.41	14000.89	5411.94	4186.72
hsa-miR-151-5p	3797.11	3741.22	3662.9	1993.22	3161.52	2025.88	2771.75	3544.84	2565.05	1589.92	1810.39	491.78	1740.2	1489.61	762.09	3905.64
hsa-miR-194	2998.11	2915.81	2863.75	1712.69	4821.25	4979.16	4172.75	3214.07	2891.26	3924.91	4795.3	844.88	3141.78	2182.8	1897.49	3931.71
hsa-miR-27a	2039.07	2145.51	1969.2	1527.25	1397.31	1124.8	1199.73	1564.46	1083.6	918.03	922.27	949.14	661.04	1671.14	1524.18	529.08
hsa-miR-29c	161.31	173.04	150.61	182.59	340.38	284.66	324.93	173.83	109.92	246.14	165.54	241.96	179.61	249.16	371.1	110.97
hsa-miR-125b	781.35	804.48	726.79	787.4	653.84	480.67	517.65	579.9	1002.76	53.22	89.34	338.35	1330.91	645.14	806.27	1516.92
hsa-miR-1915	0	0.7	0	0	0	0	0	0	0	0	0	0	0	0	0	0
hsa-miR-30d	11487.1	11389.02	11196.13	4751.97	5589.23	6432.26	5505.35	7851.85	6301.64	3465.89	4046.44	3250.68	5788.88	5937.96	4731.58	3532.09
hsa-miR-551b	17.64	21.63	31.62	8.56	0	0	0	2.82	0.71	6.65	13.14	0	0	0	0	0
hsa-miR-26a	30100.86	29160.85	26221.32	60671.53	45902.94	41469.94	44749.89	46236.33	42951.97	35916.23	33419.96	188307.57	39867.76	35707.87	42809.52	28025.24
hsa-miR-144	267.17	280.49	280.85	198.75	81.18	64.41	77.61	111.19	16.31	86.48	84.08	239.99	15.46	113.9	309.25	17.37
hsa-miR-331-3p	21.42	11.86	18.76	10.46	3.62	6.23	3.95	7.74	12.76	0	5.26	1.97	2.94	4.45	4.42	1.68
hsa-let-7d	283.55	313.28	301.76	694.2	317.88	379.55	392.68	188.61	104.25	106.44	165.54	181.96	145.02	727.01	278.33	410.83
hsa-miR-20b	5.04	2.09	4.82	2.85	1.61	1.39	0.66	1.41	2.13	13.3	7.88	3.93	0.74	2.67	6.63	1.12
hsa-miR-1207-5p	0	0	0	0	0	0	0	0	0	0	0	0	0	0	0	0
hsa-let-7c	1207.31	1144.97	1154.51	2227.16	1346.67	1996.1	1514.79	1312.51	1314.79	332.62	512.37	996.35	3836.68	2260.22	2049.91	3972.34
hsa-miR-25	4449.91	4362.89	4383.8	2884.28	1953.09	1697.58	2121.24	3828.45	3689.07	5421.69	3922.95	1224.54	2180.4	1393.5	1214.92	5165.58
hsa-miR-320c	117.2	145.13	130.24	39.94	18.49	14.54	13.81	38.71	53.9	79.83	55.18	21.64	23.56	26.7	17.67	21.58
hsa-miR-885-5p	123.5	154.9	120.6	19.02	15.67	18.7	11.84	76.71	67.37	26.61	18.39	9.84	11.78	8.9	90.57	0.56
hsa-miR-224	772.53	806.57	812.01	235.84	802.14	941.26	936.63	491.22	331.18	13.3	10.51	15.74	82.45	119.24	33.13	17.37
hsa-miR-320b	278.51	286.07	276.03	92.24	39.38	33.94	36.18	72.49	133.32	119.74	112.99	64.92	58.89	71.19	59.64	38.39
hsa-miR-199a-3p	764.97	784.94	750.37	2967.01	1674.2	1600.62	1832.49	1736.88	1544.56	206.22	262.76	5679.11	70.67	6357.08	7417.67	102.01
hsa-miR-1260	13.86	14.65	11.79	32.33	3.62	4.16	3.95	3.52	9.93	19.96	5.26	15.74	2.21	11.57	11.04	4.2
hsa-miR-365	99.56	108.15	104.52	55.16	73.54	96.97	69.72	88.67	52.48	33.26	81.45	13.77	68.46	42.71	48.6	125.27
hsa-miR-101	3169.51	3269.55	3244.84	2062.64	4721.18	4436.85	4524.65	5790.54	2988.42	5335.21	5699.18	4356.21	6356.43	4003.43	6975.88	4186.72
hsa-miR-195	209.2	175.83	174.73	402.26	308.64	290.2	284.15	234.35	589.32	153	128.75	838	125.88	438.7	929.97	135.91
hsa-miR-93	1308.13	1370.34	1320.66	1037.5	515.2	515.99	581.45	1077.46	1148.85	2002.37	1605.44	400.31	368.8	549.04	419.7	771.49
hsa-miR-455-3p	27.73	34.19	33.23	67.52	22.1	15.24	17.76	45.04	57.44	86.48	63.06	40.33	55.95	41.82	46.39	14.85
hsa-miR-1225-5p	0	0	0	0	0	0	0	0	0	0	0	0	0	0	0	0
hsa-miR-193b	102.08	101.87	110.95	31.38	59.48	68.57	63.14	95.71	98.57	93.13	120.87	44.26	165.63	43.6	24.3	105.37
hsa-miR-140-5p	32.77	26.51	18.22	19.02	23.31	18.7	21.05	24.63	31.2	46.57	31.53	27.54	9.57	14.24	13.25	21.02
hsa-miR-30e	4602.4	4521.98	4334.49	2311.79	2682.49	2786.36	2640.2	6355.66	2855.81	5168.9	5888.37	2196.3	3259.56	4022.12	4610.09	6416.83
hsa-miR-28-5p	735.98	766.8	780.93	325.23	470.59	511.84	570.27	1159.09	557.4	585.41	578.06	206.55	627.91	427.13	322.51	865.65
hsa-miR-424	34.03	25.82	31.62	93.19	63.5	39.48	65.12	19.71	66.66	33.26	42.04	473.1	6.63	65.85	260.66	11.21
hsa-miR-638	0	0	0	0	0	0	0	0	0	0	0	0	0	0	0	0
hsa-miR-30c	700.69	683.77	700.53	484.99	509.57	574.87	506.47	528.52	534	665.24	980.08	359	389.41	773.28	766.51	643.42
hsa-miR-1202	0	0	0	0	0	0	0	0	0	0	0	0	0	0	0	0
hsa-miR-99a	652.8	604.23	593.87	851.11	588.74	459.2	547.25	384.96	712	153	149.77	688.5	1480.35	462.72	1055.88	1272.55
hsa-miR-342-3p	240.71	247	243.87	878.69	198.52	209.17	221.66	204.09	213.46	518.89	359.98	496.7	133.24	315.01	474.93	45.68
hsa-miR-100	1299.31	1271.26	1242.41	2409.74	825.44	723.08	943.21	2786.89	3438.03	964.6	893.37	2397.93	7463.57	3244.39	4473.13	6893.24
hsa-let-7e	187.78	207.92	208.5	385.14	240.32	351.85	457.13	434.22	448.9	153	107.73	228.19	417.38	594.42	580.95	317.79
hsa-miR-18a	41.59	43.26	42.88	85.59	3.62	4.16	0.66	33.78	17.73	39.91	47.3	6.88	0	40.04	4.42	3.92
hsa-miR-185	20.16	20.23	17.15	15.22	9.64	13.85	9.87	20.41	11.35	19.96	34.16	23.61	17.67	7.12	19.88	8.97
hsa-miR-193a-3p	10.08	6.98	13.94	2.02	2.41	2.08	4.6	16.19	7.8	33.26	47.3	18.69	10.31	10.68	15.46	1.68
hsa-miR-146a	1442.98	1388.48	1478.77	2033.16	861.61	2428.29	1043.19	838.18	785.75	3618.9	4737.49	1551.08	423.27	1951.44	3317.85	4151.69
hsa-miR-574-3p	195.34	168.15	159.72	129.33	85.2	114.28	67.75	128.08	156.73	159.66	136.63	297.04	83.92	123.69	86.15	55.77
hsa-miR-30a	5100.2	5066.2	5145.96	3358.81	8512.04	8854.31	8443.51	4213.41	4021.67	3412.67	3757.41	13534.85	15261.33	9576.56	23116.7	11476.77
hsa-miR-940	1.26	0	1.07	0.95	0	0.69	1.32	0.7	0	6.65	0	0.98	0.74	0	0	0
hsa-miR-130a	211.72	205.83	195.63	723.68	285.73	197.39	279.54	135.83	175.16	505.58	320.56	1011.11	122.93	441.37	1033.79	60.25
hsa-miR-155	766.23	753.55	797.01	737.95	354.85	273.58	275.6	273.06	194.31	698.5	756.74	347.2	76.56	523.23	254.03	79.03
hsa-miR-150*	3.78	1.4	0.54	2.85	0.8	0.69	1.32	2.82	2.13	0	0	0.98	0	0	2.21	0
hsa-miR-181a	16044.13	16175.43	16721.57	32513.48	9184.37	10116.24	12021.66	15741.7	32564.13	12466.57	10250.12	26573.98	9259.71	54327.96	17097.3	6605.43
hsa-miR-96	35.29	31.4	25.73	12.36	6.43	7.62	3.95	16.19	87.94	59.87	94.59	17.7	21.35	31.14	30.93	16.25
hsa-miR-144*	265.91	324.44	280.85	177.83	49.03	61.64	63.8	95.01	13.47	13.3	39.41	188.84	27.24	144.16	108.24	23.54
hsa-miR-301a	190.3	180.71	187.59	488.8	83.59	58.87	91.43	76.01	67.37	432.41	244.36	81.64	42.7	178.86	61.85	78.47
hsa-miR-148b	967.87	947.51	944.4	834.95	1212.45	1059	949.79	1040.86	668.03	1809.45	2262.33	299	309.91	826.67	459.46	933.46
hsa-miR-497	76.87	90.01	73.97	75.13	57.47	58.87	67.09	53.49	133.32	53.22	26.28	116.06	27.97	67.63	141.37	26.06
hsa-miR-486-5p	16896.05	16772.69	16517.9	11038.8	2320.81	2149.86	2112.03	3185.92	1765.82	1277.26	1292.76	13553.53	2380.63	3475.75	8420.53	1112.26
hsa-miR-425	512.92	537.25	494.18	653.31	152.71	180.77	156.54	368.07	286.5	485.62	402.02	121.96	83.92	335.47	143.58	150.77
hsa-miR-1308	NA	NA	NA	NA	NA	NA	NA	NA	NA	NA	NA	NA	NA	NA	NA	NA
hsa-miR-186	5669.82	5564.38	5714.64	2617.05	2510.09	2229.51	2833.58	7260.69	2755.1	4736.5	3857.26	3672.63	2701.58	2662.43	3262.62	6017.78
hsa-miR-301b	545.68	522.6	543.49	79.88	9.24	8.31	16.44	30.97	48.22	106.44	81.45	7.87	8.1	24.03	4.42	24.1
hsa-miR-1268	0	0	0	0	0	0	0	0	0	0	0	0	0	0	0	0
hsa-miR-140-3p	874.61	858.9	838.81	855.87	882.91	1006.36	1071.47	1678.47	1316.92	1709.66	1634.34	667.84	644.11	1034	607.46	982.79
hsa-miR-320a	1009.45	1045.2	1010.86	818.78	231.08	246.57	242.05	338.51	1059.49	545.5	515	224.25	539.58	423.57	296	225.31
hsa-miR-574-5p	107.12	80.24	78.25	30.43	10.45	17.32	9.21	55.6	23.4	46.57	52.55	13.77	22.08	39.15	15.46	24.94
hsa-miR-150	260.87	236.53	257.27	746.51	134.23	119.82	107.21	177.35	73.04	86.48	36.79	128.85	6.63	333.69	148	20.46
hsa-miR-590-5p	0	0	0.54	0	0	0	0	0	0.71	0	2.63	0.98	0	1.78	0	0.28
hsa-miR-17*	75.61	74.66	71.82	41.84	20.09	12.47	18.42	68.26	36.88	39.91	23.65	16.72	13.99	37.37	39.76	15.97
hsa-miR-374a	88.22	65.59	77.72	98.9	83.99	74.8	78.27	153.42	71.63	212.88	357.35	125.9	120.72	92.54	245.19	192.8
hsa-miR-151-3p	9098.94	9299.32	9060.24	4323.08	4715.16	4303.18	4342.45	6426.74	4887.56	9539.52	9653.66	2801.19	4475.64	4410.98	3492.36	6319.87
hsa-miR-197	23.94	23.72	17.15	5.71	8.04	3.46	6.58	10.56	17.02	26.61	23.65	22.62	9.57	5.34	6.63	15.97
hsa-miR-663	0	0	0	0	0	0	0	0	0	6.65	7.88	0	0	0	0	0
hsa-miR-142-5p	4865.79	5135.98	4954.08	5205.58	2079.68	1881.12	2418.54	2667.95	2024.66	3419.33	2393.71	4042.45	451.98	4172.5	3227.28	564.11
hsa-miR-660	160.05	170.94	155.97	258.66	130.21	126.75	111.82	206.91	139	392.49	441.43	169.17	73.61	143.27	117.07	178.79
hsa-miR-324-5p	18.9	25.82	18.22	19.97	7.23	8.31	5.92	8.45	7.8	13.3	21.02	5.9	2.94	9.79	17.67	3.64
hsa-miR-7	3.78	7.68	13.94	4.75	3.62	6.23	3.95	5.63	6.38	179.61	165.54	19.67	9.57	5.34	2.21	9.81
hsa-miR-1246	3.78	1.4	0.54	67.52	2.01	1.39	1.97	2.11	0	106.44	91.96	0	3.68	7.12	0	12.05
hsa-miR-582-5p	20.16	21.63	18.22	19.97	4.42	6.23	2.63	2.82	9.22	26.61	34.16	11.8	0	8.9	4.42	1.68
hsa-miR-361-5p	167.61	161.18	164.01	230.13	165.17	148.91	139.44	271.65	199.98	206.22	254.87	56.06	111.15	134.37	125.91	226.43
hsa-miR-34b*	2.52	0	0	0	0	0	0	0	0	0	0	0.98	0	0	0	0
hsa-miR-30e*	364.21	410.96	413.78	316.67	319.89	260.42	372.29	503.89	557.4	991.21	956.43	592.11	797.22	371.07	501.43	762.8
hsa-miR-18b	7.56	12.56	3.75	11.41	0.8	0.69	0	7.04	2.84	6.65	5.26	2.95	0	4.45	2.21	1.12
hsa-miR-199a-5p	143.67	146.52	114	556.51	317.48	250.03	340.71	340.62	341.11	26.61	34.16	723.9	11.04	649.59	941.01	10.37
hsa-miR-200b	108.38	96.29	100.23	275.78	309.04	267.35	231.53	163.27	7.09	13.3	28.9	247.86	27.24	887.18	658.27	217.18
hsa-miR-1274a	NA	NA	NA	NA	NA	NA	NA	NA	NA	NA	NA	NA	NA	NA	NA	NA
hsa-miR-214	6.3	6.28	12.86	101.75	34.16	43.63	34.2	27.45	44.68	6.65	7.88	198.68	7.36	135.26	123.7	1.4
hsa-miR-324-3p	6.3	8.37	8.58	10.46	4.42	5.54	4.6	4.93	4.96	13.3	7.88	11.8	0.74	8.9	4.42	1.4
hsa-miR-145	73.09	51.63	63.78	486.89	234.69	245.88	237.45	300.51	392.17	172.96	128.75	287.2	95.7	274.96	481.55	21.02
hsa-miR-338-3p	32.77	27.91	26.26	25.68	9.64	13.85	18.42	31.67	16.31	33.26	15.77	32.46	4.42	21.36	48.6	11.49
hsa-miR-452	247.01	237.93	262.63	73.22	305.02	270.12	278.89	283.62	109.21	6.65	13.14	9.84	55.95	47.16	28.72	8.97
hsa-miR-1280	1.26	0.7	0.54	0.95	0.8	0	0	0.7	0.71	0	0	0	0	0	0	0.28
hsa-miR-374b	94.52	99.08	88.97	115.07	157.94	122.59	177.59	147.79	115.59	232.83	189.18	122.95	219.37	156.61	145.79	117.14
hsa-miR-1181	0	0	0	0	0	0	0	0	0	0	0	0	0	0	0	0
hsa-miR-181b	1724.01	1786.88	1901.66	1881.01	404.68	446.73	515.02	852.25	1437.48	665.24	585.95	778	334.94	2470.22	779.76	321.15
hsa-miR-362-5p	28.99	30.7	46.63	75.13	77.56	68.57	75.64	85.15	207.08	53.22	44.67	12.79	134.71	24.92	11.04	26.62
hsa-miR-20a*	5.04	6.28	0.54	5.71	0.4	0	0.66	2.82	4.25	0	13.14	0	0	3.56	4.42	0.28
hsa-miR-454	80.66	72.56	87.37	161.66	48.22	33.94	30.91	22.52	27.66	139.7	78.83	59.01	30.92	98.77	61.85	28.02
hsa-miR-10a	8730.95	8917.66	8975.02	37838.88	24832.03	22915.65	25032.57	40221.29	35219.24	9379.86	9438.2	103551.75	17600	48688.1	131534.36	9004.81
hsa-miR-210	55.45	55.82	56.81	84.64	9.64	9.7	12.5	39.41	29.78	86.48	97.22	98.36	37.54	88.1	22.09	14.01
hsa-miR-221	483.93	504.46	534.91	2026.51	542.93	572.79	609.73	580.6	568.75	824.9	680.54	483.91	255.44	1066.04	622.92	541.42
hsa-miR-1275	0	0	0.54	0	0	0	0	0	0.71	0	0	0	0	0	0	0.28
[illegible]	[illegible]	[illegible]	[illegible]	[illegible]	[illegible]	[illegible]	[illegible]	[illegible]	[illegible]	[illegible]	[illegible]	[illegible]	[illegible]	[illegible]	[illegible]	[illegible]

hsa-miR-1278	0	0	1.07	0	0.4	0	0	0.7	0.71	0	0	0	0	0	0	1.12
hsa-miR-548c-3p	0	0.7	0	2.85	1.21	0.69	1.97	2.11	0.71	0	0	0.98	0.74	2.67	2.21	2.8
hsa-miR-1178	0	0	0	0	0	0	0	0	0	0	0	0	0	0	0	0
hsa-miR-220a	NA	NA	NA	NA	NA	NA	NA	NA	NA	NA	NA	NA	NA	NA	NA	NA
hsa-miR-1243	0	0	0.54	0	0	0	0	0	0	0	0	0	0	0	0	0
hsa-miR-631	0	0	0	0	0	0	0	0	0	0	0	0	0	0	0	0
hsa-miR-668	2.52	0.7	0	6.66	0	0.69	0	0	0.71	6.65	2.63	0.98	0	2.67	0	1.12
hsa-miR-603	0	0	0	0	0	0	0	0	0	0	0	0	0	0	0	0
hsa-miR-744*	1.26	2.09	1.07	1.9	3.62	2.08	1.32	2.82	3.55	19.96	2.63	0	0.74	2.67	0	1.4
hsa-miR-656	1.26	0	2.14	23.77	0	2.77	1.32	0	0	0	13.14	2.95	0	8.01	0	0.56
hsa-miR-146a*	0	2.09	0.54	0	0	0	0	0	0	0	0	0.98	0	0	0	2.8
hsa-miR-101*	3.78	6.98	3.22	1.9	4.02	2.77	4.6	7.74	2.13	6.65	2.63	1.97	3.68	5.34	13.25	8.13
hsa-miR-148b*	73.09	79.54	75.04	46.6	27.73	45.02	40.12	89.38	41.84	86.48	89.34	24.59	22.82	51.61	35.34	30.83
hsa-miR-483-5p	0	0.7	0.54	142.64	0.4	0	0.66	0.7	0.71	0	2.63	2.95	9.57	148.6	19.88	0.56
hsa-miR-99a*	17.64	13.95	19.3	18.07	10.45	11.77	9.87	11.26	15.6	6.65	5.26	12.79	28.71	20.47	19.88	29.42
hsa-miR-876-5p	0	0	0	0	0	0	0	0	0	0	0	0.98	0	0	0	0
hsa-miR-92a-2*	0	0	0	0	0	0	0	0	0	0	0	0	0	0	0	0
hsa-miR-562	0	0	0	0	0	0	0	0	0	0	0	0	0	0	0	0
hsa-miR-1257	0	0	0	0	0	0	0	0	0	0	0	0	0	0	0	0
hsa-miR-1247	0	0	0	7.61	0.8	0	0.66	1.41	1.42	0	0	1.97	0	0	0	0
hsa-miR-647	0	0	0	0	0	0	0	0	0	0	0	0	0	0	0	0
hsa-miR-548i	0	2.09	0.54	0	0.8	0.69	0	1.41	0	0	2.63	0	0	0.89	2.21	4.48
hsa-miR-548g	0	0.7	0.54	0	0.4	0	0	0.7	0	0	0	0	0	0	0	0.84
hsa-miR-556-3p	8.82	9.77	9.65	0	1.21	2.77	1.32	4.22	1.42	13.3	10.51	0	1.47	1.78	0	3.36
hsa-let-7i*	1.26	2.09	3.75	9.51	2.81	1.39	1.97	2.82	0.71	6.65	0	14.75	0	3.56	6.63	0.56
hsa-miR-1204	0	0	0	0	0	0	0	0	0	0	0	0	0	0	0	0
hsa-miR-487b	8.82	2.79	2.68	183.54	5.63	4.16	8.55	2.82	2.84	39.91	73.57	3.93	0.74	98.77	6.63	2.24
hsa-miR-449b	5.04	4.19	2.14	0	0	0	0	1.41	0	0	0	0.98	0	0	0	0
hsa-miR-548j	0	2.09	1.07	0	0	1.39	0	2.82	0	0	2.63	0.98	0	0	0	0.28
hsa-miR-595	0	0	0	0	0	0	0	0	0	0	0	0	0	0	0	0
hsa-miR-100*	5.04	6.28	4.82	6.66	0.8	2.08	1.32	11.96	9.93	0	2.63	2.95	18.4	5.34	6.63	33.91
hsa-miR-380	0	0.7	0	20.92	0	0	0.66	1.41	0.71	6.65	13.14	0.98	0	9.79	0	0.28
hsa-miR-186*	0	0	0.54	0	0	0	0	0.7	0	0	0	0	0	0	0	0
hsa-miR-187*	0	0	0	0	0	0	0	0	0	0	0	0	0	0	0	0
hsa-miR-155*	0	0	0	0	0	0	0	0	0	0	0	0	0	0.89	0	0
hsa-miR-548o	20.16	18.14	21.44	10.46	10.45	11.08	7.24	17.59	0.71	26.61	18.39	1.97	4.42	6.23	2.21	14.01
hsa-miR-553	0	0	0	0	0	0	0	0	0	0	0	0	0	0	0	0
hsa-miR-541*	0	0	0	1.9	0	0	0	0	0	0	0	0	0	2.67	0	0.28
hsa-miR-504	0	0.7	2.14	0.95	0.4	0.69	0.66	0.7	0	0	0	2.95	0	0	0	9.25
hsa-miR-551a	0	0	0	0	0	0.69	0	0	0	0	0	0	0	0	0	0.28
hsa-miR-579	0	0	0.54	0	0	0	0	0	0	0	2.63	0	0	0	0	0.28
hsa-miR-1226*	0	0.7	0.54	0.95	0	0	0	0	0	0	0	0	0	0	0	0.28
hsa-miR-410	25.2	22.33	23.05	1713.64	27.73	28.4	41.44	34.48	32.62	365.88	336.33	56.06	7.36	777.73	35.34	14.29
hsa-miR-640	0	0	0	0	0	0	0	0	0	0	0	0	0	0	0	0
hsa-miR-302f	0	0	0	0	0	0	0	0	0	0	0	0	0	0	0	0
hsa-miR-105	1.26	0.7	1.61	0	0	0	0	1.41	0	6.65	5.26	0	0	0	0	0
hsa-miR-374a*	115.94	113.03	120.06	86.54	64.7	34.63	76.3	95.71	67.37	312.66	165.54	96.39	100.11	77.42	57.43	115.46
hsa-miR-649	0	0	0	0	0	0	0	0	0	0	0	0	0	0	0	0
hsa-miR-453	0	0	0	0	0	0	0	0	0	0	0	0	0	0	0	0
hsa-miR-302b*	0	0	0	0	0	0	0	0	0	0	0	0	0	0	0	0
hsa-miR-641	11.34	13.95	19.3	11.41	2.41	2.77	3.29	239.98	7.8	6.65	5.26	1.97	10.31	13.35	4.42	5.04
hsa-miR-10b*	0	0.7	0	0	0.4	0	0	2.82	3.55	19.96	5.26	0.98	0.74	0.89	2.21	6.17
hsa-miR-612	0	0	0	0	0	0	0	0	0	19.96	10.51	0	0	0	0	0
hsa-miR-450b-5p	23.94	25.82	20.9	32.33	23.31	19.39	25.65	16.89	21.98	0	10.51	41.31	0.74	40.04	70.69	11.21
hsa-miR-616*	10.08	14.65	19.83	0.95	3.62	4.85	5.92	2.82	2.84	13.3	2.63	0.98	2.94	2.67	0	12.05
hsa-miR-190b	15.12	25.82	19.3	3.8	6.83	13.85	10.52	20.41	2.13	6.65	7.88	2.95	2.21	1.78	2.21	15.69
hsa-miR-367*	0	0	0	0	0	0	0	0	0	0	0	0	0	0	0	0
hsa-miR-591	0	0	0	0	0	0	0	0	0	0	0	0	0	0	0	0
hsa-miR-1277	0	0	0	0	0.4	0.69	0	0.7	0	0	0	0.98	0	0.89	0	0.28
hsa-miR-129-5p	0	0	0.54	0.95	0	0	0	0	0	0	0	0	0	0	2.21	0
hsa-miR-106a*	0	0	0	0	0	0	0	0	0	0	2.63	0	0	0	0	0
hsa-miR-409-5p	0	0.7	1.07	56.11	2.01	0	1.32	2.11	0.71	19.96	23.65	1.97	0	26.7	6.63	0
hsa-miR-525-5p	1.26	0	0	0	0	0	0	16.89	0.71	6.65	0	0	0	0	0	1.68
hsa-miR-1914	0	0	0	0	0	0	0	0	0	0	0	0	0	0	0	0
hsa-miR-367	0	0	0	0	0	0	0	0	0	0	0	0	0	0	0	0
hsa-miR-1468	3.78	11.16	11.79	28.53	29.34	22.86	24.34	59.12	21.27	0	0	7.87	183.29	19.58	6.63	172.06
hsa-miR-1233	0	0	0	0	0	0	0	0	0	0	0	0	0	0	0	0
hsa-miR-103-as	0	0	0	0	0	0	0	0	0	0	0	0	0	0	0	0
hsa-miR-571	0	0	0	0	0	0	0	0	0	0	0	0	0	0	0	0
hsa-miR-1245	0	0	0	0	0	0	0	0	0.71	0	0	0	0	0	0	0
hsa-miR-302e	0	0	0	0	0	0	0	0	0	0	0	0	0	0	0	0
hsa-miR-525-3p	0	0	0	0	0	0	0	5.63	0	6.65	0	0	0	0	0	0.28
hsa-miR-488	0	0	0	0	0.4	0	0	0	0	0	0	0	0	0.89	0	0.28
hsa-miR-182	9093.9	8833.24	8700.6	1045.11	446.08	474.44	428.85	1287.18	15196.69	8135.87	10389.38	1268.8	2395.35	4049.7	1530.8	3951.04
hsa-miR-1197	0	0.7	0	38.99	0.4	0	0	0	0.71	0	15.77	0	0	8.01	0	0
hsa-miR-767-5p	5.04	4.19	4.29	0	0	0	0	22.52	2.84	33.26	36.79	0	0	0	0	0
hsa-miR-622	0	0	0	0	0	0	0	0	0	33.26	18.39	0	0	0	0	0
hsa-miR-1200	0	0	0	0	0	0	0	0	0	0	0	0	0	0	0	0
hsa-miR-592	2.52	0.7	3.22	0.95	0.8	0	0.66	0	0.71	6.65	2.63	1.97	1.47	0.89	0	1.68
hsa-miR-1184	0	0	0	0	0	0	0	0	0	0	0	0	0	0	0	0
hsa-miR-200c*	0	0	0	0	0	0	0	0	0	0	0	0	0	0	2.21	0
hsa-miR-566	0	0	0	0	0	0	0	0	0	0	0	0	0	0	0	0
hsa-miR-302a*	0	0	0	0	0	0	0	0	0	0	0	0	0	0	0	0
hsa-miR-1224-3p	0	0	0	0	0	0	0	0	0	0	0	0	0	0	0	0
hsa-miR-485-5p	0	0.7	0.54	18.07	0.8	1.39	1.97	0.7	1.42	0	2.63	0	0	24.92	2.21	0.56
hsa-miR-891b	0	0	0	0	0	0	0	0	0	0	0	0	0	0	0	0
hsa-miR-384	0	0	0	0	0	0	0	0	0	0	0	0	0	0	0	0
hsa-miR-539	0	0	0	8.56	0	2.08	0	0	0	6.65	5.26	0.98	0	11.57	2.21	0
hsa-miR-507	0	0	0	0	0	0	0	0	0	0	0	0	0	0	0	0
hsa-miR-548h	6.3	9.77	10.72	0.95	3.21	4.85	4.6	7.74	2.13	0	5.26	1.97	2.94	5.34	0	2.8
hsa-miR-512-5p	0	0	0	0	0	0	0	2.11	0.71	6.65	0	0	0	0	0	0.28
hsa-miR-325	0	0	0	0	0	0	0	0	0	0	0	0	0	0	0	0
hsa-miR-372	0	0	0	0	0	0	0	0.7	1.42	106.44	89.34	0	0	0	0	0.28
hsa-miR-518f*	0	0	0	0	0	0	0	12.67	0	0	0	0	0	0	0	0.28
hsa-miR-518c*	0	0	0	0	0	0	0	2.11	0	0	0	0	0	0	0	0
hsa-miR-642	0	0	0.54	0	0	0	0	0	0.71	0	0	0	0	0	0	0.28
hsa-miR-617	0	0	0	0	0	0	0	0	0	0	0	0	0	0	0	0
hsa-miR-1289	0	0	0	0	0	0	0	0	0	0	0	0	0	0	0	0
hsa-miR-516a-3p	0	0	0	0	0	0	0	0	0	0	2.63	0	0	0	0	0
hsa-miR-1909*	0	0	0	0	0	0	0	0	0	0	0	0	0	0	0	0
hsa-miR-1469	0	0	0	0	0	0	0	0	0	0	0	0	0	0	0	0
hsa-miR-765	1.26	0	0	0	0	0	0	0	0	0	0	0	0	0	0	0
hsa-miR-1301	27.73	42.56	34.3	10.46	3.62	2.08	3.95	6.33	7.09	6.65	7.88	2.95	3.68	6.23	2.21	8.69
hsa-miR-1911	6.3	6.28	6.43	0	0	0	0	0	2.13	0	0	0	0	0	0	0.28
hsa-miR-1263	0	0	0	0	0	0	0	0	0	0	0	0	0	0.89	0	0
hsa-miR-548n	0	2.09	2.14	0	1.21	0.69	0.66	2.11	1.42	0	0	0.98	1.47	0	0	0.84
hsa-miR-181c*	20.16	10.47	13.4	23.77	8.44	8.31	8.55	14.08	14.18	13.3	13.14	28.52	3.68	34.7	13.25	4.2
hsa-miR-330-3p	5.04	11.16	9.11	5.71	1.21	2.08	1.97	6.33	0.71	0	2.63	2.95	0.74	3.56	2.21	3.92
hsa-miR-369-3p	0	5.58	3.22	118.87	3.62	4.16	4.6	1.41	4.25	79.83	52.55	14.75	1.47	82.76	15.46	1.4
hsa-miR-1300	NA	NA	NA	NA	NA	NA	NA	NA	NA	NA	NA	NA	NA	NA	NA	NA
hsa-miR-618	15.12	11.86	13.94	4.75	9.64	9.7	6.58	5.63	7.09	13.3	15.77	5.9	5.15	4.45	0	3.92
hsa-miR-491-5p	1.26	0	0.54	0	0.8	1.39	0	1.41	0	0	0	0.98	0	0	2.21	0
hsa-miR-432	6.3	9.07	11.79	626.69	12.86	8.31	10.52	3.52	12.06	139.7	107.73	15.74	0.74	329.24	17.67	2.24
hsa-miR-518f	0	0	0	0	0	0	0	33.78	2.84	6.65	0	0	0	0	0	1.4
hsa-miR-623	0	0	0	0	0	0	0	0	0	0	0	0	0	0	0	0
hsa-miR-657	0	0	0	0	0	0	0	0	0	0	0	0	0	0	0	0
hsa-miR-1244	1.26	0	0.54	0.95	0.4	0	0.66	0	0	0	0	0	0	0	0	0
hsa-miR-1208	0	0	0	0	0	0	0	0	0	0	0	0	0	0	0	0
hsa-miR-1287	12.6	4.88	7.5	0	0.4	0	0	4.22	1.42	0	2.63	2.95	6.63	4.45	4.42	9.81
hsa-let-7g*	2.52	1.4	2.68	2.85	2.01	3.46	6.58	2.11	0	13.3	5.26	4.92	1.47	5.34	6.63	2.24
hsa-miR-147	0	0	0	0	0	0	0.66	0	0	0	0	0	0	0	0	0
hsa-miR-9*	0	0.7	0.54	0	0.4	0.69	0	0	0	0	0	0	0	0	2.21	0.56
hsa-miR-802	0	0	0	0	0	0	0	0	0	0	0	0	0	0	0	0
hsa-miR-1226	2.52	4.88	1.07	1.9	0.8	0.69	0	1.41	0	0	0	0	0.74	0	0	0
hsa-miR-578	0	0	0	0	0	0	0	0	0	0	0	0	0	0	0	0
hsa-miR-93*	25.2	16.75	17.69	13.31	10.05	12.47	11.18	17.59	23.4	26.61	13.14	0.98	2.94	4.45	8.84	18.5
hsa-miR-18a*	32.77	34.89	36.45	11.41	1.21	0	1.32	2.82	8.51	13.3	0	0.98	0	7.12	0	0.84
hsa-miR-105*	0	0	0	0	0	0	0	0	0	6.65	5.26	0	0	0	0	0
hsa-miR-382	1.26	0	1.07	80.83	1.21	3.46	3.29	2.82	1.42	19.96	31.53	7.87	0.74	46.27	4.42	0.28
hsa-miR-1265	0	0	0	0	0	0	0	0	0	0	0	0	0	0	0	0
hsa-miR-1915*	0	1.4	0	0	0	0	0	0	0.71	0	0	0	0	0	0	0
hsa-miR-345	781.35	734.01	766.99	194.95	112.12	118.44	88.14	434.92	497.12	139.7	186.56	62.95	139.13	207.33	152.42	442.49
hsa-miR-659	0	0	0	0	0	0	0	0	0	0	0	0	0	0	0	0
hsa-miR-1293	0	0	0	0	0	0	0	0	0	0	0	0	0	0	0	0
hsa-miR-338-5p	2.52	2.09	1.61	0.95	0.4	2.08	0.66	1.41	0	0	2.63	0	0	1.78	11.04	0.56
hsa-miR-632	0	0	0	0	0	0	0	0	0	0	0	0	0	0	0	0
hsa-miR-99b*	8.82	3.49	5.36	21.87	8.44	4.85	1.97	6.33	2.84	13.3	2.63	18.69	0	14.24	11.04	5.6
hsa-miR-506	0	0	0	0	0	0	0	0	0.71	0	0	0	0	0	0	0
hsa-miR-448	0	2.09	1.07	0	0	0	0	0	0.71	0	0	0	0	0	0	0
hsa-miR-486-3p	13.86	15.35	12.86	3.8	0.8	2.08	0	2.11	0	0	0	2.95	0	2.67	2.21	0.84
hsa-miR-330-5p	10.08	17.44	17.69	8.56	4.02	4.16	3.29	9.85	7.09	6.65	10.51	1.97	0.74	6.23	2.21	5.04
hsa-miR-607	0	0	0	0	0	0	0	0	0	0	0	0	0	0	0	0
hsa-miR-1250	0	0	0	0	0	0	0	0	0.71	0	0	0	0	0	0	0
hsa-miR-431*	0	0.7	0	13.31	0.4	0	0	0.7	0.71	6.65	2.63	2.95	0	8.01	0	0.28
hsa-miR-943	1.26	0.7	0.54	0	0	0	0	0.7	0.71	0	0	0	0	0.89	0	0
hsa-miR-708	1.26	0.7	0	10.46	2.81	0.69	2.63	0.7	4.25	0	2.63	5.9	0	8.9	11.04	0
hsa-miR-432*	0	0	0	0.95	0.8	0	0.66	0	0	0	5.26	0	0	0	0	0
hsa-miR-1302	1.26	0	0	0	0	0	0	0	0	0	0	0	0	0	0	0
hsa-miR-614	0	0	0	0	0	0	0	0	0	0	0	0	0	0	0	0
hsa-miR-548l	0	0	1.07	0.95	0.8	0	0	0.7	0.71	0	0	0	0	0	0	0
hsa-miR-200c	1.26	2.09	2.14	4.75	0.4	0.69	1.32	1.41	0.71	6.65	0	2.95	0	12.46	72.9	1.12
hsa-miR-222*	0	1.4	0	1.9	0	0.69	0	0.7	0	0	0	0	0	0.89	0	0
hsa-miR-1826	NA	NA	NA	NA	NA	NA	NA	NA	NA	NA	NA	NA	NA	NA	NA	NA
hsa-miR-513b	0	0	0	0	0	0	0	0	0	0	0	0	0	0	0	0
hsa-miR-200a*	2.52	1.4	2.68	7.61	5.63	1.39	5.26	2.82	0	0	5.26	12.79	0.74	16.02	19.88	2.8
hsa-miR-589*	3.78	2.09	3.22	1.9	1.61	0.69	1.32	4.93	2.84	0	0	0	0	0	2.21	0.84
hsa-miR-891a	5.04	6.98	1.61	0	0	0.69	0	0	2.84	0	15.77	0	0	0	0	19.62
hsa-miR-770-5p	0	0	0	0.95	0	0	0	0	0	0	0	0	0	0.89	0	0
hsa-miR-661	0	0	0	0	0	0	0	0	0	0	0	0	0	0	0	0
hsa-miR-658	0	0	0	0	0	0	0	0	0	0	0	0	0	0	0	0
hsa-miR-379	1.26	0.7	2.14	178.78	7.23	2.08	6.58	2.11	5.67	73.18	160.28	22.62	0	67.63	11.04	0.28
hsa-miR-431	5.04	4.88	9.65	234.89	8.44	11.08	7.89	2.82	3.55	79.83	78.83	2.95	1.47	360.39	2.21	2.52
hsa-miR-1322	0	0	0	0	0	0	0	0	0	0	0	0	0	0	0	0
hsa-miR-889	6.3	11.16	10.18	737	12.86	11.08	14.47	7.74	8.51	279.4	289.03	37.38	0	459.16	33.13	2.52
hsa-miR-646	0	0	0	0	0	0	0	0	0	0	0	0	0	0	0	0
hsa-miR-567	0	0	0	0	0	0	0	0	0	0	0	0	0	0	0	0
hsa-miR-662	0	0	0	0	0	0	0	0	0	0	0	0	0	0	0	0
hsa-miR-1913	0	0	0	0	0	0	0	0	0	0	0	0	0	0	0	0
hsa-miR-1269	1014.49	1030.54	1055.35	88.44	7.64	9.7	12.5	338.51	935.39	133.05	181.3	3.93	97.9	250.94	19.88	2784.98
hsa-miR-589	70.57	62.8	70.75	31.38	40.59	58.18	50.65	182.98	109.92	26.61	70.94	16.72	27.24	62.29	28.72	175.99
hsa-miR-219-2-3p	0	0	0	0	0	0	0	0	0	0	0	0	0	0	0	0
hsa-miR-300	0	0	0	0	0	0	0	0	0	0	0	0	0	0	0	0
hsa-miR-25*	25.2	27.91	26.8	4.75	1.61	0.69	0	4.93	6.38	0	5.26	0.98	0.74	0	0	11.21
hsa-miR-626	0	0	0	0	0	0	0	0	0	0	0	0	0	0	0	0
hsa-miR-220c	NA	NA	NA	NA	NA	NA	NA	NA	NA	NA	NA	NA	NA	NA	NA	NA
hsa-miR-935	0	0	0	0	0	0	0	0	0	0	0	0	0	0	0	0
hsa-miR-556-5p	15.12	20.93	19.3	0	2.81	2.08	4.6	4.22	3.55	0	0	0.98	0	0	0	3.92
hsa-miR-651	5.04	2.79	10.18	1.9	2.01	1.39	0.66	7.74	5.67	0	7.88	6.88	11.04	7.12	2.21	5.32
hsa-miR-653	5.04	5.58	4.29	1.9	0	0.69	0	4.93	3.55	0	0	0	0	0.89	0	2.52
hsa-miR-597	0	0	0	0	0	0	0	0.7	0	0	5.26	0	0	0	0	0
hsa-miR-629	6.3	6.28	6.97	2.85	1.21	1.39	1.97	9.15	0.71	46.57	47.3	10.82	0	20.47	4.42	14.57
hsa-miR-1270	1.26	1.4	5.9	2.85	0	0	0	1.41	4.96	0	2.63	0	0	0.89	0	4.76
hsa-miR-383	0	0	1.61	2.85	2.41	2.08	1.32	1.41	0.71	0	0	0	0	15.13	4.42	0.28
hsa-miR-515-5p	0	0	1.61	0	0	0	0	90.79	3.55	39.91	15.77	0	0	0	0	5.04
hsa-miR-137	0	0	0	0	0	0	0	0	0	0	0	0.98	0	0	0	0
hsa-miR-508-3p	0	0.7	0	0	0.4	0.69	0	0.7	0	0	0	0	0	0	0	0
hsa-miR-1262	0	0.7	0.54	0	0.8	0.69	0	0	0	0	0	0.98	0	0	0	4.48
hsa-miR-611	0	0	0	0	0	0	0	0	0	0	0	0	0	0	0	0
hsa-miR-411	102.08	108.85	84.15	2657.95	133.42	135.06	141.42	134.42	84.39	811.59	814.54	98.36	20.61	1120.32	130.33	57.45
hsa-miR-1254	8.82	4.88	8.04	0	0.4	0	1.32	1.41	0	0	2.63	0	0	0	2.21	1.68
hsa-miR-1910	0	0	0	0	0	0	0	0	0	0	0	0	0	0	0	0.28
hsa-miR-599	0	0	0	0	0	0	0	0	0	0	0	0	0	0	0	0
hsa-miR-10a*	2.52	4.19	2.68	9.51	4.02	2.77	3.95	6.33	4.96	0	0	14.75	3.68	4.45	28.72	1.68
hsa-miR-379*	0	0	1.07	28.53	0.8	2.77	0.66	0.7	0	26.61	28.9	2.95	0	9.79	0	0.28
hsa-miR-1304	0	0	0	0.95	0	0.69	0	0	0	0	0	0	0	0	0	0
hsa-miR-555	0	0	0	0	0	0	0	0	0	0	0	0	0	0	0	0
hsa-miR-205	3.78	5.58	6.43	0	0	0	0	0	0	26.61	7.88	0	0	0	0	0
hsa-miR-1201	NA	NA	NA	NA	NA	NA	NA	NA	NA	NA	NA	NA	NA	NA	NA	NA
hsa-miR-526b	0	0	0	0	0	0	0	48.56	4.25	26.61	10.51	0	0	0	0	5.32
hsa-miR-188-3p	0	0	0.54	1.9	1.21	1.39	1.32	0.7	2.13	6.65	0	0	1.47	0.89	0	0.28
hsa-miR-508-5p	0	0	0	0	0	0	0	0	0	0	0	0	0	0	0	0
hsa-miR-433	0	0	0	37.09	1.21	1.39	0	0.7	3.55	0	0	0.98	0.74	11.57	2.21	0.28
hsa-miR-760	3.78	4.19	0.54	0	0	0	0	0.7	0	0	0	0.98	0	0	0	0
hsa-miR-299-3p	1.26	3.49	0.54	85.59	2.81	2.08	1.97	2.82	2.84	26.61	70.94	10.82	0.74	66.74	6.63	0.56
hsa-miR-1276	3.78	4.88	2.68	0.95	0	0	0	2.82	2.84	0	0	0	0.74	1.78	0	0.56
hsa-miR-888*	0	0	0	0	0	0	0	0	0	0	0	0	0	0	0	0
hsa-miR-509-3p	0	1.4	2.68	4.75	2.81	4.85	5.26	7.04	2.84	0	5.26	0	0.74	1.78	2.21	3.08
hsa-miR-876-3p	0	0	0	0	0	0	0	0	0	0	0	0.98	0	0	0	0
hsa-miR-496	1.26	0	0	9.51	0	0	0.66	0.7	0	0	0	0	0	2.67	0	0.56
hsa-miR-558	0	0	0	0	0	0	0	0	0	0	0	0	0	0	0	0
hsa-miR-497*	0	1.4	0.54	0.95	2.81	1.39	1.97	2.11	0.71	0	2.63	2.95	0	4.45	2.21	0
hsa-miR-577	44.11	43.26	45.56	1.9	0	0	0	23.22	5.67	0	0	0.98	0	0	0	40.07
hsa-miR-363*	0	0	0	0	0	0	0	0	0	0	0	0	0	0	0	0
hsa-miR-518e*	0	0	0	0	0	0	0	26.04	0	0	2.63	0	0	0	0	1.96
hsa-miR-124	0	0	0	0	0	0	0	0	0	0	0	0	0	0	0	0
hsa-miR-302c*	0	0	0	0	0	0	0	0	0	0	0	0	0	0	0	0
hsa-miR-514	0	0.7	0	0.95	0.4	0.69	0.66	0	0	0	0	0	0	0	0	0
hsa-miR-644	0	0	0	0	0	0	0	0	0	0	0	0	0	0	0	0
hsa-miR-1253	0	0	0	0	0	0	0	0	0	0	0	0	0	0	0	0
hsa-miR-545*	0	0.7	1.61	0	0	0.69	0	0	2.13	0	0	0	0.74	1.78	0	0
hsa-miR-27b*	175.17	186.29	181.7	45.65	36.97	38.09	41.44	101.34	60.28	39.91	70.94	9.84	44.17	63.18	35.34	72.86
hsa-miR-633	0	0	0	0	0	0	0	0	0	0	0	0	0	0	0	0
hsa-miR-1183	0	0	0	0	0	0	0	0	0	0	0	0	0	0	0	0
hsa-miR-639	0	0	0	0	0	0	0	0	0	0	0	0	0	0	0	0
hsa-miR-654-5p	0	0	0	63.71	0.8	0.69	0.66	2.82	0	6.65	23.65	0.98	0	23.14	2.21	1.12
hsa-miR-490-5p	0	0	0	0	0	0	0	0	0	0	0	1.97	0	0	0	0
hsa-miR-148a*	365.47	394.22	383.23	136.94	285.33	297.13	278.23	732.61	569.46	525.54	714.7	73.77	930.46	143.27	136.96	2268.79
hsa-miR-552	1.26	0	0	0	20.09	15.24	17.1	11.26	4.96	0	28.9	0	187.71	1.78	2.21	638.1
hsa-miR-1306	1.26	0.7	0	1.9	0	0	0.66	1.41	0	0	0	0	0	0	0	0
hsa-miR-645	0	0	0	0	0	0	0	0	0	0	0	0	0	0	0	0
hsa-miR-296-3p	2.52	2.79	2.68	1.9	0	0.69	0	11.26	0	0	2.63	0	0	0	0	0
hsa-miR-106b*	360.43	338.4	356.43	202.56	116.54	99.04	126.95	258.28	309.2	153	168.16	154.42	91.28	133.48	88.36	141.8
hsa-miR-1273	0	0	0	0	0.4	0	0	0	0	6.65	7.88	0	0	0	0	0
hsa-miR-548p	39.07	39.07	41.27	2.85	7.23	4.16	7.24	13.37	26.24	6.65	10.51	1.97	12.51	4.45	2.21	0
hsa-miR-1203	0	0	0	0	0	0	0	0	0	0	0	0	0	0	0	0
hsa-miR-513a-5p	0	0	0	0	0	0	0	0	0	0	0	0	0	0	0	0
hsa-miR-513a-3p	0	0	0	0	0	0	0	0	0	0	0	0	0	0	0	0
hsa-miR-488*	0	0	0	0	0	0	0.66	0	0	0	0	0	0	0	0	0
hsa-miR-523	0	0	0	0	0	0	0	15.48	1.42	13.3	2.63	0	0	0	0	1.68
hsa-miR-1261	1.26	0	0	0	0	0.69	0	0	0	0	2.63	0	0	0	0	0.56
hsa-miR-604	0	0	0	0	0	0	0	0	0	0	0	0	0	0	0	0
hsa-miR-1285	35.29	32.1	27.34	3.8	7.23	11.08	6.58	23.93	8.51	6.65	13.14	2.95	6.63	6.23	0	14.57
hsa-miR-621	0	0	0	0	0	0	0	0	0	0	0	0	0	0	0	0
hsa-miR-1286	3.78	6.28	4.29	0	0	0	0	5.63	2.84	0	0	0	0	0.89	0	0
hsa-miR-490-3p	0	0.7	0	2.85	1.21	2.08	2.63	0.7	0	0	2.63	1.97	0	4.45	15.46	0
hsa-miR-141*	0	0	0	0	0.4	0	0	0	0	0	0	0.98	0	0	8.84	0
hsa-miR-888	0	0.7	0	0	0	0	0	0	0	0	0	0	0	0	0	0.84
hsa-miR-323-3p	0	0.7	0	130.28	0	2.08	1.32	0	0	46.57	15.77	1.97	0.74	55.17	2.21	0.28
hsa-miR-487a	1.26	0.7	2.68	158.81	1.61	0.69	1.32	0.7	2.13	13.3	31.53	0	0	57.84	2.21	0
hsa-miR-549	0	0	0	0	0	0	0	2.11	0.71	0	0	0	0	0.89	0	0.84
hsa-miR-493	6.3	6.98	9.11	252.96	10.85	13.16	9.21	9.15	9.22	73.18	165.54	16.72	1.47	218.01	24.3	3.64
hsa-miR-499-3p	0	0	0	0	0	0	0	0	0	0	0	0	0.74	0	2.21	0.56
hsa-miR-513c	0	0	0	0	0	0	0	0	0	0	0	0	0	0	0	0.56
hsa-miR-1205	0	0	0	0	0	0	0	0	0	0	0	0	0	0	0	0
hsa-miR-125b-1*	68.05	54.42	60.03	79.88	13.26	13.16	13.81	40.11	63.82	6.65	5.26	27.54	49.32	67.63	66.27	89.11
hsa-miR-184	1.26	1.4	4.29	0.95	0	0	0.66	2.82	2.84	6.65	10.51	0	9.57	3.56	4.42	309.1
hsa-miR-608	0	0	0	0	0	0	0	0	0	0	0	0	0	0	0	0
hsa-miR-412	0	0	0	0	0	0	0	0	0	6.65	0	0	0	0	0	0
hsa-miR-650	0	0	0	0	0	0	0	0	0	0	0	0	0	0	0	0
hsa-miR-564	0	0	0	0	0	0	0	0	0	0	0	0	0	0	0	0
hsa-miR-96*	0	0	0	0	0	0	0	0	0.71	0	0	0	0	0	0	0
hsa-miR-424*	20.16	20.93	24.12	42.79	16.48	16.62	15.79	13.37	21.27	0	2.63	56.06	0.74	29.37	33.13	10.09
hsa-miR-708*	0	0	0.54	3.8	0.8	0.69	0	2.11	0.71	0	0	2.95	0	1.78	2.21	0
hsa-miR-548f	0	2.09	3.22	0	0.8	0.69	0.66	1.41	0.71	0	0	0	0	0	0	2.24
hsa-miR-1182	0	0	0	0	0	0	0	0	0	0	0	0	0	0	0	0
hsa-miR-124*	0	0	0	0	0	0	0	0	0	0	0	0	0	0	0	0
hsa-miR-220b	NA	NA	NA	NA	NA	NA	NA	NA	NA	NA	NA	NA	NA	NA	NA	NA
hsa-miR-1471	0	0	0	0	0	0	0	0	0	0	0	0	0	0	0	0
hsa-miR-500	3.78	5.58	2.14	3.8	1.61	0.69	1.32	4.22	2.84	0	5.26	1.97	0.74	4.45	2.21	3.36
hsa-miR-1252	2.52	1.4	1.07	0	0	0	0	1.41	0.71	0	0	0	0	0	0	1.4
hsa-miR-573	0	0	0	0	0	0	0	0	0	0	0	0	0	0	0	0.28
hsa-miR-29b-2*	1.26	0.7	0	0.95	0.4	0	1.32	0	0	0	2.63	0.98	0.74	0	4.42	0.56
hsa-miR-147b	2.52	4.19	2.14	5.71	0.8	0.69	1.32	7.04	4.96	6.65	7.88	2.95	0	1.78	2.21	0.56
hsa-miR-583	0	0	0	0	0	0	0	0	0	0	0	0	0	0	0	0
hsa-miR-541	0	0	0	9.51	0	0	0.66	0	0	0	0	0	0	4.45	0	0
hsa-miR-297	0	0	0	0	0	0	0	0	0	0	0	0	0	0	0	0
hsa-miR-149*	0	0	0	0	0	0	0	0	0	0	0	0	0	0	0	0
hsa-miR-370	2.52	3.49	4.82	96.05	3.21	1.39	3.29	1.41	2.13	0	13.14	2.95	0	59.62	4.42	1.12
hsa-miR-643	0	0.7	0.54	0	0.8	0.69	0	1.41	0.71	0	0	0.98	0	0.89	2.21	0.84
hsa-miR-202	0	0	0	0	0	0	0	0	0	0	0	0	0	0	0	0
hsa-miR-518a-5p	0	0.7	0	0	0	0	0	9.15	0	0	5.26	0	0	0	0	0.28
hsa-miR-16-1*	0	0	0.54	0	0	0	0.66	0	0	0	2.63	0	0.74	0	0	0
hsa-miR-1180	17.64	17.44	21.44	53.25	2.01	0.69	3.95	6.33	7.09	6.65	0	5.9	0	2.67	0	3.36
hsa-miR-569	0	0	0	0	0	0	0	0	0	0	0	0	0	0	0	0
hsa-miR-146b-3p	386.89	427.01	412.71	164.52	426.39	359.46	357.16	33.78	39	212.88	173.42	51.15	28.71	78.31	97.19	72.86
hsa-miR-217	3.78	10.47	15.01	0	0.4	0	0.66	0.7	93.61	0	0	0	0	28.48	0	0.28
hsa-miR-568	0	0	0	0	0	0	0	0	0	0	0	0	0	0	0	0
hsa-miR-1297	0	0	0	0	0	0	0	0	0	0	0	0	0	0	0	0
hsa-miR-1272	0	0	0.54	0	0	0	0	0	0	0	0	0	0	0	0	0
hsa-miR-576-3p	3.78	2.09	4.29	0	4.42	3.46	2.63	2.11	2.13	13.3	2.63	1.97	1.47	0.89	0	1.12
hsa-miR-323-5p	0	0	0	0	0	0	0	0	0	0	0	0	0	0	0	0
hsa-miR-596	0	0	0	0	0	0	0	0	0	0	0	0	0	0	0	0
hsa-miR-200b*	0	0	0.54	0.95	1.61	0.69	1.97	0	0	0	0	3.93	0	0	17.67	1.12
hsa-miR-520a-5p	0	0	0	0	0	0	0	30.26	3.55	6.65	2.63	0	0	0	0	1.12
hsa-miR-28-3p	9538.76	10082.87	9892.62	2791.08	3233.46	2539.8	3111.15	6897.55	5749.2	4623.41	4351.24	1650.42	2624.28	2162.33	1778.21	6169.95
hsa-miR-34c-3p	2.52	2.79	1.07	0.95	0	0	0	0	0	0	0	0	0	0	0	0
hsa-miR-376b	0	0.7	0	29.48	0.4	0.69	0	0	0	26.61	26.28	3.93	0	7.12	2.21	0
hsa-miR-554	0	0	0	0	0	0	0	0	0	0	0	0	0	0	0	0
hsa-miR-517*	0	0	0	0	0	0	0.66	41.52	1.42	0	0	0	0	0	0	4.48
hsa-miR-1908	0	0	0	0	0	0	0	0	0	0	0	0	0	1.78	0	0
hsa-miR-663b	0	0	0	0	0	0	0	0	0	6.65	7.88	0	0	0	0	0
hsa-miR-20b*	0	0	0	0	0	0	0	0	0	0	0	0	0	0	0	0
hsa-let-7c*	NA	NA	NA	NA	NA	NA	NA	NA	NA	NA	NA	NA	NA	NA	NA	NA
hsa-miR-298	0	0	0	0	0	0	0	0	0	0	0	0	0	0	0	0
hsa-miR-610	0	0	0	0.95	0	0	0	0	0	0	0	0	0	0.89	0	0
hsa-miR-138-1*	0	0	0	0	0	0	0	0.7	0	0	0	0	0	0	0	0
hsa-miR-615-5p	0	0	0	0	0	0	0	0	0	0	0	0	0	0	0	0
hsa-miR-1266	5.04	4.19	4.29	0.95	0	0	0	2.11	0	6.65	5.26	2.95	0.74	0	0	1.12
hsa-miR-875-3p	0	0	0	0	0	0	0	0	0	0	0	0	0	0	0	0
hsa-miR-886-5p	NA	NA	NA	NA	NA	NA	NA	NA	NA	NA	NA	NA	NA	NA	NA	NA
hsa-miR-1258	0	0	0	0	0	0	0	0	0	0	2.63	0	0	1.78	0	0
hsa-miR-519e*	0	0	0	0	0	0	0	0	0	0	2.63	0	0	0	0	0
hsa-miR-648	0	0	0	0	0	0	0	0	0	0	0	0	0	0	0	0
hsa-miR-27a*	1.26	5.58	3.75	16.17	1.61	0.69	0.66	4.22	1.42	0	2.63	1.97	1.47	7.12	13.25	0
hsa-miR-1299	6.3	0	3.22	0	6.03	4.16	4.6	2.11	10.64	0	2.63	0	8.1	4.45	6.63	1.96
hsa-miR-600	0	0.7	0	0	0.4	0	0	0.7	0	0	0	0	0	0	0	0.84
hsa-miR-1827	0	0.7	0	0	0	0	1.32	0.7	0	0	0	0	0	0.89	0	1.4
hsa-miR-516b	0	2.09	3.22	0	0	0	0	418.03	23.4	6.65	13.14	0	0	0	0	38.95
hsa-miR-1255a	1.26	0.7	0	0	0.4	0	0	1.41	2.13	0	0	0	0.74	0.89	0	0
hsa-miR-1248	0	0.7	1.07	1.9	0.4	0	0	0	0	133.05	76.2	6.88	0	2.67	6.63	0.56
hsa-miR-1298	1.26	0	0	0	0	0	0	0	0	0	0	0	0	0	0	0
hsa-miR-1321	0	0	0	0	0	0	0	0	0	0	0	0	0	0	0	0
hsa-miR-138	0	0	1.07	1.9	0.8	0	0.66	0	0	6.65	10.51	6.88	0	8.01	0	0
hsa-miR-191	24983.02	26129.92	26337.09	37622.06	17987.36	22984.21	20204.05	35286.53	25895.85	24587.22	22678.48	7853.77	18244.84	11333.12	10700.17	16627.5
hsa-miR-628-3p	15.12	8.37	5.36	3.8	0.8	1.39	0.66	9.85	2.13	0	0	2.95	1.47	4.45	4.42	4.48
hsa-miR-331-5p	6.3	6.98	4.29	9.51	2.01	0	1.32	8.45	7.09	0	0	0	0.74	2.67	4.42	1.96
hsa-miR-510	0	0	0	0	0	0	0	0	0	0	0	0	0	0	0	0
hsa-miR-616	1.26	0.7	0.54	0	0.8	0	0	0	0.71	6.65	0	0	0	0	0	1.68
hsa-miR-153	6.3	7.68	7.5	4.75	4.42	0.69	1.32	2.82	2.13	0	0	3.93	2.21	7.12	4.42	4.76
hsa-miR-587	0	0	0	0	0	0	0	0	0	0	0	0	0	0	0	0
hsa-miR-33a*	0	0.7	0.54	0.95	2.01	0	1.32	2.11	2.13	6.65	2.63	0.98	3.68	0.89	2.21	0.28
hsa-miR-23a*	0	0	0	0	0	0	0	0	0	0	0	0	0	0	0	0
hsa-miR-580	1.26	4.19	0.54	1.9	2.01	0.69	3.29	2.11	0.71	6.65	7.88	0.98	0	0.89	0	4.76
hsa-miR-593*	0	0	0	0	0	0	0	0	0	0	0	0	0	0	0	0
hsa-miR-516a-5p	0	0.7	0	0	0	0.69	0	158.35	7.09	26.61	31.53	0	0	0	0	17.94
hsa-miR-302d*	0	0	0	0	0	0	0	0	0	0	0	0	0	0	0	0
hsa-miR-206	0	0	0	0	0	0	0	0	0	0	0	0	0	0	0	0
hsa-miR-219-1-3p	13.86	16.05	13.4	0	1.61	1.39	0.66	6.33	1.42	0	7.88	0	2.94	0.89	0	9.25
hsa-miR-675	5.04	2.09	4.82	0	0	0	0.66	0	0	0	0	1.97	0	0.89	2.21	0
hsa-miR-138-2*	0	0	0	0	0	0	0	0	0	0	0	0	0	0	0	0
hsa-miR-1283	0	1.4	0	0	0	0	0	66.15	2.13	0	7.88	0	0	0	2.21	10.37
hsa-miR-1251	0	0	0	0	0	0	0	0	0	0	0	0	0	0	0	0.56
hsa-miR-198	0	0	0	0	0	0	0	0	0	0	0	0	0	0	0	0
hsa-miR-606	0	0	0	0	0	0	0	0	0	0	0	0	0	0	0	0
hsa-miR-422a	0	2.09	2.14	0	1.21	0.69	0	2.11	1.42	6.65	0	1.97	3.68	0	0	0.84
hsa-miR-509-5p	0	0	0	0	0	0	0	0	0	0	0	0	0	0	0	0
hsa-miR-339-3p	98.3	87.22	90.58	24.73	26.52	38.09	34.86	111.19	51.77	33.26	47.3	7.87	21.35	23.14	30.93	64.17
hsa-miR-890	0	0	0	0	0	0	0	0	0	0	0	0	0	0	0	0
hsa-miR-936	0	0	0	0	0	0	0	0	0	0	0	0	0	0	0	0
hsa-miR-196a*	0	0	0	0	0	0	0	0	0	0	0	0	0	0	0	0
hsa-miR-183*	2.52	4.19	3.75	0.95	0	0	0	0	4.25	0	2.63	0	0	0.89	2.21	1.4
hsa-miR-218-1*	0	0	0	0	0	0	0	0.7	0	0	0	0	0	0	0	0
hsa-miR-380*	0	0	0	0	0	0	0	0	0	0	7.88	0	0	0.89	0	0
hsa-miR-1255b	0	2.09	1.61	0	0.4	0.69	0	1.41	0	0	2.63	0	0	0	0	0.28
hsa-miR-187	0	0	0.54	0	0.4	0	0.66	0	0.71	0	0	0.98	0	0	2.21	3.36
hsa-miR-934	0	0	0	0	0	0	0	0	0	0	0	0	0	0	0	0
hsa-miR-1292	1.26	0.7	2.14	0	0	0	0	0	0	0	0	0	0	0	0	0
hsa-miR-339-5p	21.42	20.93	18.76	24.73	7.23	6.93	8.55	15.48	26.24	39.91	31.53	8.85	6.63	12.46	4.42	10.65
hsa-miR-938	0	0	0	0	0	0	0	0	0	0	0	0	0	0	0	0
hsa-miR-620	0	0	0	0	0	0	0	0	0	0	0	0	0	0	0	0
hsa-miR-559	0	0	0	0	0	0	0	0	0	0	0	0	0	0	0	0
hsa-miR-1912	0	0	0	0	0	0	0	0	0	0	0	0	0	0	0	0
hsa-miR-1228*	0	0	0.54	0.95	0	0	0	0	0	0	0	0	0	0.89	0	0
hsa-miR-208a	0	0	0	0	0	0	0	0	0	0	0	0	0	0	0	0
hsa-miR-1291	0	2.09	0	0	0	1.39	0.66	0	0	13.3	10.51	0	0	0	0	1.96
hsa-miR-920	0	0	0	0	0	0	0	0	0	0	0	0	0	0	0	0
hsa-miR-1295	1.26	0	0.54	1.9	1.61	1.39	2.63	0	0	0	5.26	0	0	1.78	0	0
hsa-miR-208b	0	0	0	0	0	0	0	0	0	0	0	0	0	0	0	0
hsa-miR-922	0	0	0	0	0	0	0	0	0	0	0	0	0	0	0	0
hsa-miR-452*	0	0	0	0	0	0	0	0	0	0	0	0	0	0	0	0
hsa-miR-921	0	0	0	0	0	0	0	0	0	0	0	0	0	0	0	0
hsa-miR-509-3-5p	0	0	0	0	0	0.69	0	0	0	0	0	0	0	0	0	0
hsa-miR-588	0	0.7	0	0	0	0.69	0	0	0.71	0	0	0	0	0	0	0
hsa-miR-1909	0	0	0	0	0	0	0	0	0	0	0	0	0	0	0	0
hsa-miR-127-5p	1.26	2.09	3.22	67.52	4.42	2.77	6.58	2.11	2.13	13.3	15.77	14.75	0	31.14	11.04	0.84
hsa-miR-218-2*	0	0	0	0	0	0	0	0	0	0	0	0	0	0	0	0
hsa-miR-92b*	0	0	0	0	0	0	0	0	0	0	0	0	0	0	0	0
hsa-miR-135b*	0	0	0	0	0	0	0	0	0	0	0	0	0	0.89	0	0
hsa-miR-551b*	0	1.4	2.68	0	0	0	0	0	0	0	0	0	0	0	0	0
hsa-miR-941	273.47	281.88	285.68	86.54	240.72	200.86	192.72	165.38	104.96	172.96	186.56	49.18	72.88	48.94	33.13	352.26
hsa-miR-24-2*	7.56	14.65	10.18	7.61	7.64	7.62	7.89	8.45	5.67	0	5.26	4.92	2.94	8.9	8.84	4.48
hsa-miR-1231	0	0	0	0	0	0	0	0	0	0	0	0	0	0	0	0
