# Supplementary material for: Comparison of Hepatocellular Carcinoma miRNA Expression Profiling as Evaluated by Next Generation Sequencing and Microarray
Source: PLoS One. 2014 Sep 12;9(9):e106314. doi: 10.1371/journal.pone.0106314 (PMC4162537; doi:10.1371/journal.pone.0106314)
Supplement: Table S3 — Detailed mapping of short reads for novel miRNA candidates and hsa-mir-6715a. (PDF) [file pone.0106314.s007.pdf]

Table S3. Detailed mapping of short reads of novel miRNA candidates and hsa-mir-6715a

	sample ID	Sample ID	provisional id	miRDeep2 score	estimated probability that the miRNA candidate is a true positive	rfam alert	total read count	mature read count	loop read count	star read count	significant randfold p-value	miRBase miRNA	example miRBase miRNA with the same seed	UCSC browser	NCBI blastn	consensus mature sequence	consensus star sequence	consensus precursor sequence	precursor coordinate
hsa-miR-X																			
new12	A01_042513	K_023T	chrY_17662	1.4	0.56 ± 0.23		118	118	0	0	yes		ptr-miR-27a		blast	uucacaguggcuaagcuau	aguguucacugauggaua	uucacaguggcuaagcuauggaauccauccaaguguucacugauggaua	chrY:4474181..4474230:+
new12	B02_020613	CU_087T	chrY_18286	0.8	0.12 ± 0.14		180	180	0	0	yes		ptr-miR-27a		blast	uucacaguggcuaagcuau	aguguucacugauggaua	uucacaguggcuaagcuauggaauccauccaaguguucacugauggaua	chrY:4474181..4474230:+
new12	C03_040413	CU_091T	chrY_9666	0.8	0.01 ± 0.04		41	41	0	0	yes		ptr-miR-27a		blast	uucacaguggcuaagcuau	aguguucacugauggaua	uucacaguggcuaagcuauggaauccauccaaguguucacugauggaua	chrY:4474181..4474230:+
hsa-miR-Y																			
new12	A01_042513	K_023T	chr1_1641	5.7	0.87 ± 0.25		25	16	0	9	no				blast	uauggaggucucugucuggc	ucugaucguuccccuccguaca	uauggaggucucugucuggcuuaggacagcuggcuaagucugaucguuccccucguaca	chr1:175937535..175937595:-
new12	B02_020613	CU_087T	chr1_1819	1.0e+1	0.92 ± 0.18		35	25	0	10	no				blast	ucugaucguuccccuccguac	uauggaggucucugucuggc	uauggaggucucugucuggcuuaggacagcuggcuaagucugaucguuccccucguac	chr1:175937536..175937595:-
new12	C03_040413	CU_091T	chr1_805	0.1	0.01 ± 0.04		14	12	0	2	no				blast	uauggaggucucugucuggc	ucugaucguuccccuccgu	uauggaggucucugucuggcuuaggacagcuggcuaagucugaucguuccccucgu	chr1:175937538..175937595:-
new12	D04_020613	CU_089T	chr1_3321	3.3e+1	0.66 ± 0.48		79	57	0	22	no				blast	uauggaggucucugucuggc	ucugaucguuccccuccguac	uauggaggucucugucuggcuuaggacagcuggcuaagucugaucguuccccucguac	chr1:175937536..175937595:-
new11	D04	CU_087T	chr1_2909	2.7e+1	0.95 ± 0.15		70	45	0	25	no				blast	ucugaucguuccccuccguac	uauggaggucucugucuggc	uauggaggucucugucuggcuuaggacagcuggcuaagucugaucguuccccucguac	chr1:175937536..175937595:-
new11	A01	CU_083N	hr1_2685	7.8	0.83 ± 0.38		29	13	0	16	no				blast	ucugaucguuccccuccguac	uauggaggucucugucuggc	uauggaggucucugucuggcuuaggacagcuggcuaagucugaucguuccccucguac	chr1:175937536..175937595:-
new11	C03	CU_087N	chr1_3327	4.7	0.85 ± 0.22		23	8	0	15	no				blast	ucugaucguuccccuccguaca	uauggaggucucugucuggc	uauggaggucucugucuggcuuaggacagcuggcuaagucugaucguuccccucguaca	chr1:175937535..175937595:
new10	D04	K_181T	chr1_2081	8.3	0.86 ± 0.35		30	20	0	10	no				blast	uauggaggucucugucuggc	ucugaucguuccccuccguac	uauggaggucucugucuggcuuaggacagcuggcuaagucugaucguuccccucguac	chr1:175937536..175937595:-
new9	A01	K_175N	chr1_993	3.7	0.28 ± 0.45		21	17	0	4	no				blast	uauggaggucucugucuggc	ucugaucguuccccuccguac	uauggaggucucugucuggcuuaggacagcuggcuaagucugaucguuccccucguac	chr1:175937536..175937595:-
new9	D04	K_177T	chr1_3279	2.0e+1	0.89 ± 0.18		54	41	0	13	no				blast	uauggaggucucugucuggc	ucugaucguuccccuccguac	uauggaggucucugucuggcuuaggacagcuggcuaagucugaucguuccccucguac	chr1:175937536..175937595:-
new8	E05	K_177T	chr1_2029	1.2e+1	0.94 ± 0.20		39	30	0	9	no				blast	uauggaggucucugucuggc	ucugaucguuccccuccguaca	uauggaggucucugucuggcuuaggacagcuggcuaagucugaucguuccccucguaca	chr1:175937535..175937595:-
hsa-miR-6715a-3p																			
new12	A01_040413	CU_070T	chr10_1681	1.9e+1	0.57 ± 0.50		37	35	0	2	yes				blast	ccaaaccagucgugccugugg	acaggcacagccgguuugag	acaggcacagccgguuugagcauuuuuacugaauugccaaaccagucgugccugugg	chr10:114059382..114059439:+
new12	B02_020613	CU_087T	chr10_11773	1.9e+1	0.92 ± 0.18		36	34	0	2	yes				blast	ccaaaccagucgugccugugg	acaggcacagccgguuugagc	acaggcacagccgguuugagcauuuuuacugaauugccaaaccagucgugccugugg	chr10:114059382..114059439:+
new12	B02_040413	CU_087T	chr10_13147	2.4e+1	0.89 ± 0.23		46	42	0	4	yes				blast	ccaaaccagucgugccugugg	acaggcacagccgguuugag	acaggcacagccgguuugagcauuuuuacugaauugccaaaccagucgugccugugg	chr10:114059382..114059439:+
new12	B02_042513	CU_070T	chr10_3631	3.9e+1	0.44 ± 0.50		75	74	0	1	yes				blast	ccaaaccagucgugccugugg	cacaggcacagccgguuugagc	cacaggcacagccgguuugagcauuuuuacugaauugccaaaccagucgugccugugg	chr10:114059381..114059439:+
new10	A01	K_177N	chr10_4115	1.8	0.03 ± 0.08		13	13	0	0	yes				blast	ccaaaccagucgugccugugg	acaggcacagccgguuugagca	acaggcacagccgguuugagcauuuuuacugaauugccaaaccagucgugccugugg	chr10:114059382..114059439:+
new9	B02	K_175T	chr10_10081	3.8e+1	0.94 ± 0.18		74	73	0	1	yes				blast	ccaaaccagucgugccugugg	acaggcacagccgguuugagc	acaggcacagccgguuugagcauuuuuacugaauugccaaaccagucgugccugugg	chr10:114059382..114059439:+
hsa-miR-Z																			
new10	A01	K_177N	chr17_6186	1.4e+1	0.83 ± 0.25		20	13	0	7	yes		ptr-miR-548a		blast	aaaaacugugauuacuuuugc	agaaguaaucgcgguuuuug	agaaguaaucgcgguuuuugccauuacugcucauggcaaaaacugugauuacuuuugc	chr17:37453673..37453731:+
new10	B02	K_177T	chr17_35028	1.6e+1	0.87 ± 0.17		23	21	0	2	yes		ptr-miR-548a		blast	aaaaacugugauuacuuuugc	agaaguaaucgcgguuuuugc	agaaguaaucgcgguuuuugccauuacugcucauggcaaaaacugugauuacuuuugc	chr17:37453673..37453731:+
new11	B02	CU_083T	chr17_22022	0.5	0.07 ± 0.13		2	2	0	0	yes		ptr-miR-548a		blast	aaaaacugugauuacuuuugc	agaaguaaucgcgguuuuugc	agaaguaaucgcgguuuuugccauuacugcucauggcaaaaacugugauuacuuuugc	chr17:37453673..37453731:+
new11	D04	CU_087T	chr17_26356	1.0e+1	0.95 ± 0.15		11	8	0	3	yes		ptr-miR-548a		blast	aaaaacugugauuacuuuugc	agaaguaaucgcgguuuuug	agaaguaaucgcgguuuuugccauuacugcucauggcaaaaacugugauuacuuuugc	chr17:37453673..37453731:+
new12	A01_020613	CU_087N	chr17_22150	6.7	0.81 ± 0.39		4	3	0	1	yes		ptr-miR-548a		blast	aaaaacugugauuacuuuugc	agaaguaaucgcgguuuuug	agaaguaaucgcgguuuuugccauuacugcucauggcaaaaacugugauuacuuuugc	chr17:37453673..37453731:+
new12	B02_020613	CU_087T	chr17_16782	9.3	0.95 ± 0.12		9	5	0	4	yes		ptr-miR-548a		blast	aaaaacugugauuacuuuugc	agaaguaaucgcgguuuuugcc	agaaguaaucgcgguuuuugccauuacugcucauggcaaaaacugugauuacuuuugc	chr17:37453673..37453731:+
new12	B02_040413	CU_087T	chr17_18616	1.2e+1	0.89 ± 0.23		16	14	0	2	yes		ptr-miR-548a		blast	aaaaacugugauuacuuuugc	agaaguaaucgcgguuuuu	agaaguaaucgcgguuuuugccauuacugcucauggcaaaaacugugauuacuuuugc	chr17:37453673..37453731:+
new12	C03_020613	CU_089N	chr17_21816	3.1	0.48 ± 0.30		7	7	0	0	yes		ptr-miR-548a		blast	aaaaacugugauuacuuuugc	agaaguaaucgcgguuuuugc	agaaguaaucgcgguuuuugccauuacugcucauggcaaaaacugugauuacuuuugc	chr17:37453673..37453731:+
new12	C03_040413	CU_091T	chr17_8792	6.2	0.76 ± 0.43		3	2	0	1	yes		ptr-miR-548a		blast	aaaaacugugauuacuuuugc	agaaguaaucgcgguuuuug	agaaguaaucgcgguuuuugccauuacugcucauggcaaaaacugugauuacuuuugc	chr17:37453673..37453731:+
new12	D04_042513	O_086T	chr17_4628	1.6e+1	0.84 ± 0.21		23	20	0	3	yes		ptr-miR-548a		blast	aaaaacugugauuacuuuugc	agaaguaaucgcgguuuuugcc	agaaguaaucgcgguuuuugccauuacugcucauggcaaaaacugugauuacuuuugc	chr17:37453673..37453731:+
new12	E05	O_089T	chr17_21844	0.5	0.02 ± 0.06		2	2	0	0	yes		ptr-miR-548a		blast	aaaaacugugauuacuuuugc	agaaguaaucgcgguuuuugc	agaaguaaucgcgguuuuugccauuacugcucauggcaaaaacugugauuacuuuugc	chr17:37453673..37453731:+
new8	D04	K_177N	chr17_20142	0	0.23 ± 0.18		1	1	0	0	yes		ptr-miR-548a		blast	aaaaacugugauuacuuuugc	agaaguaaucgcgguuuuugc	agaaguaaucgcgguuuuugccauuacugcucauggcaaaaacugugauuacuuuugc	chr17:37453673..37453731:+
new9	C03	K_177N	chr17_33386	8.3	0.92 ± 0.16		7	4	0	3	yes		ptr-miR-548a		blast	aaaaacugugauuacuuuugc	agaaguaaucgcgguuuuugc	agaaguaaucgcgguuuuugccauuacugcucauggcaaaaacugugauuacuuuugc	chr17:37453673..37453731:+
new9	D04	K_177T	chr17_28472	1.6e+1	0.89 ± 0.18		23	22	0	1	yes		ptr-miR-548a		blast	aaaaacugugauuacuuuugc	agaaguaaucgcgguuuuugc	agaaguaaucgcgguuuuugccauuacugcucauggcaaaaacugugauuacuuuugc	chr17:37453673..37453731:+
hsa-miR-W																			
new10	A01	K_177N	chr2_850	0.8	0 +/- 0%	-	2	2	0	0	yes	-	ptr-miR-1294	-	-	ugugagguugucaug	aggcaugacaaccuca	ugugagguugucaug	chr2:133191304..133191365:+
new10	B02	K_177T	chr2_5330	1.9	79 +/- 8%	-	4	4	0	0	yes	-	ptr-miR-1294	-	-	ugugagguugucaug	aggcaugacaaccuca	ugugagguugucaug	chr2:133191304..133191365:+
new10	D04	K_181T	chr2_2834	0.3	15 +/- 16%	-	1	1	0	0	yes	-	ptr-miR-1294	-	-	ugugagguugucaug	aggcaugacaaccuca	ugugagguugucaug	chr2:133191304..133191365:+
new11	A01	CU_083N	chr2_3616	0.8	33 +/- 17%	-	2	2	0	0	yes	-	ptr-miR-1294	-	-	ugugagguugucaug	aggcaugacaaccuca	ugugagguugucaug	chr2:133191304..133191365:+
new12	A01_020613	CU_087N	chr2_3052	0.8	0 +/- 0%	-	2	2	0	0	yes	-	ptr-miR-1294	-	-	ugugagguugucaug	aggcaugacaaccuca	ugugagguugucaug	chr2:133191304..133191365:+
new12	C03_020613	CU_089N	chr2_3020	0.3	10 +/- 13%	-	1	1	0	0	yes	-	ptr-miR-1294	-	-	ugugagguugucaug	aggcaugacaaccuca	ugugagguugucaug	chr2:133191304..133191365:+
new12	D04_020613	CU_089T	chr2_4452	0.9	8 +/- 12%	-	2	2	0	0	yes	-	ptr-miR-1294	-	-	ugugagguugucaug	aggcaugacaaccuca	ugugagguugucaug	chr2:133191304..133191365:+
new12	D04_042513	O_086T	chr2_740	5.8	83 +/- 15%	-	14	14	0	0	yes	-	ptr-miR-1294	-	-	ugugagguugucaug	aggcaugacaaccuca	ugugagguugucaug	chr2:133191304..133191365:+
new12	E05	O_089T	chr2_3296	0.8	2 +/- 6%	-	2	2	0	0	yes	-	ptr-miR-1294	-	-	ugugagguugucaug	aggcaugacaaccuca	ugugagguugucaug	chr2:133191304..133191365:+
