# Supplementary material for: Comparison of Hepatocellular Carcinoma miRNA Expression Profiling as Evaluated by Next Generation Sequencing and Microarray
Source: PLoS One. 2014 Sep 12;9(9):e106314. doi: 10.1371/journal.pone.0106314 (PMC4162537; doi:10.1371/journal.pone.0106314)
Supplement: Text S1 — Sample script that processed short reads using fastx_clipper and miRDeep2. (PDF) [file pone.0106314.s008.pdf]

Text S1

Sample script for the execution of miRDeep2

Here is a sample script for the execution of miRDeep2.

fastx_clipper -Q 33 -c -v -i input.fastq -a ATGGAATTCTCGGGTGCCAAGGC -o trimmed_output.fastq

mapper.pl teimmed_output.fastq -h -g hsa -e -m -s trimmed_output_fastq.collapsed -p ./bowtie/indexes/hg19.ebwt/hg19 -t trimmed_output.fastq_vs_genome_h19.arf

miRDeep2.pl trimmed_output.fastq.collapsed hg19.fa trimmed_output.fastq_vs_genome_h19.arf mature_hsa.fa mature_ptr.fa hairpin_hsa.fa -t hsa

The detailed file explanations are:

input.fastq : input fastq file provided by NGS experiments.
trimmed_output.fastq : adapter trimmed fastq file
trimmed_output_fastq.collapsed : output file from mapper.pl
trimmed_output.fastq_vs_genome_h19.arf : another output file from mapper.pl
./bowtie/indexes/hg19.ebwt/hg19 : bowtie index files
hg19.fa : whole human genome sequence
mature_hsa.fa : human mature miRNA sequence (fasta format)
mature_ptr.fa : chimp mature miRNA sequence (fasta format)
hairpin_hsa.fa : human pre-miRNA sequence (fasta format)

Finally, miRDeeps generated huge number of files. For more details, see https://www.mdc-berlin.de/8551903/en/research/research_teams/systems_biology_of_gene_regulatory_elements/projects/miRDeep
