# Supplementary material for: Comparison of Hepatocellular Carcinoma miRNA Expression Profiling as Evaluated by Next Generation Sequencing and Microarray
Source: PLoS One. 2014 Sep 12;9(9):e106314. doi: 10.1371/journal.pone.0106314 (PMC4162537; doi:10.1371/journal.pone.0106314)
Supplement: Text S2 — Detailed description of feature extraction and discriminant procedures. (PDF) [file pone.0106314.s009.pdf]

Text S2

Detailed explanation of principal component analysis based feature extraction and linear discrminant analysis

Principal component analysis based feature extraction

Details of principal component analysis (PCA) based feature extraction is as follows. Suppose x_{ms} is mth miRNA expression of sth sample. There are two ways on how to apply PCA to x_{ms}.

1. Sample based PCA: Using PCA, each sample is embedded into low dimensional space. That is,

$$PC_s^k \equiv \sum_m C_m^k x_{ms},$$

where PC_s^k is the kth principal component score of sth sample.

2. miRNA based PCA: Using PCA, each miRNA is embedded into low dimensional space. That is,

$$PC_m^k \equiv \sum_s C_s^k x_{ms},$$

where PC_m^k is the kth principal component score of mth miRNA.

For PCA-based feature extraction, miRNA-based PCA was first applied to x_{ms} and M outlier miRNAs satisfying the following condition,

$$\sqrt{\sum_{k=1}^{K} (PC_m^k)^2} \geq D$$

where K is taken to be 2 if not declared explicitly, and D is taken to be as large as possible (this enables us to select as small number of miRNAs as possible) within the range where good enough performance is achieved for PCA based linear discriminant analysis (see the next section). Then, sample based PCA was applied using only selected M miRNAs in order to get

$$PC_s^{M,k} \equiv \sum_{m=1}^{M} C_m^{M,k} x_{ms}$$

that is used for the discrimination in the next section.

PCA based linear discriminant analysis

PCA based linear discriminant analysis (LDA) was performed using $PC_s^{M,k}$ obtained in the previous section. For this analysis, we employed semi-supervised machine learning work flame, i.e., although $PC_s^{M,k}$ was computed using all of samples, cross validations (leave one out cross validation) are performed by splitting samples into learning and test sets. The number of PCs, $M'(< M)$, used for LDA should be optimized so as to achieve the best performance in the cross validation. Here discriminant function is computed as,

$$LD_s^{M,M'} \equiv \sum_{k=1}^{M'} C_k^{M,M'} PC_s^{M,k}$$

which is used for discrimination between tumor and normal tissue.
